# Design, Synthesis, Biological Evaluation, and Molecular Docking Study of 4,6-Dimethyl-5-aryl/alkyl-2-[2-hydroxy-3-(4-substituted-1-piperazinyl)propyl]pyrrolo[3,4-*c*]pyrrole-1,3(2*H*,5*H*)-diones as Anti-Inflammatory Agents with Dual Inhibition of COX and LOX

**DOI:** 10.3390/ph16060804

**Published:** 2023-05-29

**Authors:** Aleksandra Redzicka, Benita Wiatrak, Izabela Jęśkowiak-Kossakowska, Andrzej Kochel, Remigiusz Płaczek, Żaneta Czyżnikowska

**Affiliations:** 1 Department of Medicinal Chemistry, Faculty of Pharmacy, Wroclaw Medical University, Borowska 211, 50-556 Wroclaw, Poland; aleksandra.redzicka@umw.edu.pl; 2 Department of Pharmacology, Wroclaw Medical University, Mikulicza-Radeckiego 2, 50-345 Wroclaw, Poland; benita.wiatrak@umw.edu.pl (B.W.); izabela.jeskowiak@umw.edu.pl (I.J.-K.); 3 Faculty of Chemistry, University of Wroclaw, ul. F.J oliot-Curie 14, 50-383 Wroclaw, Poland; andrzej.kochel@uwr.edu.pl; 4 Faculty of Pharmacy, Wroclaw Medical University, Borowska 211a, 50-556 Wroclaw, Poland; remigiusz.placzek@gmail.edu.pl; 5 Department of Basic Chemical Sciences, Faculty of Pharmacy, Wroclaw Medical University, Borowska 211a, 50-556 Wroclaw, Poland

**Keywords:** pyrrolo[3,4-*c*]pyrrole, cyclooxygenase inhibition COX-1/COX-2, LOX, molecular docking, analgesic activity, anti-inflammatory, cyclic imides

## Abstract

In the present study, we characterize the biological activity of a newly designed and synthesized series of 15 compounds 2-[2-hydroxy-3-(4-substituted-1-piperazinyl)propyl] derivatives of pyrrolo[3,4-*c*]pyrrole **3a**–**3o**. The compounds were obtained with good yields of pyrrolo[3,4-*c*]pyrrole scaffold **2a**–**2c** with secondary amines in C_2_H_5_OH. The chemical structures of the compounds were characterized by ^1^H-NMR, ^13^C-NMR, FT-IR, and MS. All the new compounds were investigated for their potencies to inhibit the activity of three enzymes, i.e., COX-1, COX-2, and LOX, by a colorimetric inhibitor screening assay. In order to analyze the structural basis of interactions between the ligands and cyclooxygenase/lipooxygenase, experimental data were supported by the results of molecular docking simulations. The data indicate that all of the tested compounds influence the activity of COX-1, COX-2, and LOX.

## 1. Introduction

**Figure 1:**
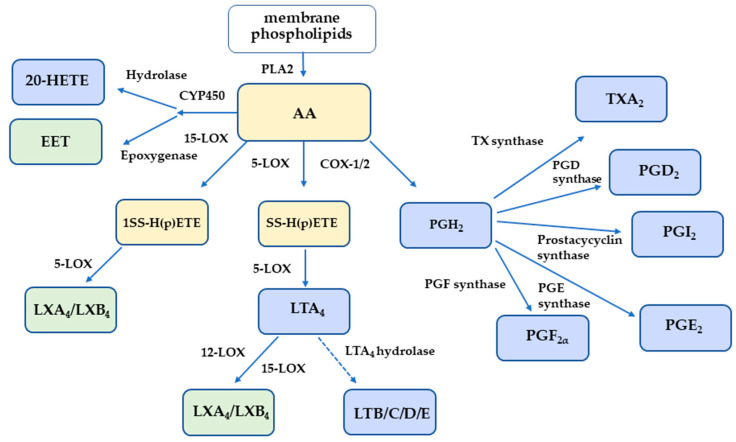
Mediators of inflammation released from arachidonic acid (AA) (pro-inflammatory metabolites are presented as blue, anti-inflammatory as green tiles) [2]. Abbreviations mean respectively, 20-HETE, 20-hydroxyeicosatetraenoic acid; EET, epoxyeicosatetraenoic acid; H(p)ETE, hydroperoxyeicosatetraenoic acid; LX, lipoxin; LT, leukotriene; PG, prostaglandin; PLA, phospholipase A; TX, thromboxane.

The phenomenon of inflammation is responsible for the initiation and/or progression of many disorders, such as asthma, rheumatoid arthritis, cardiovascular diseases, diabetes, Crohn’s disease, multiple sclerosis, neurodegenerative conditions (Alzheimer’s disease), and even cancer [1]. A key step in inflammation is the activation of cyclooxygenases (COX) and lipoxygenases (LOX) responsible for the production of inflammatory mediators from arachidonic acid (Figure 1) [2].

Three isoforms of COX enzymes, COX-1, COX-2, and COX-3, have been identified [3]. COX-1 is a constitutive enzyme expressed mainly in the kidneys, gastric, and lung mucosa, as well as on platelets [4]. Moreover, induced by inflammatory factors, COX-1 is expressed in all tissues. It is responsible for the synthesis of prostaglandins, which protect the gastric mucosa and regulate platelet aggregation and renal blood flow. COX-2 is induced during inflammation, pain, and oncogenesis and can be found in many cell types, such as the brain, kidney, and endothelial cells, as well as reproductive tissues, inflamed tissues, and tumor cells [4,5,6]. It also plays an important role in cancer progression and resistance radiotherapy and chemotherapy, as indicated by the overexpression of COX-2 in various types of cancer, including human prostate, breast, colorectal, and melanoma. Moreover, COX-2 inhibitors have shown both chemopreventive and anti-cancer effects through synergistic or additive effects with anti-cancer agents and can restore normal apoptosis in many types of cancer cells or inhibit angiogenesis [7].

Most of the classic nonsteroidal anti-inflammatory drugs (NSAIDs) that are widely used to treat inflammation inhibit both COX isoforms, so their long-term administration causes gastrointestinal ulceration and kidney and liver damage, mainly due to COX-1 inhibition [4,5]. On the other hand, there are several experimental studies indicating the positive effects of selective COX-1 inhibition. The increased level of COX-1 was found in many pathological conditions of the central nervous system, such as stroke, Alzheimer’s disease, and ischemic brain damage [8,9]. Some recent research has proved that COX-1 can be responsible for certain types of cancer progression [10]. Nevertheless, much attention is paid to designing selective COX-2 inhibitors to overcome the classic side effects associated with NSAIDs.

Although selective COX-2 inhibitors showed good anti-inflammatory activity with a good gastrointestinal and renal safety profile, cardiovascular side effects have been reported [11,12]. The second arachidonic pathway, on the other hand, involves lipoxygenases (LOXs), which exist as three different isozymes, i.e., 5-LOX, 12-LOX, and 15-LOX, and catalyze the production of inflammatory mediators from arachidonic acids (AA), such as leukotrienes, eoxins, and lipoxins [13].

**Figure 2:**
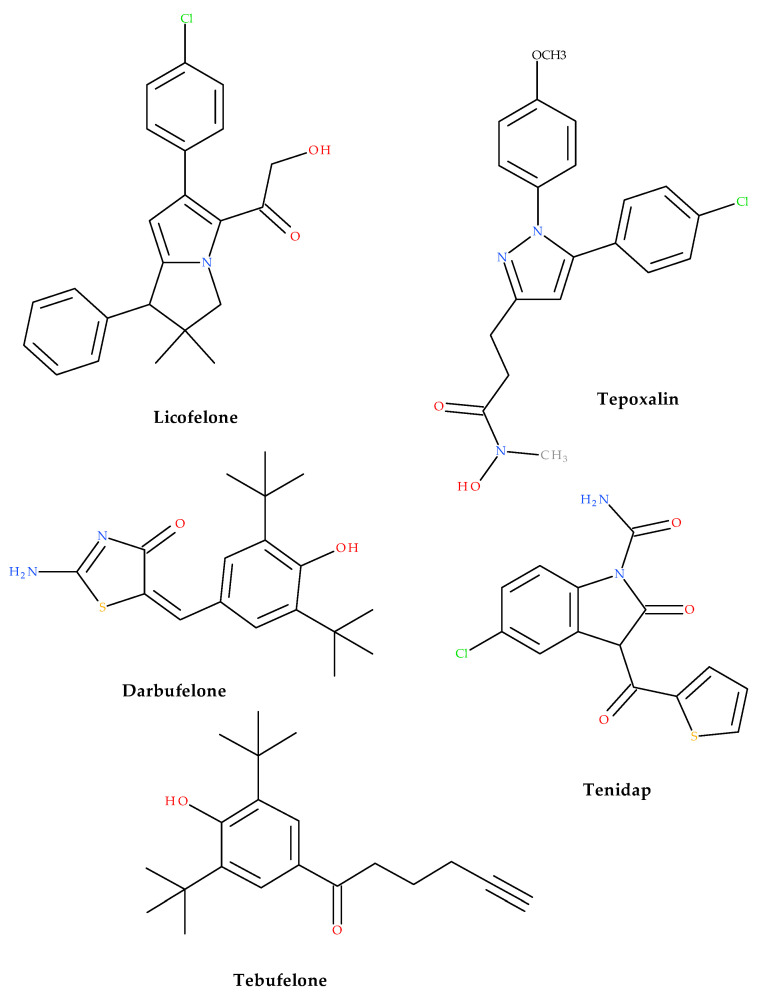
Structures clinically available COX and LOX inhibitors.

It is known that inhibition of COXs (by both NSAIDs and coxibs) leads to increased substrate availability and thus increased production of LOX inflammatory mediators due to arachidonic acid, which is the same substrate for both COXs and LOXs. Leukotrienes have been described as involved in cancer, cardiovascular disease, and gastrointestinal reactions induced by NSAIDs. Therefore, 5-LOX and 15-LOX represent promising therapeutic targets. The development of COX-1/COX-2 inhibitors with LOX inhibitory properties may be an important strategy to avoid gastrointestinal and cardiovascular side effects [14,15]. 15-LOX has been described as a target for the reduction of eoxin biosynthesis, a pro-inflammatory mediator, and tumor promoter [16,17]. Through the oxidation of low-density lipoprotein (LDL), 15-LOX leads to atherosclerosis [18,19]. In addition, 15-LOX is one of the key mediators in neurodegenerative diseases such as Alzheimer’s disease [20]. There are several clinically available COX and LOX enzyme inhibitors (Figure 2).

Therefore, the use of multi-targeted ligands (MTDLs) for dual inhibition of both 15-LOX and COX-2 enzymes is a promising strategy for drug therapy with maximum efficacy and minimal side effects [20,21]. Compounds that are dual COX/15-LOX inhibitors have potent anti-inflammatory [22,23,24,25,26,27] and anti-tumor [28] activities. They can prevent neurodegenerative diseases or be effective in cardiovascular diseases [14,20,29].

Dual activity against COX/LOX enzymes has been described for various heterocyclic systems [30,31,32,33]. Among them are compounds with a cyclic imide structure [34]. Commercially important drugs with anti-inflammatory properties and an imide structure include Apremilast, a drug used to treat certain types of psoriasis and psoriatic arthritis that also exhibits anti-inflammatory activity. This imide derivative may be useful in other immune-mediated inflammatory diseases [35]. Another class of drugs containing an imide group is immunomodulatory imide drugs (IMiDs). This class includes thalidomide and its analogs (lenalidomide [36] and pomalidomide [37]). Research on thalidomide and its analogs is intense due to its anti-angiogenic and anti-inflammatory properties [38]. It is worth noting here that the anti-inflammatory activity of thalidomide is also related to its ability to inhibit COX-2 [39,40,41]. The properties of imide derivatives are strongly dependent on the type of substituent in the imide ring. For example, the size and electrophilicity of the substituents affect their steric properties [42]. The presence of specific groups with nitrogen and oxygen atoms is responsible for the pharmacological consequences [43,44]. In addition, imide-based compounds are often neutral and hydrophobic, which determines their ability to penetrate biological membranes [45].

Earlier work showed that derivatives of 3,4-pyrroledicarboximides possessing the pharmacophore fragment 2-hydroxy-3-(4-phenylpiperazinyl)propyl exhibited strong analgesic activity in in vivo studies (Figure 2).

**Figure 3:**
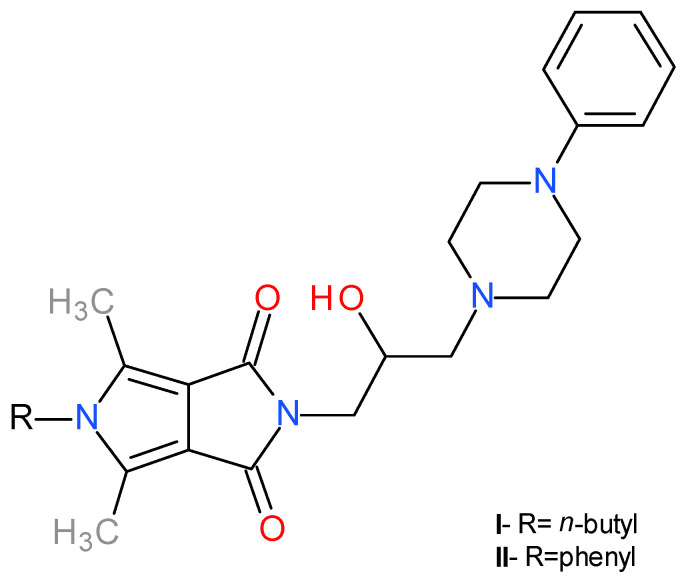
Pyrrolo[3,4-*c*]pyrroles with strong analgesic activity [41].

Compound **I** acted up to the dose of 1/80 LD_50_ and compound **II** to 1/40 (in the “writhing syndrome” test). In the “hot plate” test, Compound **I** and **II** acted up to the dose of 1/20 of LD_50_. Compounds **I** and **II** (Figure 3) were not tested in vitro for inhibitory activity against COX-1 and COX-2 enzymes [46].

The 2-hydroxy-3-(4-arylpiperazinyl)propyl fragment is described in the literature as a pharmacophore important for analgesic and anti-inflammatory activities [47,48,49].

Taking into account the above considerations, a series of pyrrolo[3,4-*c*]pyrrole derivatives were designed by modifying the pharmacologically active structures shown in Figure 1. The modifications consisted of leading substituents (F, Br, CF_3_, OC_2_H_5_) to the aryl ring by replacing the aryl ring with a heteroaryl or cyclohexyl ring. In addition, a methylsulfonyl group was introduced instead of the aryl ring at the N4 position of piperazine to increase the pharmacological activity and selectivity against the COX-2 enzyme. It is known that the substituted methylsulfonyl group is considered one of the pharmacophore molecules responsible for the selective recognition of key amino acid residues in the COX-2 active site pockets [50]. The methylsulfonyl group has previously been used in analgesics that are preferential (Nimesulide) or selective COX-2 inhibitors (Etoricoxib).

Despite significant progress in the design of dual COX/LOX inhibitors, the development of an effective and safe anti-inflammatory drug is still a significant challenge. Therefore, this paper reports the design and synthesis of a series of pyrrolo[3,4-*c*]pyrrole derivatives. Using a colorimetric screening assay, the new compounds were tested for their inhibitory activity against COX/LOX enzymes. The docking study against COX-2 and LOX active sites was established to determine the possible binding mode of new derivatives inside the site pocket in comparison to Meloxicam and Zileuton as a standard.

## 2. Results and Discussion

### 2.1. Chemistry

**Scheme 1:**
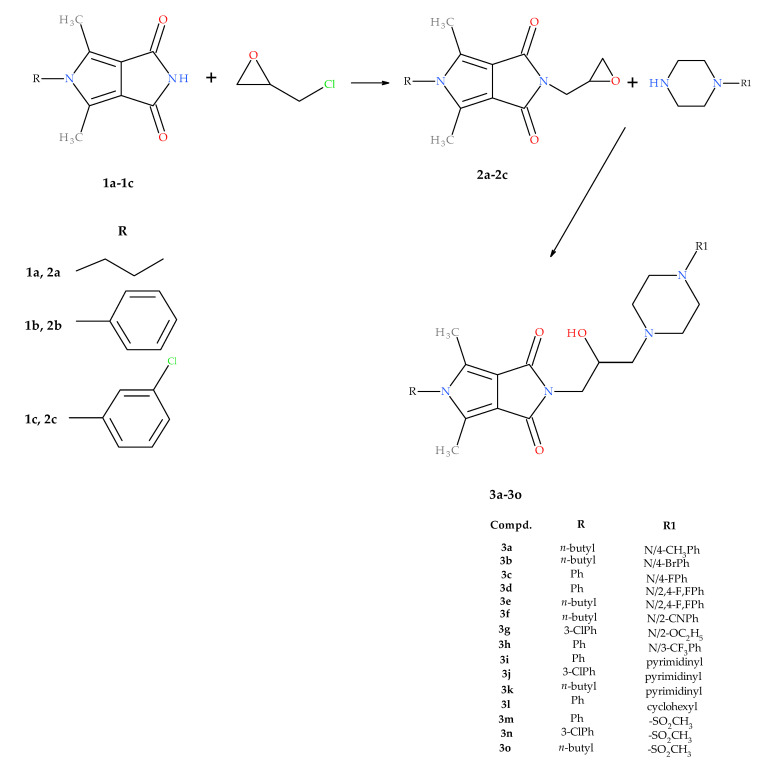
Synthetic route of the target compounds **3a**–**3o**.

The procedures for the preparation of target N-substituted pyrrolo[3,4-*c*]pyrroles **3a**–**3o** are shown in Scheme 1.

The final compounds **3a**–**3o** were generally synthesized by nucleophilic substitution of the corresponding derivatives of 1-aryl substituted piperazine **3a**–**3h**, heteroarylpiperazines **3j**–**3k**, cyclohexylpiperazine **3l**, and methylsulphonylpiperazines **3m**–**3o** with appropriate 3,4-pyrroledicarboximide intermediates **2a**–**2c**. The target compounds **3a**–**3o** were obtained with a 57–79% yield. The epoxide intermediates **2a**–**2c** were prepared by reactions of epichlorhydrin and pyrroledicarboximides **1a**–**1c** in the presence of NaH/DMF. The key intermediates, imides **1a**–**1c**, were prepared from ethyl α,β-diacetylsuccinate in a five-step synthesis according to known methods [51,52]. The analytical data (^1^H-NMR, ^13^C-NMR, FT-IR, and MS) of new products **2c** and **3a**–**3o** are in agreement with the assigned structures. The FT-IR spectra of all the new compounds revealed coupled imidic carbonyl absorption (1680 and 1750 cm^−1^) typical of five-member cyclic imides [53] and confirmed the presence of an OH group in their structure (3090–3490 cm^−1^). These spectra for **3m**–**3o** compounds confirmed the presence of the SO_2_ group (1320 cm^−1^). On the ^1^H-NMR spectrum, the proton present at the carbon atom bound to the hydroxyl group (in the linker connecting the pyrrolo[3,4-*c*]pyrrole core to the amine) was observed as a multiplet in the range of shift δ = 3.60–4.10 ppm. The signal of the carbon atom bound to the hydroxyl group (on the ^13^C NMR spectrum) was observed in the range of shift δ = 52.67–53.55 ppm. A detailed description of the spectra of the newly obtained compounds is provided in the Experimental Section and Supplementary Materials.

**Figure 4:**
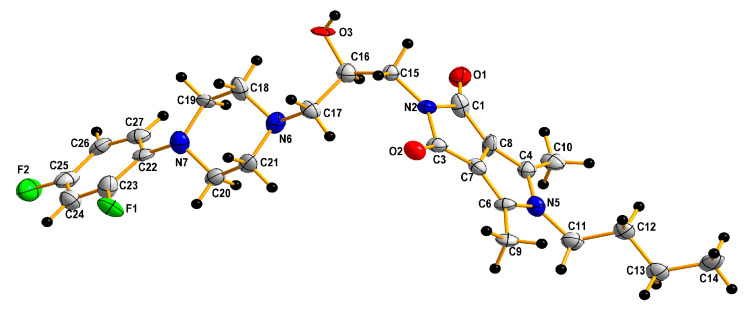
ORTEP diagram of the 3e compound. Displacement ellipsoids are plotted at the 50% probability level for non-H atoms.

Single-crystal X-ray diffraction data were measured on an XtaLAB Synergy R, DW system HyPix-Arc 150 κ-axis four-circle diffractometer with mirror-monochromated Cu Kα radiation (1.54184 Å). The X-ray diffraction data were collected at 100 K using an Oxford Cryosystem device. Data reduction and analysis were carried out with the CrysAlis Pro programs [54]. The needle, the very small size of the crystal, and the rotating-anode X-ray tube contributed to receiving weak final parameters. The structures were solved by direct methods and refined with the full-matrix least-squares technique using the SHELXT and SHELXL-2016 programs [55,56]. Crystal data and structure refinement for 3e are in Table 1 and Figure 4.

Crystallographic data for the structure reported in this paper have been deposited with the CCDC as file no. 2144853. (https://www.ccdc.cam.ac.uk/ accessed on 11 May 2023).

### 2.2. Biological Evaluation

#### 2.2.1. Cell Viability

Most of the tested compounds—presented no cytotoxic activity in the tested concentration range (Table 2). The compounds **3d** and **3o** are characterized by a lower IC_50_ value of 86.55 μM and 49.47 μM, respectively, against human dermal fibroblasts compared with the other receiving compounds. The remaining tested derivatives may present a pro-proliferative effect on Normal Human Dermal Fibroblast (NHDF) cells. The compound **3m** (563.49 μM) presented the highest IC_50_ value against NHDF from this series of derivatives.

#### 2.2.2. COX-1 and COX-2 Inhibition Assay Results

All receiving 4,6-dimethyl-5-aryl/alkil-2-[2-hydroxy-3-(4-substituted-1-piperazinyl)propyl]pyrrolo[3,4-*c*]pyrrole-1,3(2*H*,5*H*)-diones presented activity against COX-1 and COX-2 (Table 2). Only one compound, **3e** (0.64), is characterized by greater selectivity than that of Meloxicam (0.68). One compound, **3f,** had a selectivity equal to that of meloxicam. The rest of the series had lower selectivity than Meloxicam. It was observed that the introduction of an additional fluorine atom into the phenyl ring leads to an increase in activity against COX-2 (comparison of compounds **3c** and 3**d**). In addition, the replacement of the **3d** phenyl ring with a **3e** butyl group leads to an increase in activity against this COX isoform. For 3-chlorophenyl derivatives **3g**, **3j**, and **3n**, no effect of the substituent at the R^1^ position on activity against COX-2 was observed. Taking into account the Selectivity Ratio, it was observed that the replacement of the heteroaromatic ring **3h**–**3j** with a methylsulfonyl group **3m**–**3o** leads to a decrease in selectivity against COX-2. Compounds **3a**–**3d**, **3h**–**3m**, and **3o** indicated higher COX-1 inhibitory activity than Meloxicam. Only one compound from the series, **3e** (56.43 μM), had higher COX-2 activity than Meloxicam (57.14 μM). The remaining compounds in the series indicated lower inhibitory activity against COX-2 than that of Meloxicam. The compound **3o** (69.56μM) also has the highest COX-1 activity than Meloxicam (83.68 μM) and is the most active COX-1 compound in the series.

#### 2.2.3. LOX Inhibition Assay Results

All test compounds in the series presented inhibitory activity against 15-LOX (Table 2). Compounds **3c**, **3e**, **3f**, and **3g** had higher activity against LOX than Zileuton (13.37 μM) from the series of these compounds. The highest activity against 15-LOX from the series of these compounds is presented by the compounds **3c** (12.72 μM) and **3g** (12.80 μM).

### 2.3. Computational Studies

#### 2.3.1. The Analysis of Physicochemical and Pharmacokinetic Properties of Designed Compounds

It is a very important task to determine the drug-like properties of newly designed compounds as early as possible. Therefore, in the very early phase of drug development, in silico tools can be a promising alternative to complicated and expensive experimental studies and allow the selection of the best candidates. For example, biological studies determining the level of blood-brain distribution are extraordinarily time-consuming and difficult to access in extensive screening research on a large group of molecules. We should be aware that in the last stages of design, experimental analysis for promising compounds will be necessary.

Recently, two comprehensive revisions of web tools intended for the prediction of drug-like properties of compounds have been published [57,58]. Dulsat et al. performed a comparative evaluation of many different tools for determining these parameters for a set of 24 FDA-approved tyrosine kinase inhibitors. According to them, SwissADME offers the best coverage of physicochemical parameters for tested compounds and also offers the best accuracy and precision in the predictions. Additionally, it is extremely good at determining physicochemical and absorption properties. On the other hand, according to the authors, ADMETlab is the most complete tool in terms of the number of parameters that it can predict. Furthermore, some predicted parameters of tested drugs are close to experimental values.

In the present paper, the SwissADME and ADMETlab (2.0) sewers were used to determine the physicochemical and pharmacokinetic properties of the final compounds **3a**–**3o** (Table 3 and Table 4) [59,60]. In both cases, the calculations are performed based on experimental data obtained from the literature and the Drug Bank database.

The Lipinski and Veber rules are commonly used to evaluate drug-likeness and determine the chemical and physical properties of orally active compounds in humans. They are generally based on the assumption that the majority of drugs occupy a specific area of molecular properties. These physicochemical properties can affect the solubility and metabolic stability of biologically active compounds. The Lipinski rules of five focus on a set of parameters such as molecular weight (MW) ≤ 500 Da, lipophilicity values (log P) ≤ 5, number of hydrogen bond donors (NHD) ≤ 5, and number of hydrogen bond acceptors (NHA) ≤ 10.

Lipophilicity is the ability of a molecule to dissolve in a given environment and influences the form and method of drug administration as well as its pharmacodynamic and pharmacokinetic properties. The number of hydrogen donors and acceptors affects the polarity of the molecule and its lipophilicity, which is associated with the manner of intracellular drug distribution and transport. Veber rules include the number of rotatable bonds (NBR) ≤ 10 and the polar surface area (PSA) ≤ 140 Å^2^ [61,62]. The number of rotatable bonds and the size of the polar surface area of a molecule are considered as the main factors affecting drug absorption.

According to our study, the 10 tested compounds comply with Lipinski’s rule of five and contain less than five hydrogen bond donors, less than 10 hydrogen bond acceptors, with molecular weights below 500 Da, and log *p*-values < 5. Moreover, in line with the Veber rule, these molecules had less than 10 number of rotating bonds (NBR) and polar surface area (PSA) values lower than 140 Å^2^.

The obtained pharmacokinetic parameters of the chosen compounds were compared with the data available for meloxicam [63,64]. Results are presented in Table 4 and Table 5. According to the data, the designed compounds exhibit good bioavailability and good blood-brain barrier permeability. The plasma protein binding parameter does not exceed 96%, which may influence their good bioavailability. All, in contrast to meloxicam, exhibit favorable permeability parameters expressed as Caco-2 cell monolayers and high passive MDCK permeability. As is known, both models are experimentally used to assess the membrane permeability properties of compounds. The next important parameter is the affinity of newly designed drugs for membrane P-glycoprotein. This protein plays an important role in the transport of drugs out of the cells, reducing their concentration in the tissues. As presented in Table 4, compounds **3c** and **3h** exhibit a high probability of P-gp inhibition. The crucial role that cytochrome P450 plays in the metabolism of drugs and the type of products of its metabolic reactions give important information about the efficacy of toxicities and clearance of drugs. According to the results, all considered compounds exhibit a high probability of CYP2C19, CYP2C9, and CYP3A4 inhibition (Table 5).

#### 2.3.2. Molecular Docking

**Figure 5:**
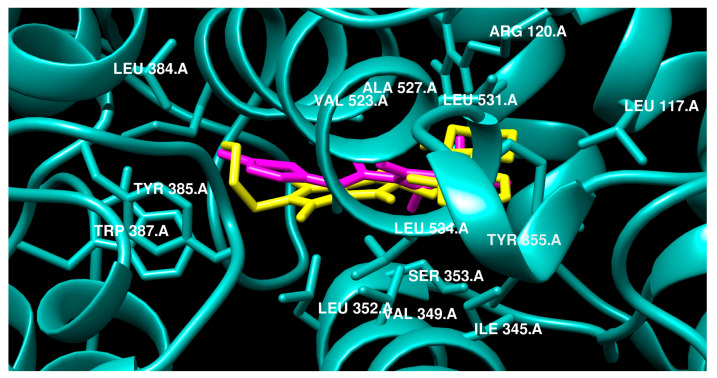
The binding mode of **3e** (yellow) and meloxicam (magenta) in the active center of COX-1.

**Figure 6:**
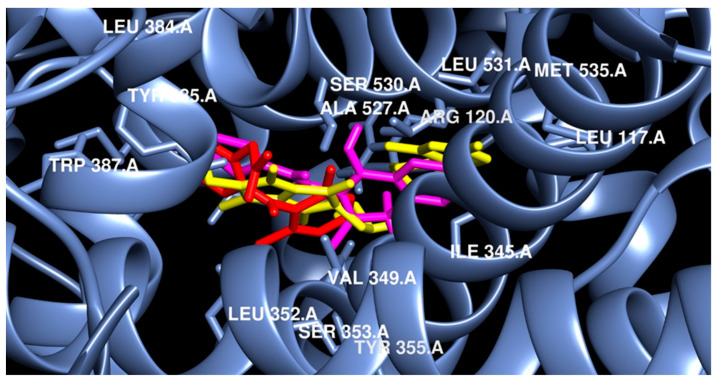
The binding mode of **3e** (yellow), meloxicam (magenta), and diclofenac (red) in the active center of COX-2.

**Figure 7:**
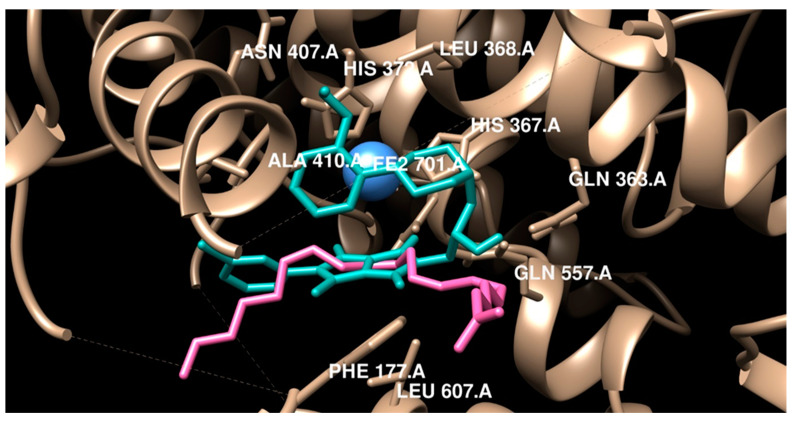
The binding mode of **3g** (corn blue) and arachidonic acid (green) in the active center of LOX.

In order to predict the binding mode of designed inhibitors to the binding site of cyclooxygenases, molecular docking was performed. In the present paper, we discuss in detail the impact of structural differences on the binding manner of most selective inhibitors of COX-2 according to enzymatic measurement results (Figure 5, Figure 6 and Figure 7 and Supplementary Materials). The obtained docking poses were compared with the positions of meloxicam and diclofenac.

**Figure 8:**
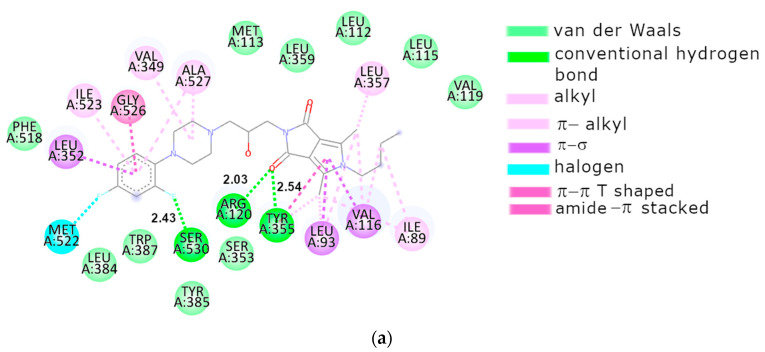

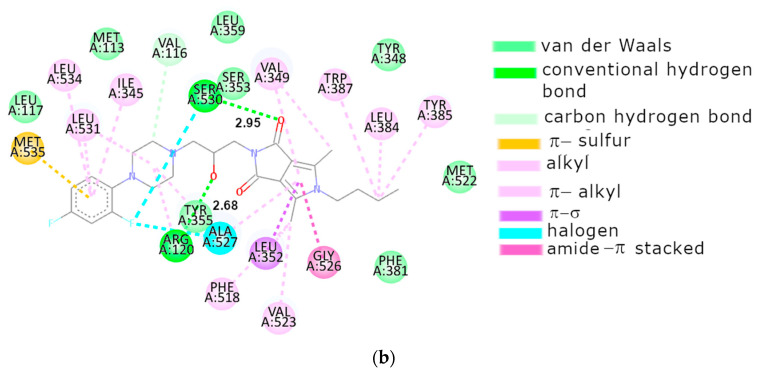
The intermolecular interactions of **3e** in the active center of (**a**) COX-1 and (**b**) COX-2. The distances between hydrogen atoms and hydrogen bond donors are given in Å.

Taking into account the scoring functions, including the free enthalpy of binding compounds, **3b**, **3d**, **3e**, **3h**, **3i**, **3l**, and **3o** can bind a little bit stronger to COX-2, which is consistent with the results of the experimental study. The scoring function, including the value of ΔG binding, which characterizes the affinity of protein-ligand complexes in this case, varies from −13.8 to −14.8 kcal/mol. In all cases considered, the potency of binding to the active center of COX-2 is similar, and the binding mode of compounds influences their selectivity. All inhibitor candidates can bind to a specific binding pocket of protein created by Leu352, Ser353, Tyr355, Phe518, and Val523, which arises as a result of Tyr355 conformation changes and the presence of Val523 instead of Ile523 in the structure of COX-2 [65]. Similar to classic nonsteroidal anti-inflammatory drugs such as diclofenac, designed inhibitors form hydrogen bonds with Arg120, Tyr355, and Ser530 [66,67]. As we can observe in Figure 5 and Figure 6, compound **3e**, for example, forms a hydrogen bond between oxygen atoms and arginine, tyrosine, and serine of COX-1 and arginine and serine of COX-2. Additionally, similar to meloxicam, all compounds interact with a hydrophobic pocket created by Ser353, Leu384, Tyr385, Trp387, Val523, and Met522. The pyrrolo[3,4-*c*]pyrrole part of molecule **3e** binds as meloxicam to the cavity of COX-2, including Val523, Gly526, Ala527, Ser530, and Leu531, which arises probably due to the conformation of Tyr 355. As presented in Figure 8b, compound **3e** can form two hydrogen bonds with Arg120 and Ser530 as classic anti-inflammatory agents. The phenylpiperazine moiety binds mainly through π—alkyl and van der Waals interactions with Ile345, Leu531, Leu534, Met113, Met117, and Tyr355, respectively. As determined, **3e** forms π—σ interactions with Leu93, Val116, and Leu352 of COX-1. Additionally, three hydrogen bonds are created with Arg120, Tyr355, and Ser530. In this case, **3e** takes a similar **3h** and **3i** orientation in the binding site of COX-1 (Figures S8a7, S3a, and S4a in Supplementary Materials). Data obtained for the remaining inhibitors are presented in Supplementary Materials. The intermolecular interactions of compound **3h** in the active center of COX-2 are presented in Figure S3b in Supplementary Materials. As can be seen, the most important factor responsible for protein-**3h** complex stabilization is van der Waals interactions. The pyrrolo[3,4-*c*]pyrrole moiety and its aromatic substituent interact with Ser353, Phe381, Leu384, Trp387, Phe518, Met522, Val523, Gly526, Ala527, and Ser530 amino acid residues. The alkyl and π—alkyl interactions are mainly created with phenylpiperazine fragments. The compound **3h** binds differently to the COX-1 enzyme. One hydrogen bond and π-lone interaction with Ser530 are present. Additionally, halogen interactions with Phe529 and Ser530 are created. The phenylpiperazine scaffold interacts via π—alkyl and alkyl interactions with Phe205, Phe209, Val344, Phe381, Tyr385, Trp387, and Leu534 amino acid residues.

The binding pose of **3i** in the active center of COX-2 exhibits some unfavorable interactions with Tyr355. Two conventional hydrogen bonds are presently created with Arg120 and Tyr355. Three π—σ interactions are possible with the Leu325 and Ala527 amino acid residues of the protein and the pyrrolo[3,4-*c*]pyrrole scaffold. Its aromatic substituent is exposed to Leu384, Trp387, Phe518, Met522, Val523, and Gly526 amino acids. As presented in Figure S4a in Supplementary Materials, **3i** can form two hydrogen bonds with Met522 and Ser530 of COX-1. In this case, the phenylpiperazine moiety interacts mainly through van der Waals interactions. The alkyl and π—alkyl interactions stabilize the pyrrolo[3,4-*c*]pyrrole scaffold and its aromatic substituent.

The binding mode of **3l** in the active center of COX-2 is presented in Figure S5b in Supplementary. As can be observed, the pyrrolo[3,4-*c*]pyrrole part of the molecule interacts with the protein via π—σ, alkyl, and amide—π stacked interactions with Leu352, Tyr385, Val349, Val523, Gly526, and Ala527. Two conventional hydrogen bonds are created with Ser530. The phenylpiperazine scaffold occupies the binding cavity in close proximity to Met113, Val116, Leu117, Arg120, Ile345, Tyr355, Leu359, and Leu531 amino acid residues. A similar binding mode of **3l** was obtained in the case of COX-1 binding (Figure S5a in Supplementary Materials).

In the case of interactions of the **3o** inhibitor in the active center of COX-2, the π—σ, alkyl, and van der Waals forces are crucial. In this case, the pyrrolo[3,4-*c*]pyrrole moiety of the inhibitor and its aliphatic substituent can penetrate hydrophobic pockets formed by Val349, Leu352, Phe381, Leu384, Tyr385, Trp387, Met522, Val533, and Gly526, similar to meloxicam. Similarly, diclofenac **3o** can form two hydrogen bonds with Tyr355 and Ser350 amino acid residues of COX-2. The piperazine ring interacts exclusively via van der Waals interactions with Met113, Val116, Leu117, Arg120, Leu359, Leu531, Leu534, and Met535. Similar binding modes and the nature of interactions were observed in the case of interactions with COX-1.

**Figure 9:**
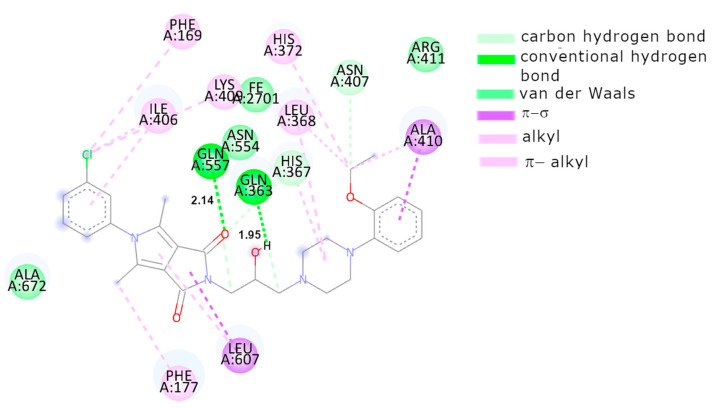
The intermolecular interactions of **3g** in the active center of LOX. The distances between hydrogen atoms and hydrogen bond donors are given in Å.

One of the mechanisms of lipoxygenase inhibition is the competition with its substrate in binding to the enzyme’s active site. According to an experimental study, **3c**, **3e**, **3f**, and **3g** pyrrolo[3,4-*c*]pyrrole derivatives presented inhibitory activity against LOX higher than the Zileuton reference drug. Therefore, we perform docking for the most potent compounds according to enzymatic measurements of the crystal structure of lipoxygenase. Below, we discuss in detail the result obtained for the most active inhibitor and compare it with the data characterizing the binding of arachidonic acid. Compounds **3g**, **3n**, and **3c**, according to scoring functions, including ΔG_binding_, were predicted to be the strongest LOX inhibitors. The pyrrolo[3,4-*c*]pyrrole moiety of compound **3g** binds exactly to the binding cavity occupied by arachidonic acid (Figure 7 and Figure 9). The phenylpiperazine scaffold forms interactions with two histidine, alanine, and arginine side chains. As can be observed, two conventional hydrogen bonds with Gln363 and Gln557 are formed. Additionally, a molecule of inhibitor interacts via many π—alkyl and π –σ interactions.

In the case of **3c** π—alkyl and π—π stacking interactions are important stabilizing factors of pyrrolo[3,4-*c*]pyrrole moiety in the protein binding cavity. Two conventional hydrogen bonds with Ala625 and Tyr558 are present.

In the case of **3e**, the most important stabilizing factors are van der Waals interactions. Similar to the previous ones, compound **3e** can form two hydrogen bonds with Tyr558 and Asn445. The pyrrolo[3,4-*c*]pyrrole scaffold interacts mainly via alkyl—π—alkyl and π–σ interactions.

A similar mode of binding exhibits **3n** compounds. Van der Waals interactions play a crucial role in the stabilization of the chlorobenzene group of inhibitors in the binding cavity formed by Asn554, Phe555, Gln557, and Tyr558. The piperazine ring is surrounded by Phe169, Ile406, Lys409, and Ala410. In this case, three hydrogen bonds are formed with Phe177, Gln413, and Ala672.

## 3. Discussion

Leukotrienes are a very important group of mediators that initiate the inflammatory response in diseases with inflammatory pathogenesis, including asthma, inflammatory bowel conditions, Alzheimer’s disease, allergic reactions, and COVID-19 [68,69,70]. The leukotriene mixture is a slow-reacting anaphylactic shock substance. Cysteine leukotrienes can be responsible for the persistence of allergic inflammation, bronchial smooth muscle contraction, and an increase in the permeability of blood vessels. Zileuton, selected as the reference drug in this study, is a 5-lipoxygenase inhibitor used in the treatment of asthma. It is administered for the prevention of asthma attacks due to its ability to inhibit inflammatory processes in the bronchi and prevent the formation of bronchial spastic conditions [71,72]. In addition, it also exhibits anti-angiogenic activity [73]. All tested compounds exhibit 15-LOX inhibitory activity. Increased 15-LOX activity has been reported in chronic sinusitis and asthma. Here, 15-LOX plays an important role in controlling the redox balance and epithelial homeostasis in asthma; therefore, the discoveries of 15-LOX inhibitors may pave the way for the creation of a new class of drugs used in the treatment of asthma [74].

Cyclooxygenase-1 produces prostaglandins that have a protective effect on the gastric mucosa and an effect on blood vessels. The COX-2 enzyme produces inflammatory mediators in damaged tissues. The anti-inflammatory, analgesic, antipyretic, and anticoagulant effects of NSAIDs result from blocking the COX-2 enzyme. On the other hand, the inhibition of physiological prostaglandin production by COX-1 blockade is the cause of most of the side effects of NSAIDs [75,76]. In addition, selective COX-2 inhibitors present chemopreventive activity in colorectal cancer [77]. Several years after the discovery of selective COX-2 inhibitors, some of them were withdrawn from the market due to cardiovascular side effects [78]. During the planned further animal studies of the obtained compounds, markers such as serum cardiac biomarker levels of AST, LDH, CK-MB, and ALP will be determined. At the same time, it is known that the risk of NSAID cardiovascular events increases with increasing COX-2 inhibitory power [79]. On the other hand, the results of some studies show that the increased cardiovascular risk associated with the initiation of treatment with diclofenac was greater than with selective COX-2 inhibitors (meloxicam/etodolac) and comparable to coxibs (celecoxib/etoricoxib) [80]. Therefore, it is necessary to look for multi-targeting compounds that are free from certain side effects.

Dual inhibition of COX-2 and LOX (mainly 5-LOX and 15-LOX) has become a research “hotspot” for the treatment of inflammatory diseases, and compounds with such properties are characterized by increased efficacy, reduced side effects, and a broader anti-inflammatory spectrum in comparison with classic NSAIDs [4,81,82]. Dual COX-2/LOX inhibitors showed better anti-cancer activity than their counterparts, which are inhibitors of one pathway [6]. At the same time, selective inhibition of both COX-2 and 15-LOX may provide a good strategy for alleviating inflammation while minimizing side effects during potential use in asthma [26]. There are some studies on new naproxen analogs with such properties [83]. According to the data, they are promising candidates for further development as anti-inflammatory agents without an increased risk of cardiovascular events.

Summing up, there are barely any studies in this area, and our work partially fills that gap.

We designed compounds in accordance with the latest requirements that can show inhibitory activity against COX-1, COX-2, and 15-LOX and could be an alternative to selective drugs. Molecular docking revealed that most of them can bind to enzymes similar to known drugs with a high value of free energy of binding. What is important is that most of them presented no cytotoxic activity in the tested concentration range. However, we should be aware that further preclinical studies are needed to evaluate the potential of 2-[2-hydroxy-3-(4-substituted-1-piperazinyl)propyl] derivatives as novel anti-inflammatory dual agents.

## 4. Materials and Methods

### 4.1. Chemicals and Instruments

All chemicals, reagents, and solvents used in the current study were purchased from commercial suppliers (Chemat, Gdańsk, Poland; Archem, Łany, Poland; Alchem, Wrocław, Poland) and used without further purification. Dry solvents were obtained according to the standard procedure. The progress of the reaction was monitored by the thin-layer chromatography (TLC) technique on silica-gel-60-F254-coated TLC plates (FlukaChemie GmbH, Buchs, Switzerland) and visualized by UV light at 254 nm. The melting points of received products were determined by an open capillary method on the Electrothermal Mel-Temp 1101D apparatus (Cole-Parmer, Vernon Hills, IL, USA) and were uncorrected. The ^1^H NMR (300 MHz) and ^13^C NMR (75 MHz) spectra were recorded on a Bruker 300 MHz NMR spectrometer (Bruker Analytische Messtechnik GmbH, Rheinstetten, Germany) in CDCl_3_ using tetramethylsilane (TMS) as an internal reference. Spectral data includes chemical shifts in ppm, multiplicities, constant couplings in Hz, number of protons, and protons positions. Multiplicities are abbreviated as follows: s (singlet), d (doublet), t (triplet), and m (multiplet). Spectra were recorded and read using TopSpin 3.6.2 (Bruker Daltonik, GmbH, Bremen, Germany). The infrared (IR) spectra were determined on a Nicolet iS50 FT-IR Spectrometer (Thermo Fisher Scientific, Waltham, MA, USA). Samples were applied as solids, and frequencies are reported in cm^−1^. Spectra were read using OMNIC Spectra 2.0 (Thermo Fisher Scientific, Waltham, MA, USA). Mass spectra were recorded using a Bruker Daltonics Compact ESI-mass spectrometer (Bruker Daltonik, GmbH, Bremen, Germany). The instrument was operated in positive ion mode. The analyzed compounds were dissolved in a mixture of chloroform and methanol.

#### 4.1.1. Synthesis of Compounds **2a**–**2c**

Synthesis and experimental data for compounds **2a** and **2b** were previously reported [46].

*1-(3-chlorophenyl)-2,5-dimethyl-N-(2,3-epoxypropyl)-3,4-pyrroledicarboximide* **2c**.

0.42 g of NaH (56–58% suspension in mineral oil) was added in portions to a solution of 0.01 mol of pyrroledicarboximide **1c** [45] in anh. DMF (15 mL). After stirring at room temperature for 1 h, 3.1 mL (0.04 mol) of epichlorohydrin was added, and stirring was continued at 60 °C for 5 h. The reaction mixture was then poured into cold H_2_O and the separated precipitate was filtered off and purified by crystallization from ethanol.

**2c**: Yield 80%, m.p. 140–142 °C.
**^1^H NMR** (300 MHz, CDCl_3_) δ: 1.58 (s, 6H, 2,5xCH_3_), 2.69–2.71 (m, 1H, CH_2_), 2.78–2.80 (m, 1H, CH_2_), 3.11–3.22 (m, 1H, CH-oxiran), 3.63–3.70 (dd, 1H, CH_2_-oxiran), 3.87–3.93 (dd, 1H, CH_2_-oxiran), 7.13–7.15 (m, 1H, ArH), 7.26–7.28 (m, 1H, ArH), 7.52–7.54 (m, 2H, ArH)
**^13^C NMR** (75 MHz, CDCl_3_) δ: 165.64, 130.92, 129.99, 128.21, 126.21, 49.48, 46.20, 39.14, 11.86
**FT-IR** (selected lines, γ_max_, cm^−1^): 1700 (C=O), 1760 (C=O)
**ESI-MS** (*m*/*z*): calcd. for C_26_H_37_N_4_O_3_ [M+H]^+^: 453.5970; found: 453.2871

#### 4.1.2. General Procedure for Preparation of 1-Substituted-2,5-dimethyl-N-[2-hydroxy-3-(4-substituted piperazine-1-yl)propyl]-3,4-pyrroledicarboximides **3a**–**3o**

A solution of 0.01 mol of epoxypropyl-pyrroledicarboximide **2a**, **2b [46]**, or **2c** and 0.01 mol of appropriate amine in absolute ethanol (50 mL) was refluxed for 3–5 h. The course of the reaction was controlled by TLC. The solvent was distilled off and the residue was purified by crystallization from ethanol (with charcoal) to give pure product **3a**–**3o**.

*4,6-dimethyl-5-butyl-2-[2-hydroxy-3-[4-(p-methylphenyl) piperazin-1-yl]propyl]pyrrolo[3,4-c]pyrrole-1,3(2H,5H)-dione (**3a**).*

**3a**: from **2a** and 1-(*p*-methylphenyl)piperazine. Yield 57%, m.p. 116–118 °C.
**^1^H NMR** (300 MHz, CDCl_3_) δ: 0.97 (t, 3H, CH_3_-butyl, *J* = 7.5 Hz), 1.35–1.42 (m, 2H, CH_2_-butyl), 1.60–1.65 (m, 2H, CH_2_-butyl), 2.26 (s, 3H, ArCH_3_), 2.39 (s, 6H, 4,6-CH_3_), 2.45 (d, 2H, CH_2_, H_γ_-propyl, *J* = 6.6 Hz), 2.58–2.60 (m, 2H, CH_2-piperazine_), 2.75–2.76 (m, 2H, CH_2-piperazine_), 3.10–3.20 (m, 4H, N(CH_2_)_2_Ar), 3.64–3.67 (m, 2H, N_pyrrole_-CH_2_), 3.72–3.77 (m, 2H, CH_2_H_α_-propyl), 398–4.02 (m, 1H, CH-H_β_-propyl), 6.81 (d, 2H ArH, *J* = 8.7 Hz), 7.04 (d, 2H, ArH, *J* = 8.4 Hz)
**^13^C NMR** (75 MHz, CDCl_3_) δ: 165.64, 149.16, 129.60, 129.20, 128.71, 116.37, 116.21, 65.84, 61.74, 53.32, 49.77, 43.83, 41.66, 32.46, 20.40, 20.02, 13.69, 11.37
**FT-IR** (selected lines, γ_max_, cm^−1^): 1700 (C=O), 1760 (C=O), 3110 (OH)
**ESI-MS** (*m*/*z*): calcd. for C_26_H_37_N_4_O_3_ [M+H]^+^: 453.5970; found: 453.2871

*4,6-dimethyl-5-butyl-2-[2-hydroxy-3-[4-(p-bromophenyl)piperazin-1-yl]propyl]pyrrolo[3,4-c]pyrrole-1,3(2H,5H)-dione* (**3b**).

**3b**: from **2a** and 1-(*p*-bromophenyl)piperazine. Yield 75%, m.p. 180–182 °C.
**^1^H NMR** (300 MHz, CDCl_3_) δ: 0.97 (t, 3H, CH_3_-butyl, *J* = 7.5 Hz), 1.34–1.42 (m, 2H CH_2_-butyl,), 1.57–1.64 (m, 2H, CH_2_-butyl,), 2.38 (s, 6H, 4,6-CH_3_), 2.45 (d, 2H, CH_2_ H_γ_-propyl, *J* = 6.6 Hz), 2.55–2.59 (m, 2H, CH_2-_piperazine), 2.73–2.76 (m, 2H, CH_2-_piperazine), 3.12–3.15 (m, 4H, N(CH2)2Ar), 3.65–3.67 (m, 2H, N_pyrrole_-CH_2_), 3.72 (t, 2H, CH_2_ H_α_-propyl *J* = 7.8 Hz), 3.98–4.02 (m, 1H, CH H_β_-propyl), 6.75 (d, 2H, ArH, *J* = 9 Hz), 7.30 (d, 2H, ArH, *J* = 9 Hz)
**^13^C NMR** (75 MHz, CDCl_3_) δ: 165.66, 150.26, 131.82, 128.76, 117.57, 116.19, 111.75, 66.06, 61.71, 53.13, 49.03, 43.83, 41.71, 32.46, 20.02, 13.68, 11.37
**FT-IR** (selected lines, γ_max_, cm^−1^): 1690 (C=O), 1760 (C=O), 3110 (OH)
**ESI-MS** (*m*/*z*): calcd. for C_25_H_34_BrN_4_O_3_ [M+H]^+^: 517.1814; found: 517.1789

*4,6-dimethyl-5-phenyl-2-[2-hydroxy-3-[4-(p-fluorophenyl)piperazin-1-yl]propyl]pyrrolo[3,4-c]pyrrole-1,3(2H,5H)-dione* (**3c**).

**3c**: from **2b** and 1-(*p*-fluorophenyl)piperazine. Yield 72%, m.p. 182–184 °C.
**^1^H NMR** (300 MHz, CDCl_3_) δ: 2.17 (s, 6H, 4,6–CH_3_), 2.49 (d, 2H, CH_2_ H_γ_-propyl, *J* = 6.9 Hz), 2.59–2.62 (m, 2H, CH_2_-piperazine), 2.77–2.78 (m, 2H, CH_2_-piperazine), 3.09–3.10 (m, 4H, N(CH_2_)_2_Ar -), 3.70–3.73 (m, 2H, CH_2_ H_α_-propyl), 4.03–4.07 (m, 1H, CH H_β_-propyl), 6.84–6.88 (m, 2H, ArH), 6.92–6.98 (m, 2H, ArH), 7.17–7.26 (m, 2H, ArH), 7.53–7.55 (m, 3H, ArH)
**^13^C NMR** (75 MHz, CDCl_3_) δ165.62, 158.74, 155.58, 147.94, 135.95, 130.06, 129.89, 127.83, 117.81, 117.71, 116.48, 115.62, 115.33, 65.88, 61.72, 53.32, 50.22, 41.82, 11.89
**FT-IR** (selected lines, γ_max_, cm^−1^): 1680 (C=O), 1750 (C=O), 3110 (OH)
**ESI-MS** (*m*/*z*): calcd. for C_27_H_30_FN_4_O_3_ [M+H]^+^: 477.5505; found: 477.2303

*4,6-dimethyl-5-phenyl-2-[2-hydroxy-3-[4-(2,4-difluorophenyl)-1-piperazinyl]propyl]pyrrolo[3,4-c]pyrrole-1,3(2H,5H)-dione* (**3d**).

**3d**: from **2a** and 2,4-difluorophenylpiperazine. Yield 69%, m.p. 143–145 °C.
**^1^H NMR** (300 MHz, CDCl_3_) δ: 2.20 (s, 6H, 4,6–CH_3_), 2.51–2.53 (m, 2H, CH_2_ H_γ_-propyl), 2.62–2.67 (m, 2H, CH_2-_piperazin_e_), 2.79–2.85 (m, 2H, CH_2_-piperazine), 3.00–3.15 (m, 4H, N(CH_2_)_2_Ar), 3.71–3.74 (m, 2H, CH_2_ H_α_-propyl), 4.01–4.10 (m, 1H, CH H_β_-propyl), 6.78–6.95 (m, 3H, ArH), 7.20–7.28 (m, 2H, ArH), 7.54–7.57 (m, 3H, ArH)
**^13^C NMR** (75 MHz, CDCl_3_) δ: 165.62, 135.97, 130.07, 129.89, 129.56, 127.84, 119.52, 116.48, 110.52, 105.01, 104.68, 65.87, 61.72, 53.36, 50.99, 41.80, 11.89
**FT-IR** (selected lines, γ_max_, cm^−1^): 1695 (C=O), 1750 (C=O), 3100 (OH)
**ESI-MS** (*m*/*z*): calcd. for C_27_H_29_F_2_N_4_O_3_ [M+H]^+^: 495.5410; found: 495.2221

*4,6-dimethyl-5-butyl-2-[2-hydroxy-3-[4-(2,4-difluorophenyl)piperazin-1-yl]propyl]pyrrolo[3,4-c]pyrrole-1,3(2H,5H)-dione* (**3e**).

**3e**: from **2a** and 2,4-difluorophenylpiperazine. Yield 76%, m.p. 139–141 °C.
**^1^H NMR** (300 MHz, CDCl_3_) δ: 0.97 (t, 3H, CH_3_-butyl, *J* = 7.2 Hz), 1.35–1.42 (m, 2H, CH_2_-butyl,), 1.57–1.65 (m, 2H, CH_2-_butyl), 2.39 (s, 6H, 4,6-CH_3_), 2.46 (d, 2H, CH_2,_ H_γ_-propyl_,_ *J* = 6.3 Hz), 2.60–2.62 (m, 2H, CH_2_-piperazine), 2.75–2.77 (m, 2H, CH_2_-piperazine), 3.00–3.10 (m, 4H, N(CH_2_)_2_Ar), 3.65–3.67 (m, 2H, N_pyrrole_-CH_2_), 3.72–3.77 (m, 2H, CH_2_ H_α_-propyl), 3.97–4.01 (m, 1H, CH H_β_-propyl), 6.75–6.84 (m, 2H ArH), 6.87–6.92 (m, 1H, ArH)
**^13^C NMR** (75 MHz, CDCl_3_) δ: 165.65, 136.64, 128.74, 119.50, 119.32, 116.20, 110.73, 110.45, 104.99, 104.66, 104.31, 65.96, 61.70, 53.35, 50.97, 43.83, 41.70, 32.47, 20.02, 13.68, 11.36
**FT-IR** (selected lines, γ_max_, cm^−1^): 1700 (C=O), 1760 (C=O), 3140 (OH)
**ESI-MS** (*m*/*z*): calcd. for C_25_H_33_F_2_N_4_O_3_ [M+H]^+^: 475.5513; found: 475.2527

*4,6-dimethyl-5-butyl-2-[2-hydroxy-3-[4-(o-cyjanophenyl)piperazin-1-yl]propyl]pyrrolo[3,4-c]pyrrole-1,3(2H,5H)-dione* (**3f**).

**3f**: from **2a** and 1-(*o*-cyjanophenyl)piperazine. Yield 79%, m.p. 154–156 °C.
**^1^H NMR** (300 MHz, CDCl_3_) δ: 0.98 (t, 3H, CH_3_-butyl, *J* = 7.5 Hz), 1.26–1.43 (m, 2H CH_2_-butyl,), 1.60–1.63 (m, 2H, CH_2_-butyl,), 2.39 (s, 6H, 4,6-CH_3_), 2.48 (d, 2H, CH_2,_ H_γ_-propyl, *J* = 6.6 Hz), 2.51–2.67 (m, 2H, CH_2_-piperazine), 2.80–2.82 (m, 2H, CH_2_-piperazine), 3.15–3.20 (m, 4H, N(CH_2_)_2_Ar), 3.66 (d, 2H, N_pyrrole_-CH_2_ *J* = 4.5 Hz), 3.75 (t, 2H, CH_2_ H_α_-propyl_,_ *J* = 7.8 Hz), 3.99–4.00 (m, 1H, CH-H_β_-propyl), 6.97–7.02 (m, 2H, ArH), 7.47 (t, 1H, ArH, *J* = 7.5 Hz), 7.53 (d, 1H, ArH, *J* = 7.8 Hz)
**^13^C NMR** (75 MHz, CDCl_3_) δ: 165.64, 155.64, 134.33, 133.74, 128.77, 121.73, 118.67, 118.39, 166.19, 106.05, 66.02, 61.66, 53.29, 51.59, 43.84, 41.65, 32.47, 20.02, 13.69, 11.37
**FT-IR** (selected lines, γ_max_, cm^−1^): 1700 (C=O), 1760 (C=O), 3110 (OH)
**ESI-MS** (*m*/*z*): calcd. for C_26_H_34_N_5_O_3_ [M+H]^+^: 464.5799; found: 464.2637

*4,6-dimethyl-5-(3-chlorophenyl)-2-[2-hydroxy-3-[4-(o-ethoxyphenyl)piperazin-1-yl]propyl]pyrrolo[3,4-c]pyrrole-1,3(2H,5H)-dione* (**3g**).

**3g**: from **2c** and 1-(*o*-ethoxyphenyl)piperazine. Yield 65%, m.p. 129–131 °C.
**^1^H NMR** (300 MHz, CDCl_3_) δ: 1.42 (t, 3H, CH_2_**CH_3_O**), 2.19 (s, 6H, 4,6–CH_3_), 2.48–2.51 (m, 2H, CH_2_, H_γ_-propyl), 2.60–2.65 (m, 2H, CH_2_-piperazine), 2.75–2.83 (m, 2H, CH_2-piperazine_), 3.01–3.15 (m, 4H, N(CH_2_)_2_Ar), 3.68–3.73 (m, 2H, CH_2_), 4.02–4.09 (m, 3H, CH+CH_2_ H_α_-propyl+-H_β_-propyl), 6.83–6.85 (m, 1H, ArH), 6.90–6.93 (m, 3H, ArH), 7.11–7.13 (m, 1H, ArH), 7.20–7.24 (m, 1H, ArH), 7.49–7.52 (m, 2H, ArH)
**^13^C NMR** (75 MHz, CDCl_3_) δ: 165.37, 151.56, 141.32, 137.12, 135.60, 130.89, 129.94, 129.76, 128.22, 126.23, 122.73, 120.99, 118.15, 116.84, 112.53, 65.51, 63.56, 61.80, 53.54, 50.65, 41.80, 14.92, 11.87
**FT-IR** (selected lines, γ_max_, cm^−1^): 1680 (C=O), 1750 (C=O), 3010 (OH)
**ESI-MS** (*m*/*z*): calcd. for C_29_H_34_ClN_4_O_4_ [M+H]^+^: 537.2268; found: 537.2248

*4,6-dimethyl-5-phenyl-2-[2-hydroxy-3-[4-(m-trifluoromethylphenyl)piperazin-1-yl]propyl]pyrrolo[3,4-c]pyrrole-1,3(2H,5H)-dione* (**3h**).

**3h**: from **2b** and 1-(*m*-trifluoromethylphenyl)piperazine. Yield 77%, m.p. 135–138 °C.
**^1^H NMR** (300 MHz, CDCl_3_) δ: 2.17 (s, 6H, 4,6–CH_3_), 2.49 (d, 2H, CH_2_ H_γ_-propyl, *J* = 6.6 Hz), 2.61–2.62 (m, 2H, CH_2_-piperazine), 2.70–2.79 (m, 2H, CH_2_-piperazine), 3.17–3.20 (m, 4H, N(CH_2_)_2_Ar), 3.70–3.73 (m, 2H, CH_2_ H_α_-propyl), 4.00–4.10 (m, 1H, CH H_β_-propyl), 6.85–6.90 (m, 2H, ArH), 6.91–6.93 (m, 2H, ArH), 7.17–7.23 (m, 2H, ArH), 7.26–7.28 (m, 2H, ArH), 7.53–7.55 (m, 3H ArH)
**FT-IR** (selected lines, γ_max_, cm^−1^): 1680 (C=O), 1750 (C=O), 3090 (OH)
**ESI-MS** (*m*/*z*): calcd. for C_28_H_30_F_3_N_4_O_3_ [M+H]^+^: 527.5580; found: 527.8769
*4,6-dimethyl-5-phenyl-2-[2-hydroxy-3-(4-pyrimidynylpiperazin-1-yl)propyl]pyrrolo[3,4-c]pyrrole-1,3(2H,5H)-dione* (**3i**).
**3i**: from **2b** and 1-pyrimidynylpiperazine. Yield 65%, m.p. 165–167 °C.
**^1^H NMR** (300 MHz, CDCl_3_) δ: 2.17 (s, 6H, 4,6–CH_3_), 2.48–2.50 (m, 4H, N(CH_2_)_2_Ar), 2.65–2.70 (m, 2H, CH_2_ H_α_-propyl), 3.70–3.74 (m, 2H, CH_2_ H_β_-propyl), 3.80–3.90 (m, 2H, CH_2_), 4.00–4.09 (m, 1H, CH), 6.47–6.50 (m, 1H, ArH), 7.19–7.22 (m, 2H, ArH), 7.53–7.56 (m, 3H, ArH), 8.29 (d, 2H, ArH, *J* = 4.8 Hz)
**^13^C NMR** (75 MHz, CDCl_3_) δ: 165.61, 161.70, 157.69, 130.06, 129.89, 129.55, 127.84, 116.48, 109.88, 65.85, 61.92, 53.20, 43.79, 41.76, 11.88
**FT-IR** (selected lines, γ_max_, cm^−1^): 1680 (C=O), 1750 (C=O), 3490 (OH)
**ESI-MS** (*m*/*z*): calcd. for C_25_H_29_N_6_O_3_ [M+H]^+^: 461.5362; found: 461.2289

*4,6-dimethyl-5-(3-chlorophenyl)-2-[2-hydroxy-3-(4-pyrimidynylpiperazin-1-yl)propyl]pyrrolo[3,4-c]pyrrole-1,3(2H,5H)-dione* (**3j**).

**3j**: from **2c** and 1-pyrimidynylpiperazine. Yield 72%, m.p. 179–181 °C.
**^1^H NMR** (300 MHz, CDCl_3_) δ: 2.19 (s, 6H, 4,6–CH_3_), 2.47–2.52 (m, 4H, 2xCH_2_Hγpropyl+piperazine), 2.67–2.69 (m, 2H, CH_2_), 3.69–3.73 (m, 2H, N_pyrrole-_CH_2_), 3.80–3.81 (m, 4H, CH_2_ piperazine+CH_2_ H_α_-propyl), 4.00–4.06 (m, 1H, CH H_β_-propyl), 6.48 (t, 2H, ArH, *J* = 4.8 Hz), 7.11–7.13 (m, 1H, ArH), 7.24–7.25 (m, 1H, ArH), 7.50–7.52 (m, 2H, ArH), 8.29 (d, 2H, ArH, *J* = 4.5 Hz)
**^13^C NMR** (75 MHz, CDCl_3_) δ: 165.38, 157.69, 137.09, 135.61, 130.90, 129.96, 129.84, 128.21, 126.21, 116.78, 109.91, 65.75, 61.90, 53.20, 43.77, 41.77, 11.86
**FT-IR** (selected lines, γ_max_, cm^−1^): 1680 (C=O), 1750 (C=O), 3350 (OH)
**ESI-MS** (*m*/*z*): calcd. for C_25_H_28_ClN_6_O_3_ [M+H]^+^: 495.9812; found: 495.1909

*4,6-dimethyl-5-butyl-2-[2-hydroxy-3-(4-pyrimidynyl-1-piperazinyl)propyl]pyrrolo[3,4-c]pyrrole-1,3(2H,5H)-dione* (**3k**).

**3k**: from **2a** and 1-pyrimidynylpiperazine. Yield 65%, m.p. 182–184 °C.
**^1^H NMR** (300 MHz, CDCl_3_) δ: 0.97 (t, 3H, CH_3_-butyl *J* = 7.2 Hz), 1.24–1.42 (m, 2H, CH_2_-butyl), 1.57–1.65 (m, 2H, CH_2_-butyl), 2.38 (s, 6H, 4,6-CH_3_), 2.44–2.49 (m, 4H, N(CH_2_)_2_Ar_,_), 2.64–2.68 (m, 2H, CH_2_-piperazine), 3.64–3.67 (m, 2H, CH_2_ H_α_-propyl), 3.72–3.81 (m, 4H, CH_2_-piperazine + N_pyrrole_-CH_2_), 4.01–4.06 (m, 1H, CH H_β_-propyl), 6.47 (t, 1H, ArH, *J* = 4.8 Hz)), 7.11–7.13 (m, 1H, ArH), 7.50–7.52 (m, 2H, ArH), 7.24–7.26 (m, 1H, ArH), 8.28 (d, 2H, ArH, *J* = 7.8 Hz)
**^13^C NMR** (75 MHz, CDCl_3_) δ: 165.63, 161.67, 157.67, 128.73, 116.20, 109.86, 65.93, 61.91, 53.19, 43.83, 43.76, 41.67, 32.46, 20.01, 13.68, 11.36
**FT-IR** (selected lines, γ_max_, cm^−1^): 1680 (C=O), 1750 (C=O), 3350 (OH)
**ESI-MS** (*m*/*z*): calcd. for C_23_H_33_N_6_O_3_ [M+H]^+^: 441.5465; found: 441.2597

*4,6-dimethyl-5-butyl-2-[2-hydroxy-3-(4-cyclohexylpiperazin-1-yl)propyl]pyrrolo[3,4-c]pyrrole-1,3(2H,5H)-dione* (**3l**).

**3l**: from **2a** and 1-cyclohexylpiperazine. Yield 70%, m.p. 111–113 °C.
**^1^H NMR** (300 MHz, CDCl_3_) δ: 1.19–1.22 (m, 5H, CH_2_), 1.35 (t, 2H, CH_2_, *J* = 6.9 Hz), 1.55–1.64 (m, 2H, CH_2_), 1.75–1.80 (m, 2H, CH_2_), 1.81–1.90 (m, 2H, CH_2_), 2.20 (s, 6H, 4,6–CH_3_), 2.38–2.43 (m, 4H, N(CH_2_)_2_Ar), 2.65–2.70 (m, 5H, CH_2_CH_2_CH), 3.64–3.69 (m, 2H, CH_2_ H_α_-propyl), 3.90–4.05 (m, 1H, CH H_β_-propyl), 4.32–4.34 (m, 1H, CH), 7.16–7.22 (m, 2H, ArH), 7.49–7.55 (m, 3H, ArH)
**^13^C NMR** (75 MHz, CDCl_3_) δ: 165.56, 136.00, 129.96, 129.87, 129.65, 129.51, 127.85, 116.51, 66.60, 65.56, 63.51, 61.68, 53.76, 49.03, 41.77, 28.94, 26.28, 25.85, 13.62, 12.84, 11.88
**FT-IR** (selected lines, γ_max_, cm^−1^): 1680 (C=O), 1760 (C=O), 3310 (OH)
**ESI-MS** (*m*/*z*): calcd. for C_27_H_37_N_4_O_3_ [M+H]^+^: 465.6077 found: 465.2863

*4,6-dimethyl-5-phenyl-2-[2-hydroxy-3-(N-methylsulphonylpiperazin-1-yl)-propyl]pyrrolo[3,4-c]pyrrole-1,3(2H,5H)-dione* (**3m**).

**3m**: from **2b** and N-methylsulphonylpiperazine. Yield 72%, m.p. 136–138 °C.
**^1^H NMR** (300 MHz, CDCl_3_) δ: 2.15 (s, 6H, 4,6–CH_3_), 2.46 (d, 2H, CH_2_ H_γ_-propyl, *J* = 6.6 Hz), 2.54–2.60 (m, 2H, CH_2_-piperazine), 2.68–2.71 (m, 2H, CH_2_-piperazine), 2.76 (s, 3H, CH_3_SO_2_), 3.21–3.24 (m, 4H, N(CH_2_)_2_Ar), 3.67 (d, 2H, CH_2_H_α_-propyl *J* = 5.4 Hz), 3.97–4.00 (m, 1H, CH H_β_-propyl), 7.18–7.21 (m, 2H, ArH), 7.52–7.54 (m, 3H, ArH)
**^13^C NMR** (75 MHz, CDCl_3_) δ: 165.66, 135.85, 130.24, 129.92, 129.61, 127.79, 116.31, 65.55, 61.47, 52.68, 45.92, 41.87, 34.22, 11.89
**FT-IR** (selected lines, γ_max_, cm^−1^): 1320 (SO_2_), 1680 (C=O), 1750 (C=O), 3155(OH)
**ESI-MS** (*m*/*z*): calcd. for C_22_H_29_N_4_O_5_S [M+H]^+^: 461.5544; found: 461.1846

*4,6-dimethyl-5-(3-chlorophenyl)-2-[2-hydroxy-3-(N-methylsulphonylpiperazin-1-yl)-propyl]pyrrolo[3,4-c]pyrrole-1,3(2H,5H)-dione* (**3n**).

**3n**: from **2c** and N-methylsulphonylpiperazine. Yield 79%, m.p. 119–122 °C.
**^1^H NMR** (300 MHz, CDCl_3_) δ: 2.18 (s, 6H, 4,6-CH_3_), 2.47 (d, 2H, CH_2_ H_γ_-propyl, *J* = 6.6 Hz), 2.55–2.59 (m, 2H, CH_2_-piperazine), 2.68–2.73 (m, 2H, CH_2_-piperazine), 2.78 (s, 3H, CH_3_SO_2_), 3.24 (t, 4H, N(CH_2_)_2_Ar), 3.69 (d, 2H, CH_2_, *J* = 5.7 Hz), 3.75 (t, 2H, CH_2_ H_α_-propyl, *J* = 7.8 Hz), 3.95–4.05 (m, 1H, CH H_β_-propyl), 7.11–7.13 (m, 1H, ArH), 7.23–7.24 (m, 1H, ArH), 7.50–7.53 (m, 2H, ArH)
**^13^C NMR** (75 MHz, CDCl_3_) δ: 165.48, 136.98, 135.68, 131.01, 130.08, 128.21, 126.18, 116.65, 66.51, 61.48, 52.68, 41.92, 34.26, 11.88
**FT-IR** (selected lines, γ_max_, cm^−1^): 1320 (SO_2_), 1580 (C=O), 1690 (C=O),3350 (OH)
**ESI-MS** (*m*/*z*): calcd. for C_22_H_28_ClN_4_O_5_S [M+H]^+^: 495.9995; found: 495.1466

*4,6-dimethyl-5-butyl-2-[2-hydroxy-3-(N-methylsulphonylpiperazin-1-yl)-propyl]pyrrolo[3,4-c]pyrrole-1,3(2H,5H)-dione* (**3o**).

**3o**: from **2a** and N-methylsulphonylpiperazine. Yield 75%, m.p. 139–141 °C.
**^1^H NMR** (300 MHz, CDCl_3_) δ: 0.97 (t, 3H, CH_3_-butyl, *J* = 7.2 Hz), 1.37–1.42 (m, 2H, CH_2_-butyl), 1.60–1.65 (m, 2H, CH_2_-butyl), 2.38 (s, 6H, 4,6-CH_3_), 2.44 (d, 2H, CH_2_ H_γ_-propyl, *J* = 6.6 Hz), 2.55–2.59 (m, 2H, CH_2_-piperazine), 2.66–2.70 (m, 2H, CH_2_-piperazine), 2.76 (s, 3H, CH_3_SO_2_), 3.22 (t, 4H, N(CH_2_)_2_Ar), 3.64 (d, 2H, N_pyrrole_-CH_2_ *J* = 5.1 Hz), 3.75 (t, 2H, CH_2_ H_α_-propyl, *J* = 7.5 Hz), 3.90–4.00 (m, 1H, CH H_β_-propyl)
**^13^C NMR** (75 MHz, CDCl_3_) δ: 165.73, 128.97, 116.07, 66.68, 61.43, 52.67, 45.91, 43.87, 41.78, 34.19, 32.45, 20.01, 13.68, 11.37
**FT-IR** (selected lines, γ_max_, cm^−1^): 1320 (SO_2_), 1690 (C=O), 1750 (C=O), 3380 (OH)
**ESI-MS** (*m*/*z*): calcd. for C_20_H_33_N_4_O_5_S [M+H]^+^: 441.5681; found: 441.2178

### 4.2. Materials and Methods of Biological Evaluation

#### 4.2.1. Cell Line and Culture Medium

The NHDF (CC-2511) was purchased from Lonza, Basel. This line was derived from human dermal fibroblasts grown in 5% CO_2_ with 95% humidity at 37 °C. Cells were cultured with 10% FBS and 2 mM L-glutamine, 1.25 μg/mL Amphotericin B, and 100 μg/mL Gentamicin. Twice a week, the morphology and confluence were evaluated under microscopy. The confluence was above 70%, and the NHDF cells were subcultured with TrypLE solution.

#### 4.2.2. Tested Compounds

The new 2-[2-hydroxy-3-(4-substituted-1-piperazinyl)propyl] derivatives were dissolved in DMSO to a concentration of 10 mM and stored at −20 °C. The samples were prepared in four different concentrations of compounds (10 µM, 25 µM, 50 µM, and 100 µM), so the concentration of DMSO for cells was lower than 0.01%.

#### 4.2.3. SRB Assay

Viability evaluation was performed using the Sulforhodamine B (SRB) dye. The SRB assay was carried out 24 h after seeding. The culture plates were fixed with a cold TCA solution with a final concentration of 10% *w*/*v* for 1 h at 4–8 °C. This plate was T_0_ control. T_0_ is cell before treatment tested compounds. The supernatant was removed from the culture. The compounds were added and incubated for 48 h at 5% CO_2_, 95% humidity, and 37 °C. The culture was fixed with a cold TCA solution. The same condition was performed on the T_0_ plate. All plates (tested and T_0_) were washed four times with running water and air-dried at room temperature. A 0.4% SRB solution with 1% *v*/*v* acetic acid was added for 30 min. Then were rinsed five times with a 1% solution of acetic acid and then the plates were air-dried at room temperature. The SRB dye connected to intracellular proteins was dissolved with a 10 mM Trizma base for 30 min with stirring on a shaker. The absorbance was measured at 540 nm with a Varioskan LUX microplate reader (Thermo Scientific, Waltham, MA, USA).

#### 4.2.4. Cyclooxygenase Inhibition Assay

COX peroxidase activity was evaluated by colorimetric evaluation of the occurrence of the oxidized form of N, N, N′, N′-tetramethyl-p-phenylenediamine (TMPD). The reduction of PGG_2_ (prostaglandin G2) to PGH_2_ is due to the oxidation of TMPD. It was prepared for COX standard wells (150 μL Assay Buffer, 10 μL Hemin, 10 μL standard solution), backgrounds wells (120 μL Assay Buffer, 10 μL Hemin, 40 μL inactive sample), and sample wells (120 μL Assay Buffer, 10 μL Hemin, 40 μL COX-1 and COX-2). The tested compounds wells included 110 μL Assay Buffer, 10 μL Hemin, and 40 μL solution of tested compounds, which was dissolved in DMSO in a final concentration of 10 mM. These samples were performed in three replicates for every compound. All samples were weakly mixed and incubated for 5 min at room temperature. Subsequently, 20 μL substrate, i.e., arachidonic acid, was added. The entire plate was shaken for 5 min. The result is a change in color measured at 590 nm with a Varioskan LUX microplate reader (Thermo Scientific, Waltham, MA, USA).

#### 4.2.5. Lipoxygenase Inhibitor Screening Assay

The assay detects and measures the hydroperoxides produced in the lipoxygenation reduction using a purified lipoxygenase (LO). Blank wells were prepared that contained 100 μL Assay buffer. The positive control included 10 μL 1x assay buffer and 90 μL 15-LO, but 100% of the Initial activity samples contained 90 μL 15-LO and 10 μL methanol. Samples with test compounds were prepared in triplicate, containing 90 µL of 15-LO and 10 µL of test compound at the assayed concentration of 10 mM. All samples prepared in this way were incubated for 5 min at room temperature. Subsequently, the substrate, i.e., arachidonic acid, was added. The entire test plate was shaken for 10 min. 100 µL of chromogen was added to the samples. The entire plate was shaken for 5 min. The absorbance was read at 490 nm in a VariuScan microplate reader.

### 4.3. Molecular Modeling (Methodology)

The geometries of designed compounds were optimized at the B3LYP6-31G** level of theory by using the Gaussian 09 program [84,85]. In order to take into account the solvent effect, a PCM (polarizable continuum model) was used [86]. The standard docking protocol in the AutoDock4.2 package was adopted to predict the binding mode of ligands to both cyclooxygenase isoforms (COX-1 and COX-2) and lipoxygenase [87]. As molecular targets, the structures of COX-1 (PDB ID:4O1Z) and COX-2 (PDB ID:4M11) co-crystalized with meloxicam were downloaded from the Protein Data Bank [61]. Additionally, the protein crystal structure of the inhibitor-bound 5-LOX with arachidonic acid was retrieved (PDB ID: 3V99) [88]. The docking procedure was validated by docking meloxicam (COX docking) and arachidonic acid (LOX docking) into the protein crystal and comparing its position with the initial structure. To evaluate the accuracy of docking prediction, the root mean square deviation (RMSD) was estimated on the LigRMSD web server [89]. When the RMSD value was found to be less than 2 Å, the binding mode of compounds was correctly predicted. It is well known that the scoring functions used in the docking algorithms are predicted to give approximate values of the free energy of binding, so the results were validated with biological activity measurements. The procedure for molecular target and inhibitor structure preparation was described in detail in previous studies [57,90,91]. Previous studies have shown that the Lamarckian genetic algorithm is the most efficient and reliable approach to AutoDock4.2, so it was used with a total of 500 runs for each binding site [90]. The obtained results were visualized using a BIOVIA Discovery Studio visualizer and a Chimera package [92,93].

## 5. Conclusions

In conclusion, 15 new derivatives of 4,6-dimethyl-5-aryl/alkyl-2-[2-hydroxy-3-(4-substituted-1-piperazinyl)propyl]pyrrolo[3,4-*c*]pyrrole-1,3(2H,5H)-diones were designed and synthesized. The obtained structures **3a**–**3o** were tested for their inhibitory activity against COX-1, COX-2, and LOX enzymes. The most potent inhibitory activity against the COX-1 enzyme was shown by the compound **3o** (69.56 ± 0.03 μM), while the COX-2 enzyme was shown by the compound **3e** (56.43 ± 0.03 μM). The COX-1 and COX-2 inhibition values for the reference drug Meloxicam are 83.68 ± 0.03 μM and 57.14 ± 0.05 μM, respectively. The four compounds with higher activity against the LOX enzyme than the reference drug Zileuton (13.37 ± 0.03 μM) were compounds **3c** (12.72 ± 0.02 μM), **3e** (13.02 ± 0.02 μM), **3f** (13.15 ± 0.05 μM), and **3h** (13.70 ± 0.07 μM). Taking into account the obtained pharmacological results, compound **3e** deserves special attention as it can inhibit the COX-2 enzyme most potently. Additionally, it shows a COX selectivity ratio higher than that of the reference drug Meloxicam. In addition, it inhibits the LOX enzyme more strongly than the reference Zileuton. Compound **3e** is, therefore, the most promising candidate for further research to obtain new more effective dual COX/LOX inhibitors. To summarize, there is undoubtedly an urgent need for new dual-mode anti-inflammatory agents that prevent the release of both prostaglandins and leukotrienes with a better safety profile. The obtained anti-inflammatory compound 3e with beneficial properties could be a promising agent in the treatment of diseases characterized by chronic inflammation, such as arthritis, neurological, cardiovascular, or autoimmune diseases, or cancer.

## Figures and Tables

**Table 1:** Crystal data and structure refinement for 3e.

Empirical formula	C_25_ H_32_ F_2_ N_4_ O_3_
Formula weight	474.54
Temperature	100(2) K
Wavelength	1.54184 Å
Crystal system	Monoclinic
Space group	P2**_1_**/c
Unit cell dimensions	a = 17.842(4) Å
	b = 16.1601(17) Å
=99.92(3)°	
	c = 8.237(2) Å
Volume	2339.5(8) Å^3^
Z	4
Density (calculated)	1.347 Mg/m^3^
Absorption coefficient	0.832 mm^−1^
F(000)	1008
Crystal size	0.08 × 0.05 × 0.05 mm^3^
Theta range for data collection	2.514 to 78.067°.
Index ranges	−22 ≤ h ≤ 22, −20 ≤ k ≤ 19, −8 ≤ l ≤ 10
Reflections collected	21868
Independent reflections	4834 [R(int) = 0.2042]
Completeness to theta = 67.684°	99.9%
Refinement method	Full-matrix least-squares on F^2^
Data/restraints/parameters	4834/0/310
Goodness-of-fit on F^2^	1.096
Final R indices [I > 2sigma(I)]	R1 = 0.2094, wR2 = 0.4749
R indices (all data)	R1 = 0.2794, wR2 = 0.5065
Extinction coefficient	n/a
Largest diff. peak and hole	0.682 and −0.602 e.Å^−3^

**Table 2:** IC_50_ values were calculated for all tested compounds after 48 h incubation with NHDF cells. COX inhibition was calculated for COX-1 and COX-2 enzymes after incubation for 2 min with the tested compounds at a concentration of 100 µM and COX selectivity ratio; LOX inhibition was calculated; data are presented as mean (SD).

Compound	NHDF Cells	Cyclooxygenase Inhibition Assay		COX Selectivity Ratio	Lipoxygenase Inhibition Assay
	IC_50_ [µM]	IC_50_ [µM]		IC_50(COX-2)_/IC_50(COX-1)_	IC_50_ [µM]
		COX-1	COX-2		LOX
**3a**	207.72 ± 0.11	82.05 ± 0.02	60.16 ± 0.08	0.73	13.94 ± 0.06
**3b**	286.24 ± 0.13	79.10 ± 0.06	59.77 ± 0.05	0.76	13.82 ± 0.05
**3c**	142.29 ± 0.09	78.61 ± 0.08	60.05 ± 0.03	0.76	12.72 ± 0.02
**3d**	86.55 ± 0.05	82.23 ± 0.07	59.70 ± 0.03	0.73	14.32 ± 0.02
**3e**	167.88 ± 0.08	88.71 ± 0.09	56.43 ± 0.03	0.64	13.02 ± 0.02
**3f**	225.96 ± 0.09	89.00 ± 0.06	60.36 ± 0.06	0.68	13.15 ± 0.05
**3g**	433.50 ± 0.14	86.47 ± 0.05	60.55 ± 0.09	0.70	12.80 ± 0.04
**3h**	138.57 ± 0.11	79.01 ± 0.02	59.34 ± 0.04	0.75	13.70 ± 0.07
**3i**	114.78 ± 0.08	82.17 ± 0.11	57.93 ± 0.02	0.71	13.98 ± 0.02
**3j**	243.47 ± 0.05	76.74 ± 0.08	60.28 ± 0.02	0.79	14.25 ± 0.03
**3k**	118.02 ± 0.07	83.60 ± 0.08	60.83 ± 0.01	0.73	14.32 ± 0.02
**3l**	103.05 ± 0.06	75.50 ± 0.11	58.23 ± 0.10	0.77	14.00 ± 0.06
**3m**	563.49 ± 0.16	76.59 ± 0.04	61.00 ± 0.04	0.80	13.85 ± 0.07
**3n**	107.32 ± 0.10	84.93 ± 0.02	60.25 ± 0.07	0.71	13.44 ± 0.02
**3o**	49.47 ± 0.05	69.56 ± 0.03	59.42 ± 0.06	0.85	13.75 ± 0.08
Meloxicam	174.23 ± (0.09)	83.68 ± (0.03)	57.14 ± (0.05)	0.68	-
Zileuton	-		-	-	13.37 ± 0.03

**Table 3:** Drug-likeness parameters according to Lipinski and Veber rules.

Compd.	Lipinski Rules				Veber Rules		
	MW ≤500	LogP ≤5	NHD ^a^ ≤5	NHA ^b^ ≤10	Violations of Rules	NBR ^c^ ≤10	TPSA ^d^ ≤140
**3a**	452.59	4.41	1	4	0	8	70.71
**3b**	517.46	4.40	1	4	1	8	70.71
**3c**	476.54	3.98	1	5	0	6	70.71
**3d**	494.53	3.98	1	6	0	6	70.71
**3e**	474.54	4.41	1	6	0	8	70.71
**3f**	518.01	4.17	0	5	1	6	94.50
**3g**	537.05	4.68	1	5	1	8	79.94
**3h**	526.55	4.34	1	7	1	7	70.71
**3i**	460.53	3.67	1	6	0	6	96.49
**3j**	494.97	3.44	1	6	0	6	96.49
**3k**	440.54	3.60	1	6	0	8	96.49
**3l**	444.61	4.43	1	5	0	8	70.71
**3m**	460.55	2.93	1	7	0	6	113.23
**3n**	511.03	3.95	1	7	1	6	102.23
**3o**	440.56	3.25	1	7	0	8	113.23

^a^ NHD: number of hydrogen bond donors; ^b^ NHA: number of hydrogen bond acceptors; ^c^ NBR: number of rotatable bonds; ^d^ TPSA: total polar surface area.

**Table 4:** Absorption and distribution parameters of compounds **3c**, **3e**, **3g**, **3h**, **3l**, and mxm (meloxicam).

Parameter	Compound					
	3c	3e	3g	3h	3l	mxm
HIA (Human Intestinal Absorption; 1—high probability of being HIA (HIA < 30%); 0—low probability of being HIA))	0.315	0.006	0.008	0.006	0.005	0.004
Caco-2 Permeability; optimal higher than −5.15 log cm/s	−5.32	−5.29	−5.45	−5.46	−5.31	−4.71
MDCK Permeability of high passive permeability: >20 × 10^−6^	2.5 × 10^−5^	9 × 10^−6^	2.5 × 10^−5^	1.3 × 10^−5^	7.8 × 10^−6^	1.8 × 10^−5^
Pgp-inhibitor	+	-	-	+	--	-
Pgp-substrate	-	-	--	--	-	-
PPB (Plasma Protein Binding; Optimal <90%)	84%	81%	96%	92%	82%	99%
VD (Volume Distribution; 0.04–20 L/kg)	1.3	1.9	1.0	2.4	0.9	0.4
BBB Penetration (Blood-Brain Barrier Penetration; 1—high probability of penetration; 0—low probability of penetration)	0.6	0.4	0.8	0.4	0.5	0.1

**Table 5:** Metabolism and excretion parameters of compounds **3c**, **3e**, **3g**, **3h**, **3l**, and mxm (meloxicam).

Parameter	Compound					
	3c	3e	3g	3h	3l	mxm
CYP1A2 inhibitor	No	No	No	No	No	No
CYP2C19 inhibitor	Yes	Yes	Yes	Yes	No	No
CYP2C9 inhibitor	Yes	Yes	Yes	Yes	No	Yes
CYP2D6 inhibitor	No	Yes	Yes	No	Yes	No
CYP3A4 inhibitor	Yes	Yes	Yes	Yes	No	No

## Data Availability

Not applicable.

## Supplementary Materials

The following supporting information can be downloaded at: https://www.mdpi.com/article/10.3390/ph16060804/s1, Figure S1: The intermolecular interactions of 3b in the active centre of a) COX-1 b) COX-2; Figure S2: The intermolecular interactions of 3d in the active centre of a) COX-1 b) COX-2; Figure S3: The intermolecular interactions of 3h in the active centre of a) COX-1 b) COX-2; Figure S4: The intermolecular interactions of 3i in the active centre of a) COX-1 b) COX-2; Figure S5: The intermolecular interactions of 3l in the active centre of a) COX-1 b) COX-2; Figure S6: The intermolecular interactions of 3o in the active centre of a) COX-1 b) COX-2; Figures S7–S36: ^1^H NMR and ^13^C NMR ^1^H NMR of compounds 3a–3o; Figures S37–S38: ^1^H NMR and ^13^C NMR of compound 2c; Figures S39–S53: ESI-MS; Figures S54–S68: FT-IR of compounds 3a–3o.
