# Supplementary material for: Design, Synthesis, Biological Evaluation, and Molecular Docking Study of 4,6-Dimethyl-5-aryl/alkyl-2-[2-hydroxy-3-(4-substituted-1-piperazinyl)propyl]pyrrolo[3,4-c]pyrrole-1,3(2H,5H)-diones as Anti-Inflammatory Agents with Dual Inhibition of COX and LOX
Source: Pharmaceuticals (Basel). 2023 May 29;16(6):804. doi: 10.3390/ph16060804 (PMC10303166; doi:10.3390/ph16060804)
Supplement: Supplementary file 1 [file pharmaceuticals-16-00804-s001.zip › pharmaceuticals-2390358-supplementary.pdf]

Figure S1. The intermolecular interactions of 3b in the active centre of a) COX-1 b) COX-2 a)

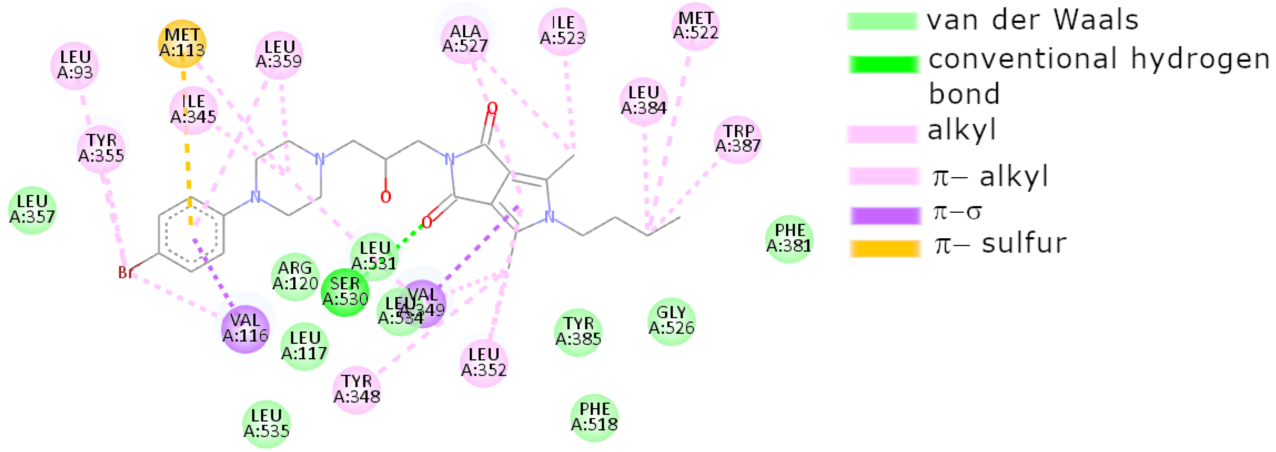

b)

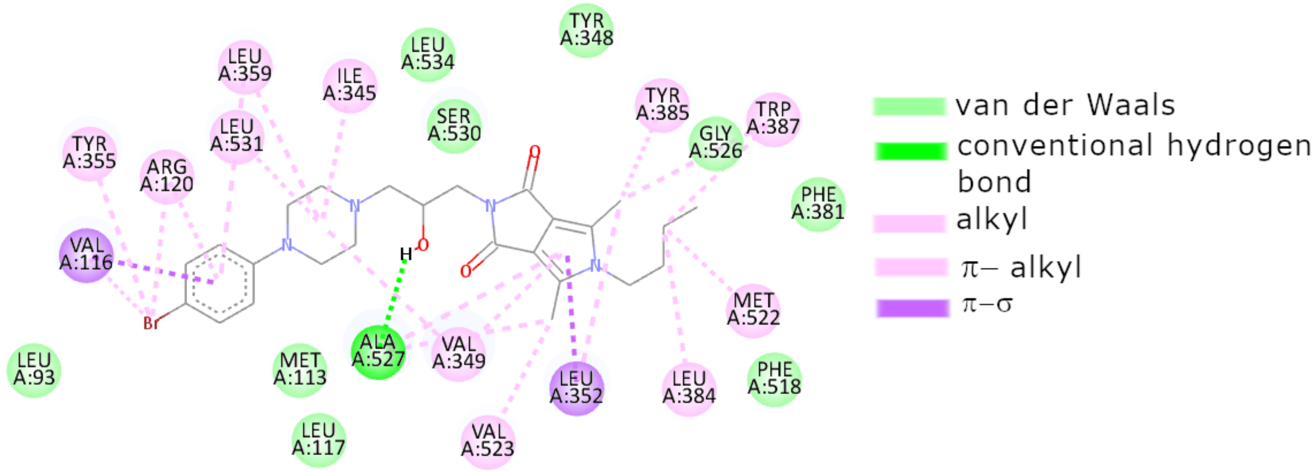

Figure S2. The intermolecular interactions of 3d in the active centre of a) COX-1 b) COX-2 a)

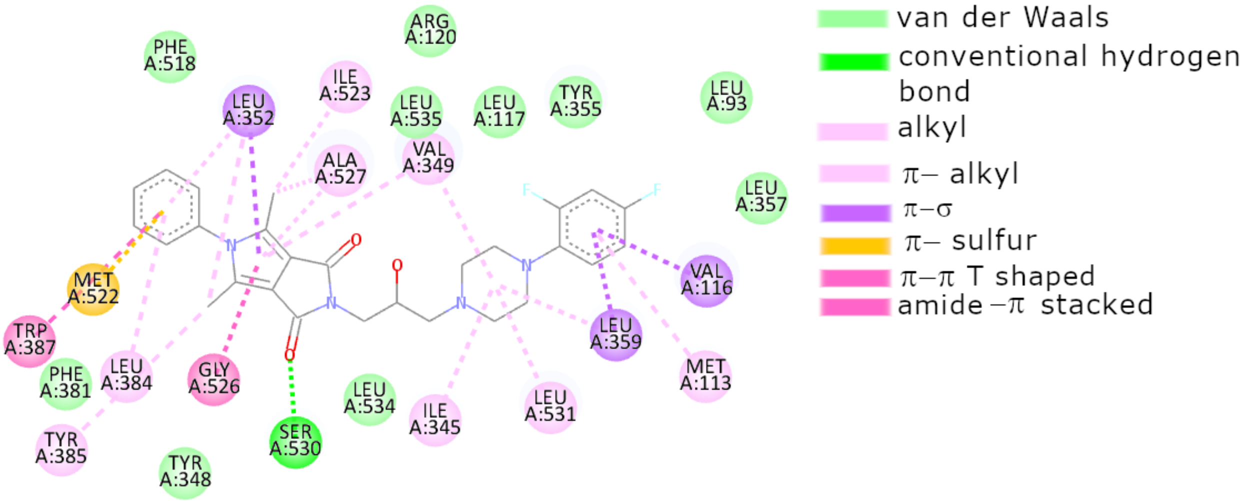

b)

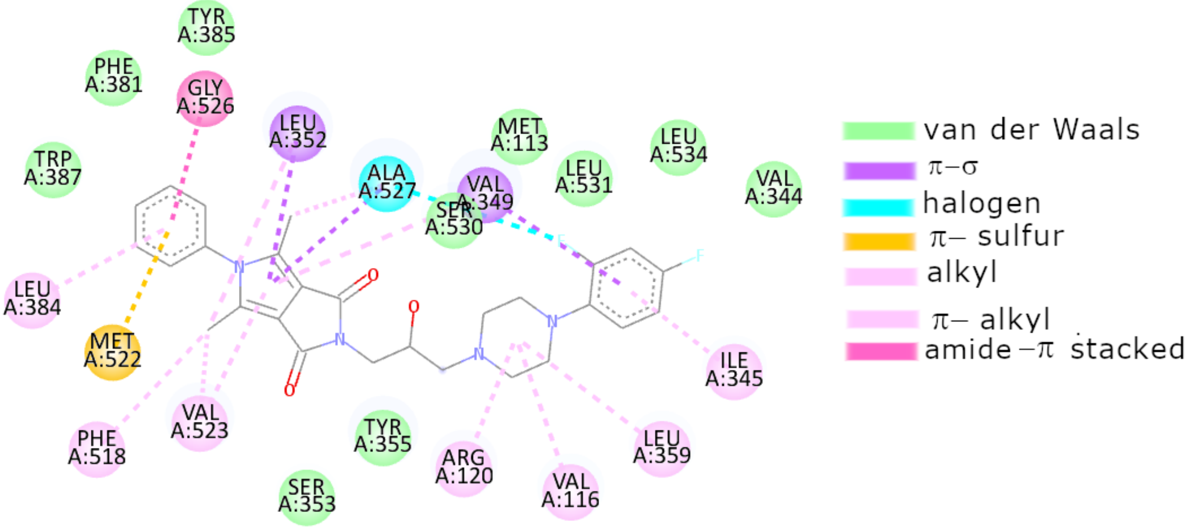

Figure S3. The intermolecular interactions of 3h in the active centre of a) COX-1 b) COX-2 a)

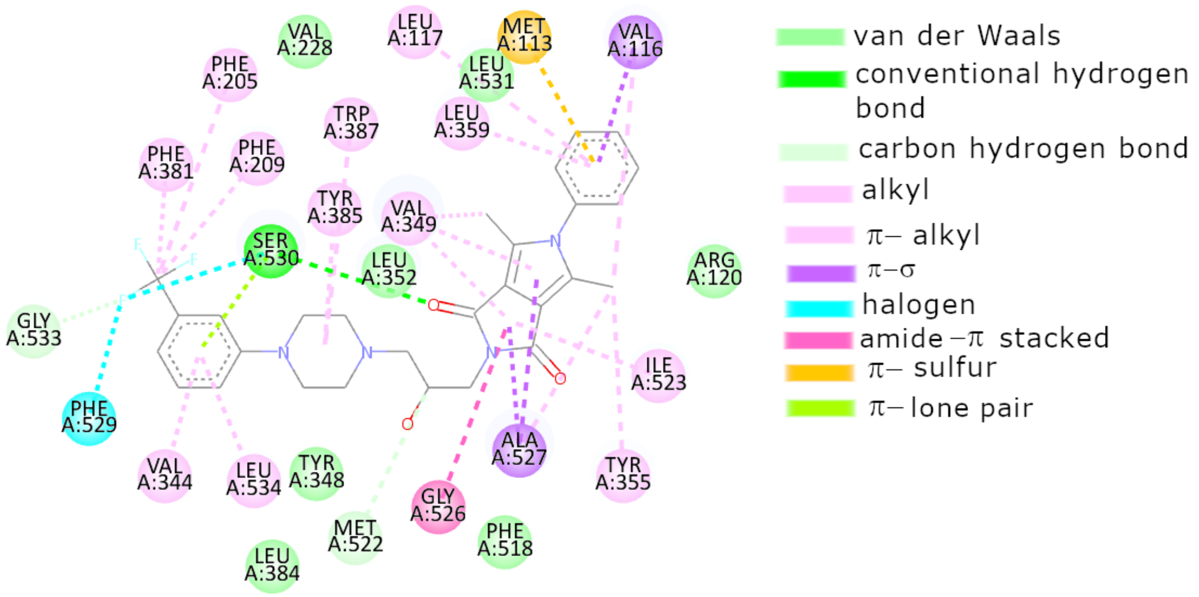

b)

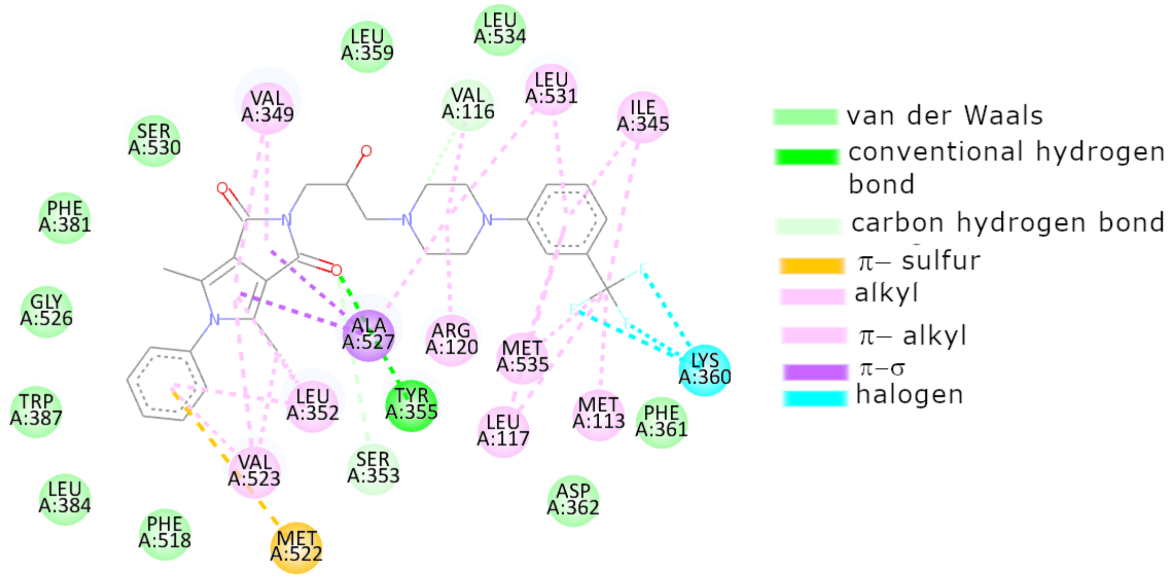

Figure S4. The intermolecular interactions of 3i in the active centre of a) COX-1 b) COX-2 a)

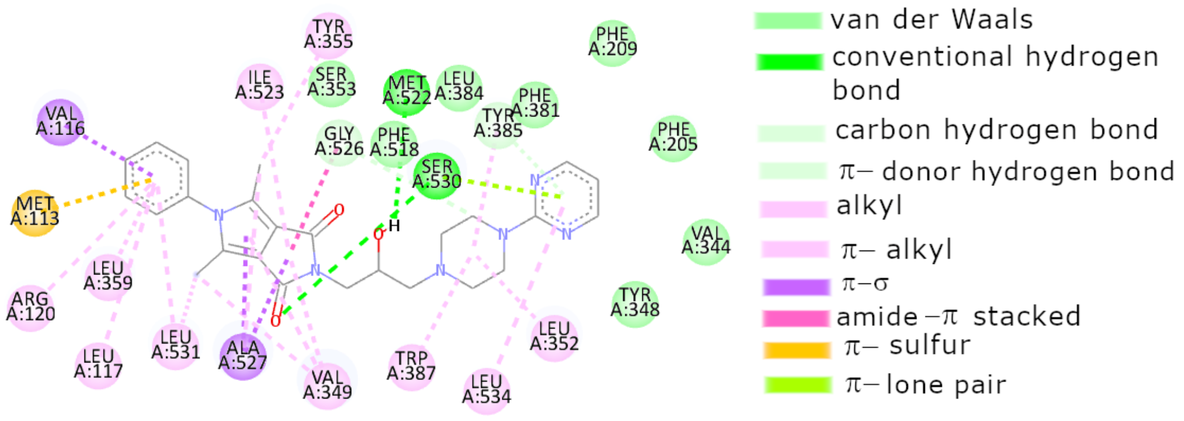

b)

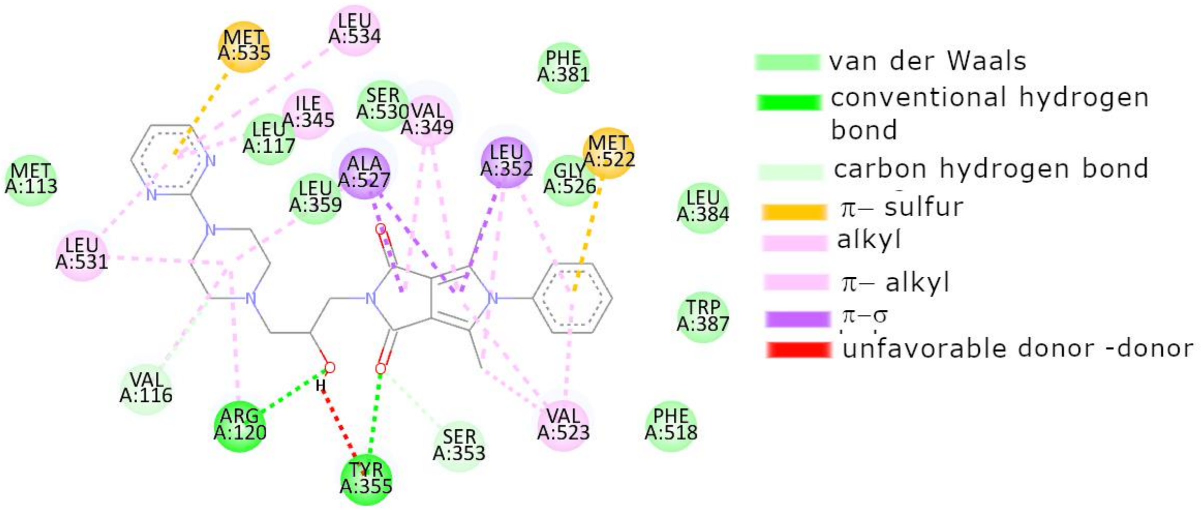

Figure S5. The intermolecular interactions of 3l in the active centre of a) COX-1 b) COX-2 a)

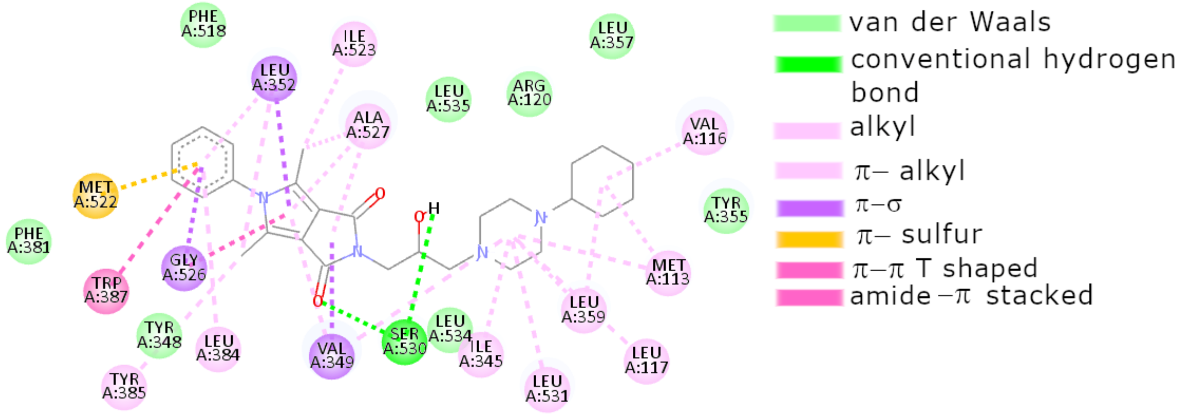

b)

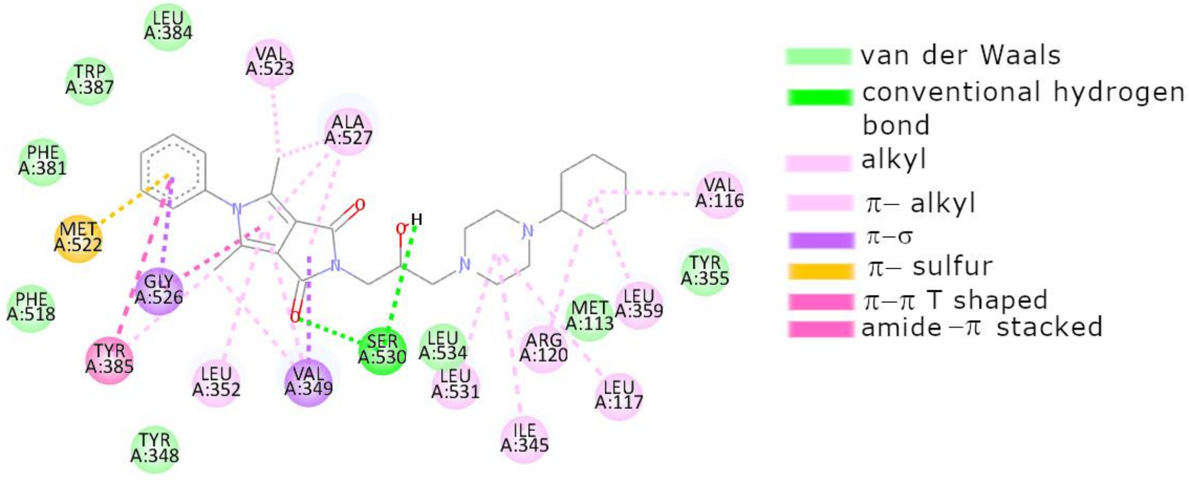

Figure S6. The intermolecular interactions of 3o in the active centre of a) COX-1 b) COX-2 a)

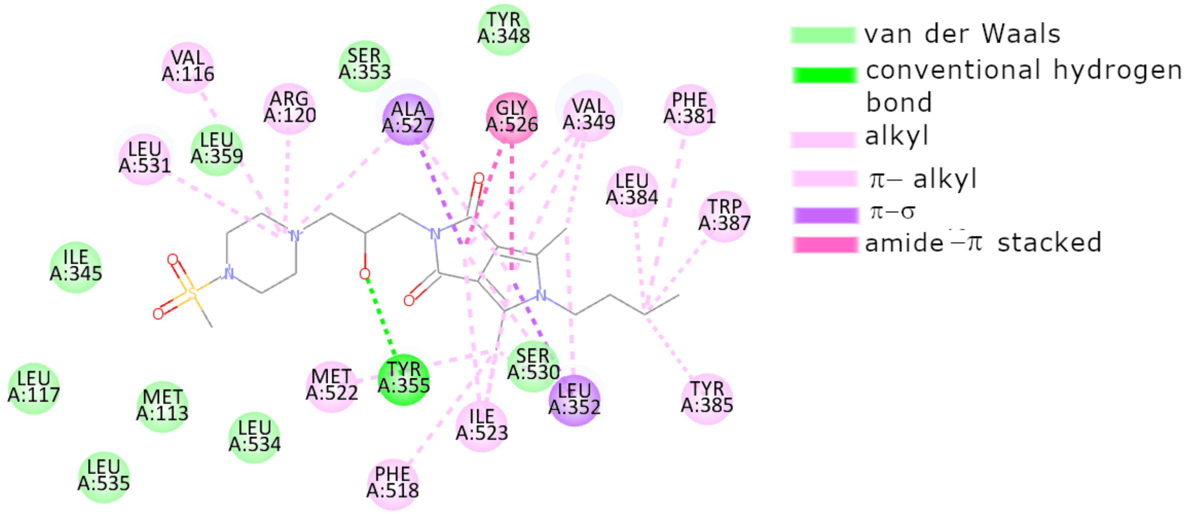

b)

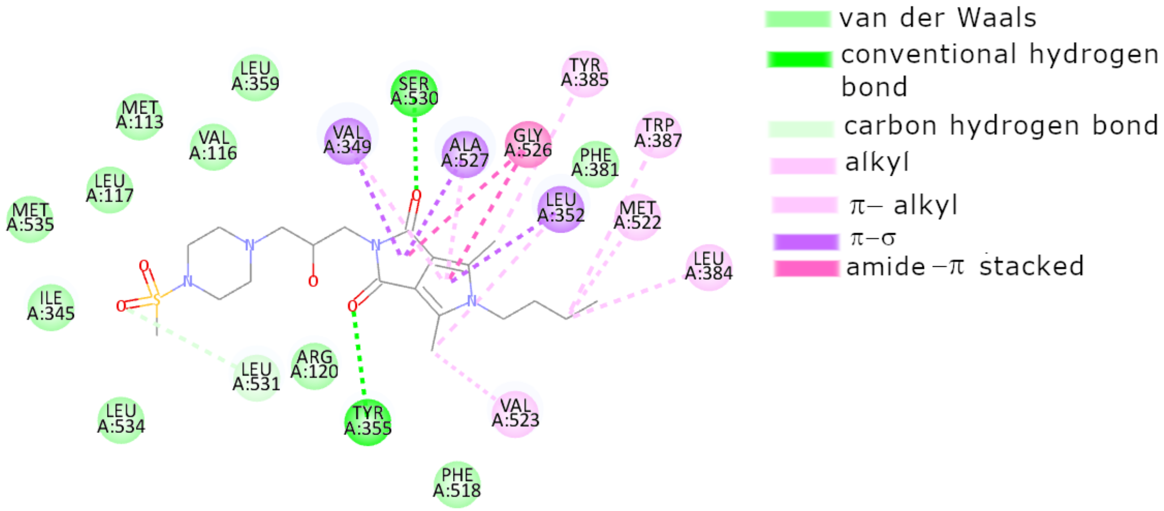

3a C

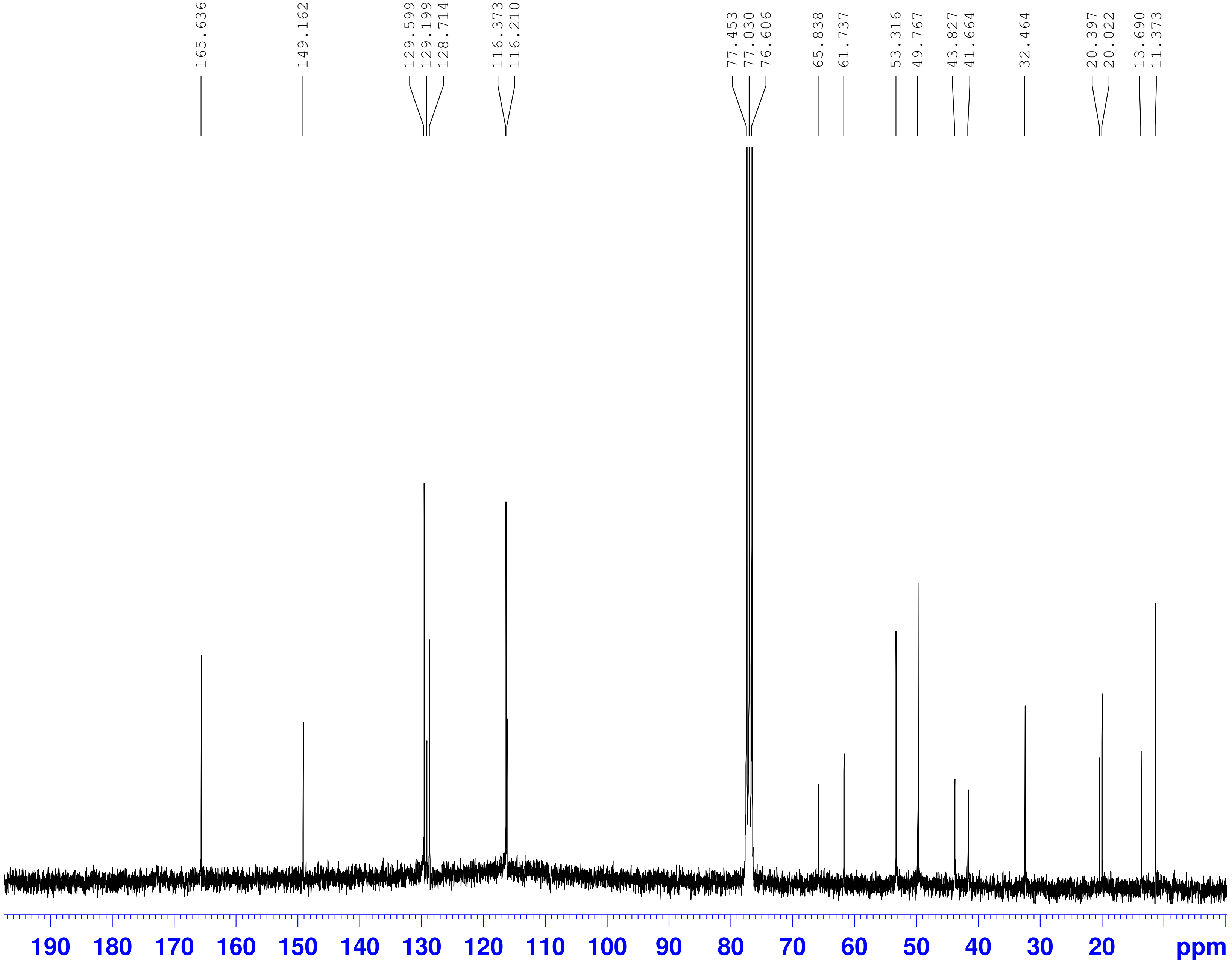

BRUKER

Current Data Parameters
NAME Redzicka 1018
EXPNO 2
PROCNO 1

F2 - Acquisition Parameters
Date_ 20210721
Time 15.12
INSTRUM spect
PROBHD 5 mm BBI 1H/D-
PULPROG zgpg30
TD 65536
SOLVENT CDCl3
NS 4096
DS 2
SWH 17985.611 Hz
FIDRES 0.274439 Hz
AQ 1.8219508 sec
RG 18390.4
DW 27.800 usec
DE 20.00 usec
TE 299.1 K
D1 2.00000000 sec
d11 0.03000000 sec
DELTA 1.89999998 sec
TD0 1

======== CHANNEL f1 ========
NUC1 13C
P1 11.90 usec
PL1 -6.00 dB
SFO1 75.4803248 MHz

======== CHANNEL f2 ========
CPDPRG2 waltz16
NUC2 1H
PCPD2 100.00 usec
PL2 2.00 dB
PL12 21.66 dB
PL13 23.00 dB
SFO2 300.1512006 MHz

F2 - Processing parameters
SI 32768
SF 75.4727782 MHz
WDW EM
SSB 0
LB 1.00 Hz
GB 0
PC 2.00

3a H

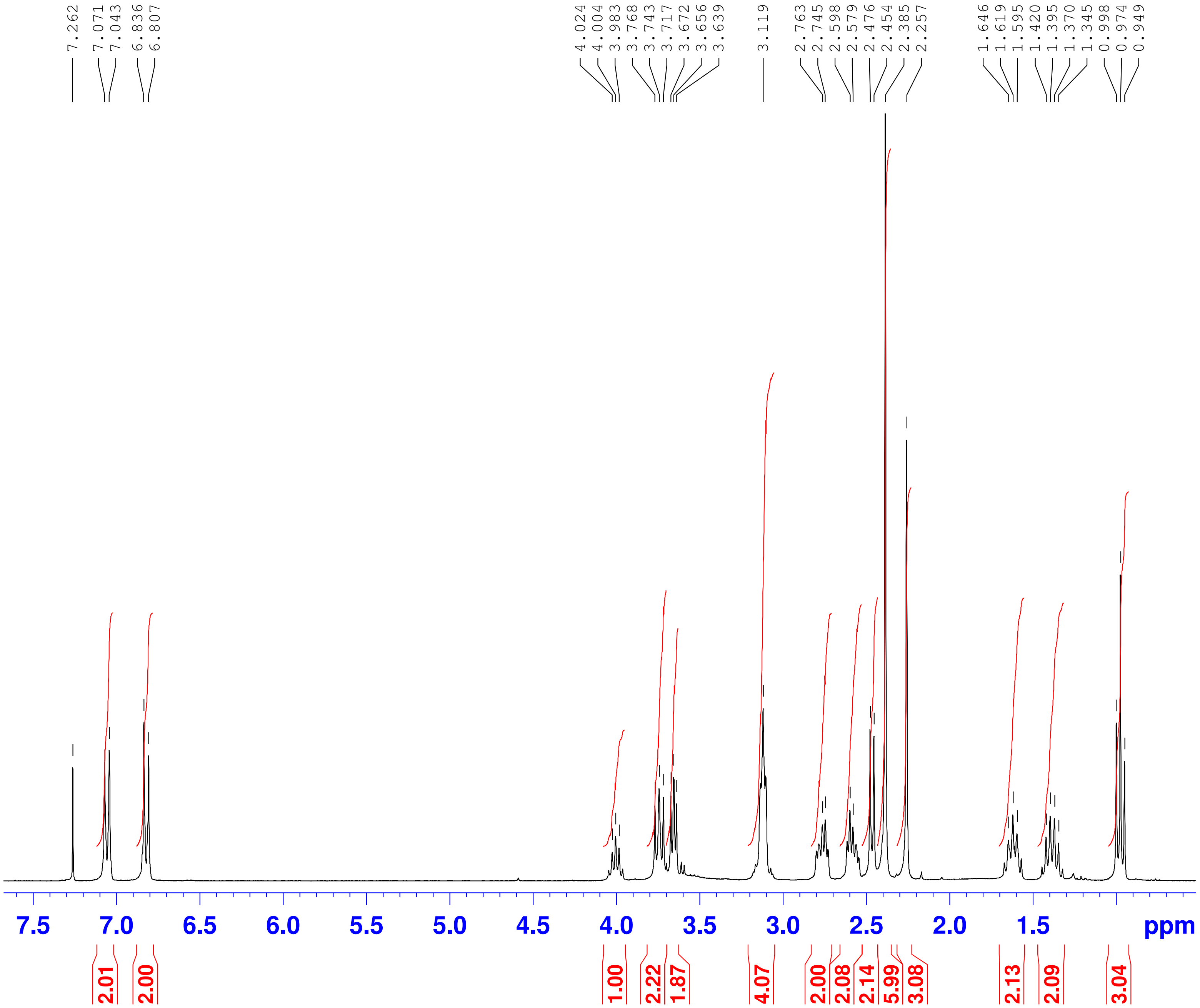

BRUKER

Current Data Parameters
NAME Redzicka 1018
EXPNO 1
PROCNO 1

F2 - Acquisition Parameters
Date_ 20210721
Time 10.44
INSTRUM spect
PROBHD 5 mm BBI 1H/D-
PULPROG zg30
TD 65536
SOLVENT CDCl3
NS 16
DS 0
SWH 6172.839 Hz
FIDRES 0.094190 Hz
AQ 5.3084660 sec
RG 228.1
DW 81.000 usec
DE 8.00 usec
TE 298.1 K
D1 1.00000000 sec
TD0 1

======== CHANNEL f1 ========
NUC1 1H
P1 10.40 usec
PL1 2.00 dB
SFO1 300.1518535 MHz

F2 - Processing parameters
SI 32768
SF 300.1500045 MHz
WDW EM
SSB 0
LB 0.30 Hz
GB 0
PC 20.00

3b C

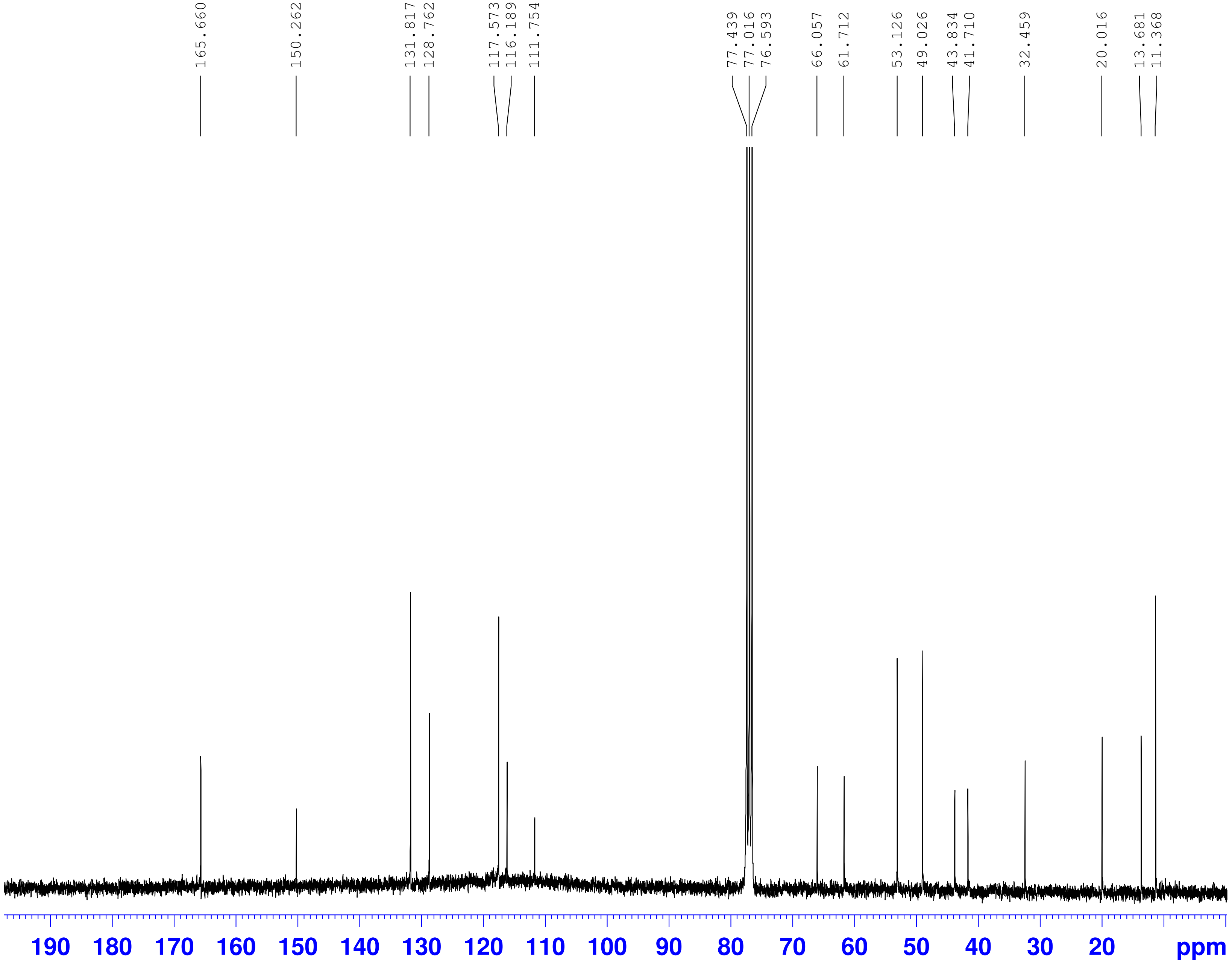

Current Data Parameters
NAME Redzicka 1016
EXPNO 2
PROCNO 1

F2 - Acquisition Parameters
Date_ 20210720
Time 15.35
INSTRUM spect
PROBHD 5 mm BBI 1H/D-
PULPROG zgpg30
TD 65536
SOLVENT CDCl3
NS 5120
DS 2
SWH 17985.611 Hz
FIDRES 0.274439 Hz
AQ 1.8219508 sec
RG 16384
DW 27.800 usec
DE 20.00 usec
TE 299.3 K
D1 2.00000000 sec
d11 0.03000000 sec
DELTA 1.89999998 sec
TD0 1

======== CHANNEL f1 ========
NUC1 13C
P1 11.90 usec
PL1 -6.00 dB
SFO1 75.4803248 MHz

======== CHANNEL f2 ========
CPDPRG2 waltz16
NUC2 1H
PCPD2 100.00 usec
PL2 2.00 dB
PL12 21.66 dB
PL13 23.00 dB
SFO2 300.1512006 MHz

F2 - Processing parameters
SI 32768
SF 75.4727782 MHz
WDW EM
SSB 0
LB 1.00 Hz
GB 0
PC 2.00

3b H

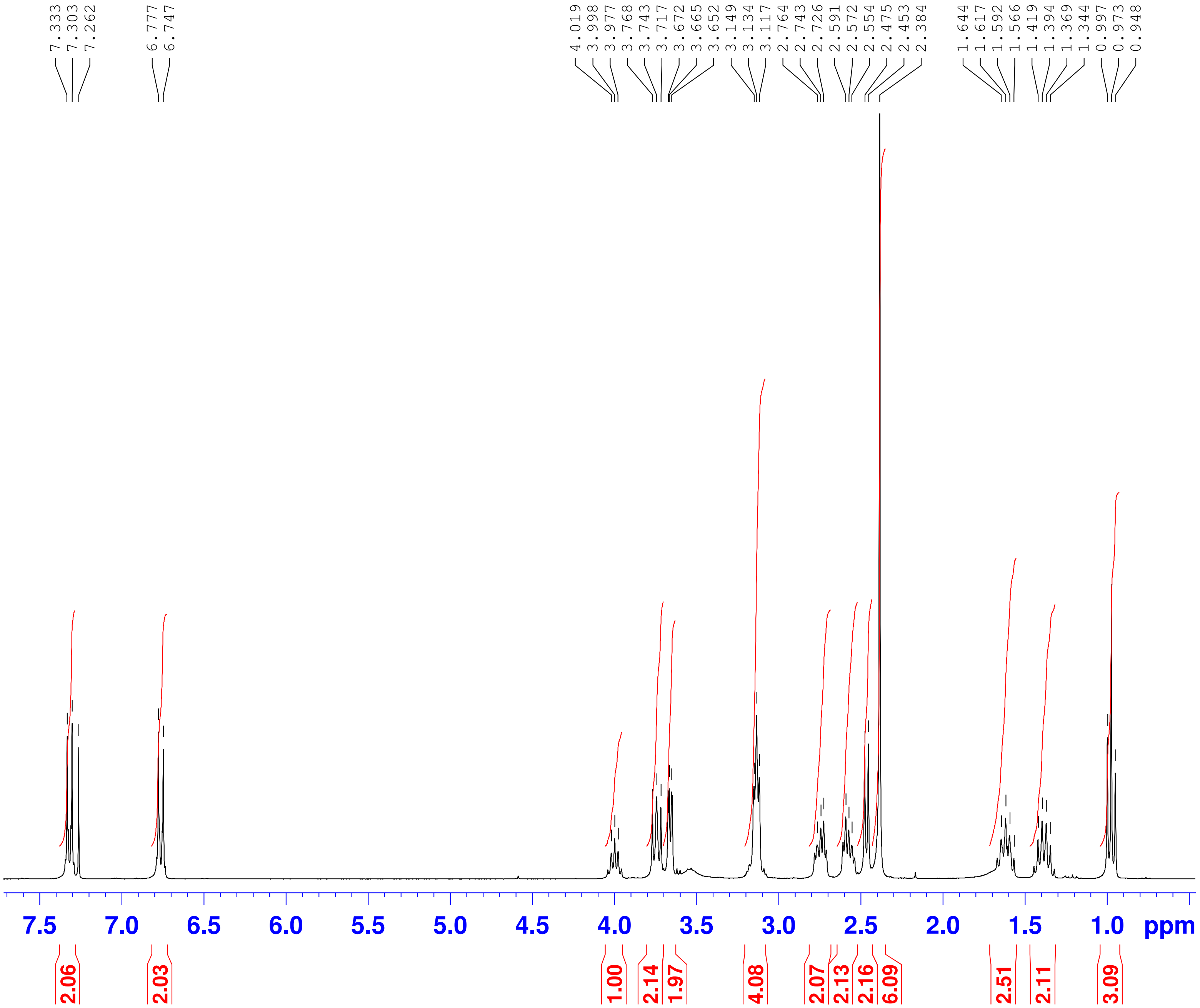

Current Data Parameters

NAME	Redzicka 1016
EXPNO	1
PROCNO	1

F2 - Acquisition Parameters

Date_	20210720
Time	10.00
INSTRUM	spect
PROBHD	5 mm BBI 1H/D-
PULPROG	zg30
TD	65536
SOLVENT	CDCl3
NS	16
DS	0
SWH	6172.839 Hz
FIDRES	0.094190 Hz
AQ	5.3084660 sec
RG	203.2
DW	81.000 usec
DE	8.00 usec
TE	298.1 K
D1	1.00000000 sec
TD0	1

======== CHANNEL f1 ========

NUC1	1H
P1	10.40 usec
PL1	2.00 dB
SFO1	300.1518535 MHz

F2 - Processing parameters

SI	32768
SF	300.1500045 MHz
WDW	EM
SSB	0
LB	0.30 Hz
GB	0
PC	20.00

3c C

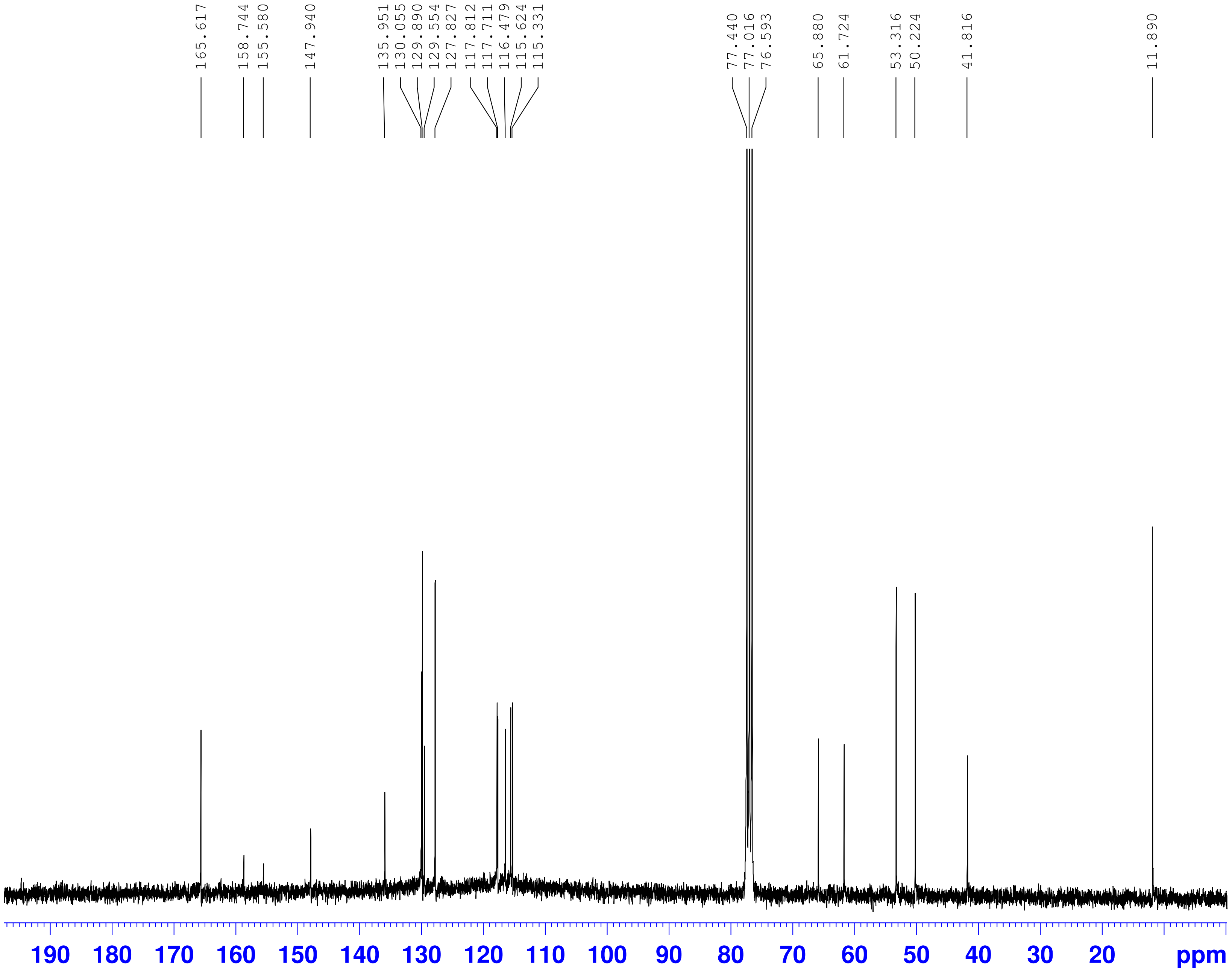

Current Data Parameters
NAME Redzicka 1015
EXPNO 2
PROCNO 1

F2 - Acquisition Parameters
Date_ 20210719
Time 22.16
INSTRUM spect
PROBHD 5 mm BBI 1H/D-
PULPROG zgpg30
TD 65536
SOLVENT CDCl3
NS 5120
DS 2
SWH 17985.611 Hz
FIDRES 0.274439 Hz
AQ 1.8219508 sec
RG 23170.5
DW 27.800 usec
DE 20.00 usec
TE 299.4 K
D1 2.00000000 sec
d11 0.03000000 sec
DELTA 1.89999998 sec
TD0 1

======== CHANNEL f1 ========
NUC1 13C
P1 11.90 usec
PL1 -6.00 dB
SFO1 75.4803248 MHz

======== CHANNEL f2 ========
CPDPRG2 waltz16
NUC2 1H
PCPD2 100.00 usec
PL2 2.00 dB
PL12 21.66 dB
PL13 23.00 dB
SFO2 300.1512006 MHz

F2 - Processing parameters
SI 32768
SF 75.4727782 MHz
WDW EM
SSB 0
LB 1.00 Hz
GB 0
PC 2.00

3c H

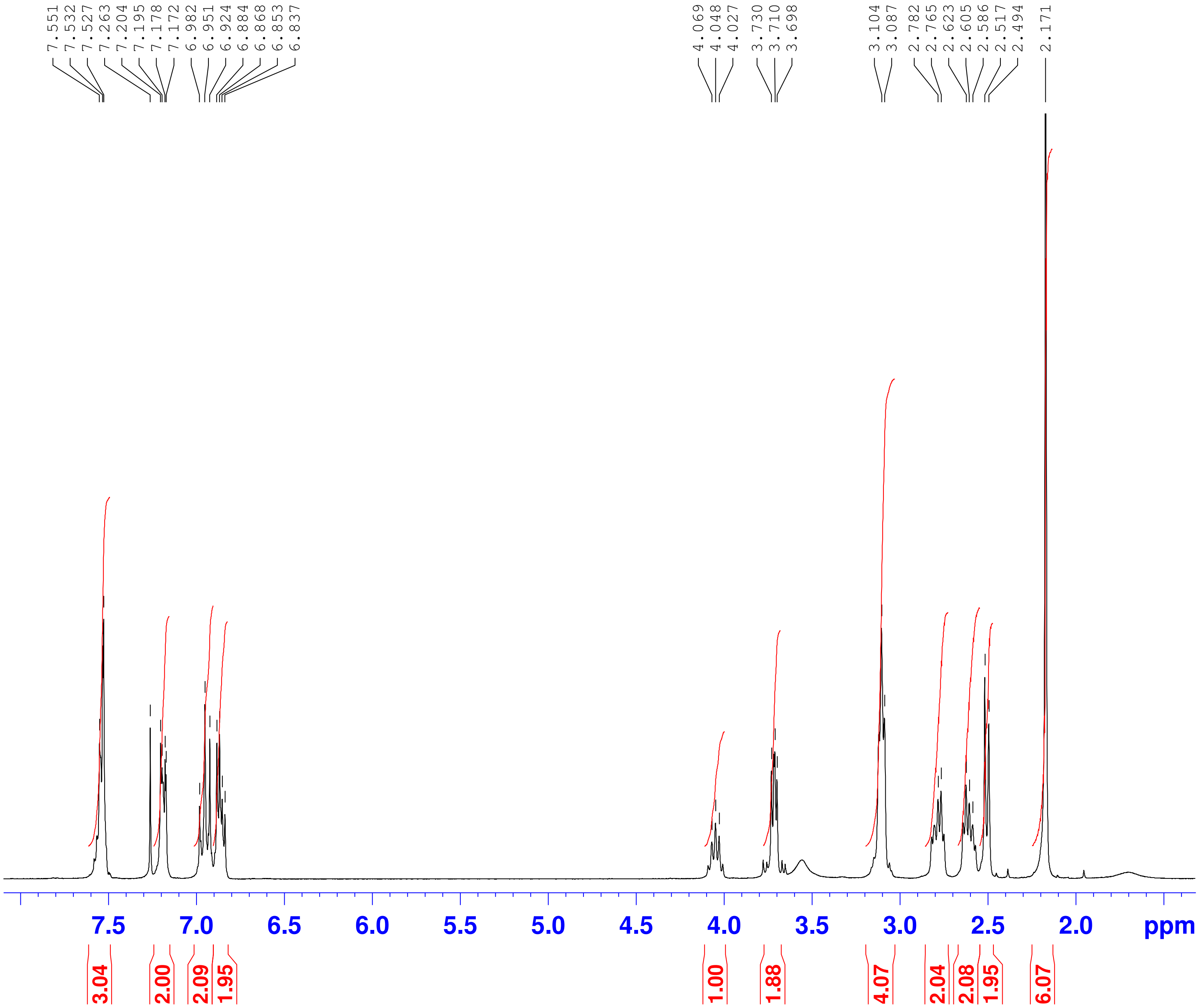

Current Data Parameters	
NAME	Redzicka 1015
EXPNO	1
PROCNO	1
F2 - Acquisition Parameters	
Date_	20210719
Time	16.42
INSTRUM	spect
PROBHD	5 mm BBI 1H/D-
PULPROG	zg30
TD	65536
SOLVENT	CDCl3
NS	16
DS	0
SWH	6172.839 Hz
FIDRES	0.094190 Hz
AQ	5.3084660 sec
RG	228.1
DW	81.000 usec
DE	8.00 usec
TE	298.1 K
D1	1.00000000 sec
TD0	1
======== CHANNEL f1 ========	
NUC1	1H
P1	10.40 usec
PL1	2.00 dB
SFO1	300.1518535 MHz
F2 - Processing parameters	
SI	32768
SF	300.1500045 MHz
WDW	EM
SSB	0
LB	0.30 Hz
GB	0
PC	20.00

3d C

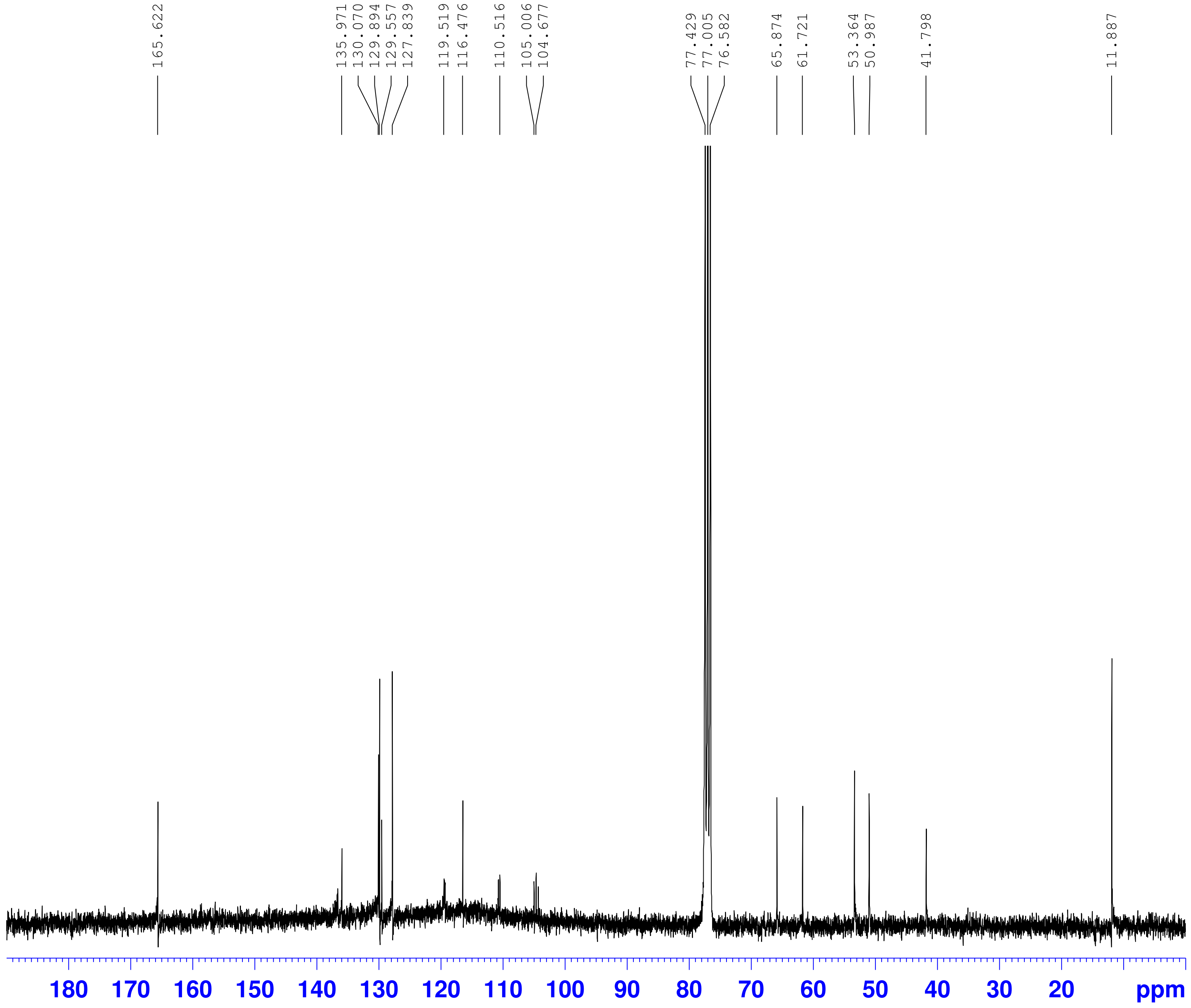

Current Data Parameters
NAME Redzicka 1114
EXPNO 2
PROCNO 1

F2 - Acquisition Parameters
Date_ 20210826
Time 3.08
INSTRUM spect
PROBHD 5 mm BBI 1H/D-
PULPROG zgpg30
TD 65536
SOLVENT CDCl3
NS 10240
DS 2
SWH 17985.611 Hz
FIDRES 0.274439 Hz
AQ 1.8219508 sec
RG 11585.2
DW 27.800 usec
DE 20.00 usec
TE 299.6 K
D1 2.00000000 sec
d11 0.03000000 sec
DELTA 1.89999998 sec
TD0 1

======== CHANNEL f1 ========
NUC1 13C
P1 11.00 usec
PL1 -6.00 dB
SFO1 75.4803248 MHz

======== CHANNEL f2 ========
CPDPRG2 waltz16
NUC2 1H
PCPD2 100.00 usec
PL2 2.00 dB
PL12 21.45 dB
PL13 23.00 dB
SFO2 300.1512006 MHz

F2 - Processing parameters
SI 32768
SF 75.4727780 MHz
WDW EM
SSB 0
LB 1.00 Hz
GB 0
PC 2.00

3d H

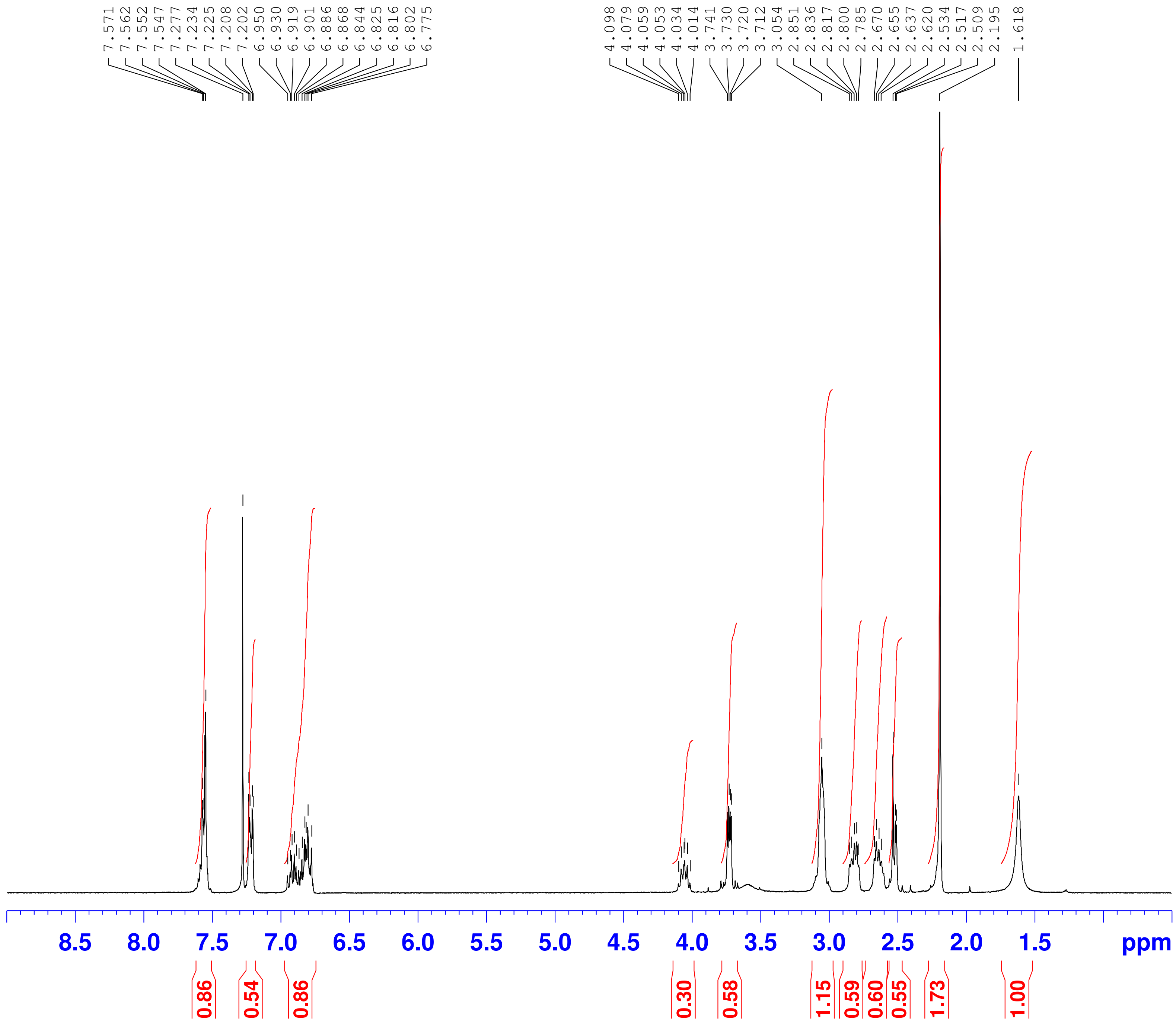

Current Data Parameters
NAME Redzicka 1114
EXPNO 1
PROCNO 1

F2 - Acquisition Parameters
Date_ 20210825
Time 16.04
INSTRUM spect
PROBHD 5 mm BBI 1H/D-
PULPROG zg30
TD 65536
SOLVENT CDCl3
NS 16
DS 0
SWH 6172.839 Hz
FIDRES 0.094190 Hz
AQ 5.3084660 sec
RG 456.1
DW 81.000 usec
DE 8.00 usec
TE 298.4 K
D1 1.00000000 sec
TD0 1

======== CHANNEL f1 ========
NUC1 1H
P1 11.00 usec
PL1 2.50 dB
SFO1 300.1518535 MHz

F2 - Processing parameters
SI 32768
SF 300.1500000 MHz
WDW EM
SSB 0
LB 0.30 Hz
GB 0
PC 1.00

3e C

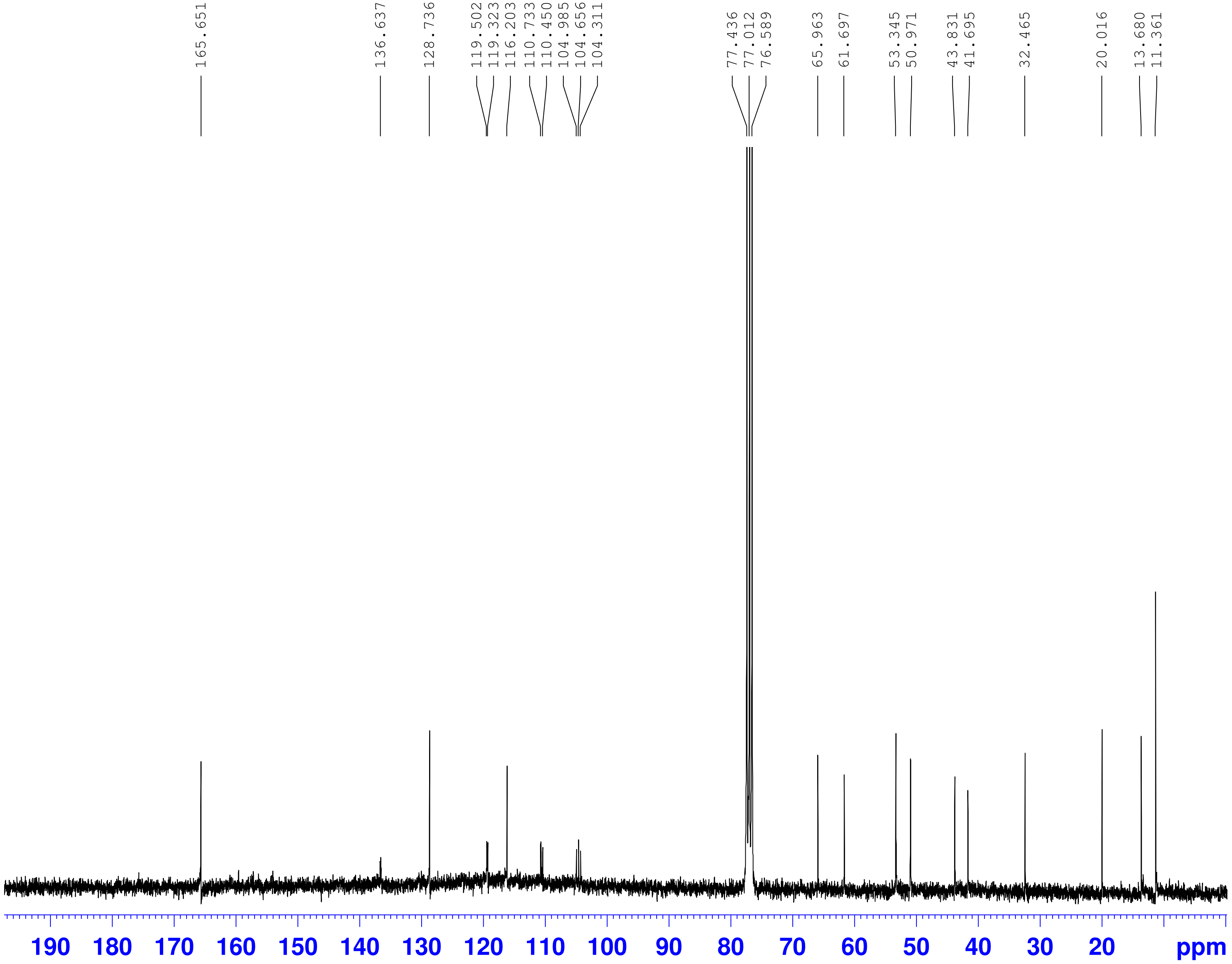

BRUKER

Current Data Parameters
NAME Redzicka 1020
EXPNO 2
PROCNO 1

F2 - Acquisition Parameters
Date_ 20210722
Time 15.43
INSTRUM spect
PROBHD 5 mm BBI 1H/D-
PULPROG zgpg30
TD 65536
SOLVENT CDCl3
NS 5120
DS 2
SWH 17985.611 Hz
FIDRES 0.274439 Hz
AQ 1.8219508 sec
RG 18390.4
DW 27.800 usec
DE 20.00 usec
TE 299.2 K
D1 2.00000000 sec
d11 0.03000000 sec
DELTA 1.89999998 sec
TD0 1

======== CHANNEL f1 ========
NUC1 13C
P1 11.90 usec
PL1 -6.00 dB
SFO1 75.4803248 MHz

======== CHANNEL f2 ========
CPDPRG2 waltz16
NUC2 1H
PCPD2 100.00 usec
PL2 2.00 dB
PL12 21.66 dB
PL13 23.00 dB
SFO2 300.1512006 MHz

F2 - Processing parameters
SI 32768
SF 75.4727782 MHz
WDW EM
SSB 0
LB 1.00 Hz
GB 0
PC 2.00

3e H

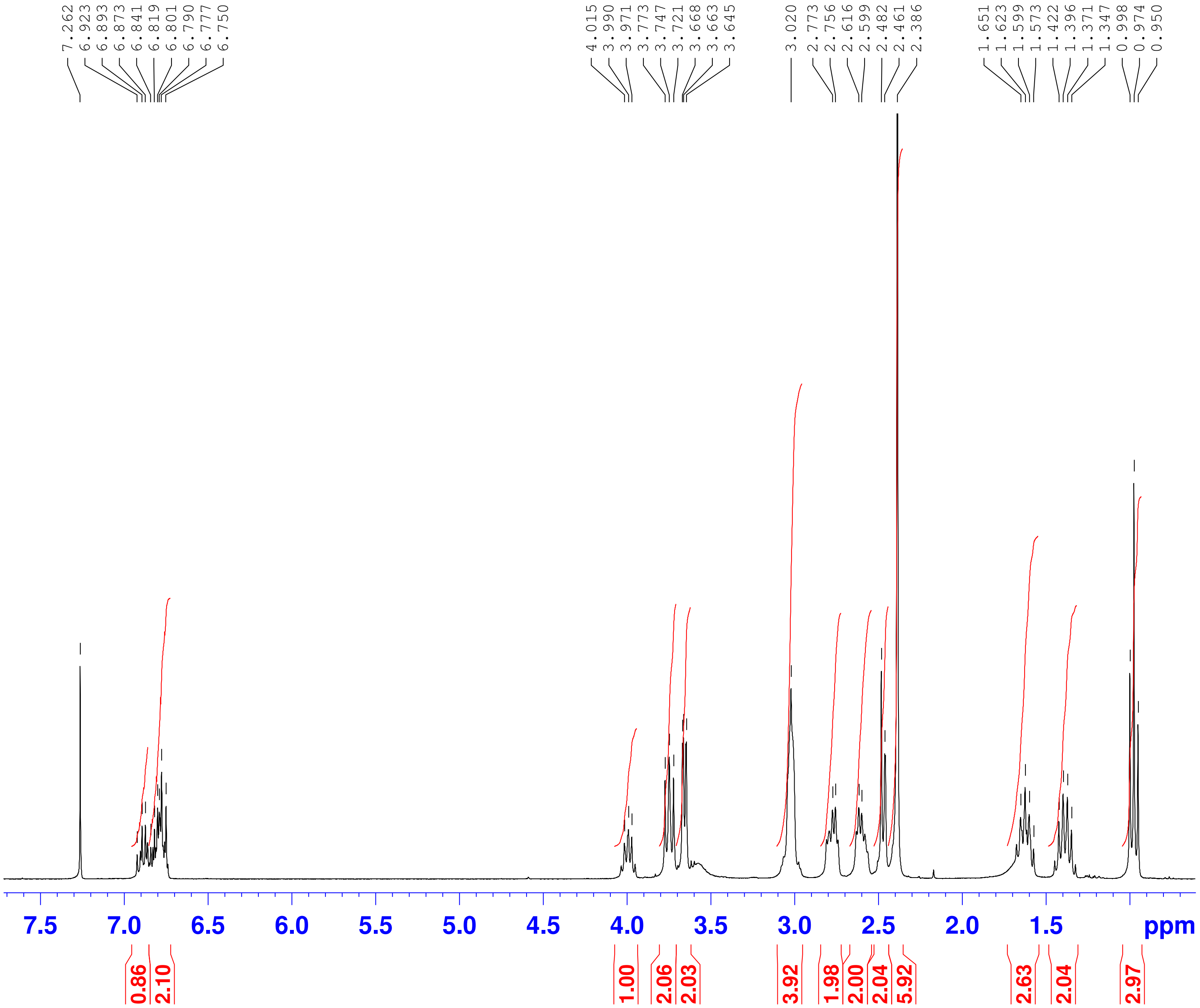

Current Data Parameters
NAME Redzicka 1020
EXPNO 1
PROCNO 1

F2 - Acquisition Parameters
Date_ 20210722
Time 10.06
INSTRUM spect
PROBHD 5 mm BBI 1H/D-
PULPROG zg30
TD 65536
SOLVENT CDCl3
NS 16
DS 0
SWH 6172.839 Hz
FIDRES 0.094190 Hz
AQ 5.3084660 sec
RG 228.1
DW 81.000 usec
DE 8.00 usec
TE 298.1 K
D1 1.00000000 sec
TD0 1

======== CHANNEL f1 ========
NUC1 1H
P1 10.40 usec
PL1 2.00 dB
SFO1 300.1518535 MHz

F2 - Processing parameters
SI 32768
SF 300.1500045 MHz
WDW EM
SSB 0
LB 0.30 Hz
GB 0
PC 20.00

3f C

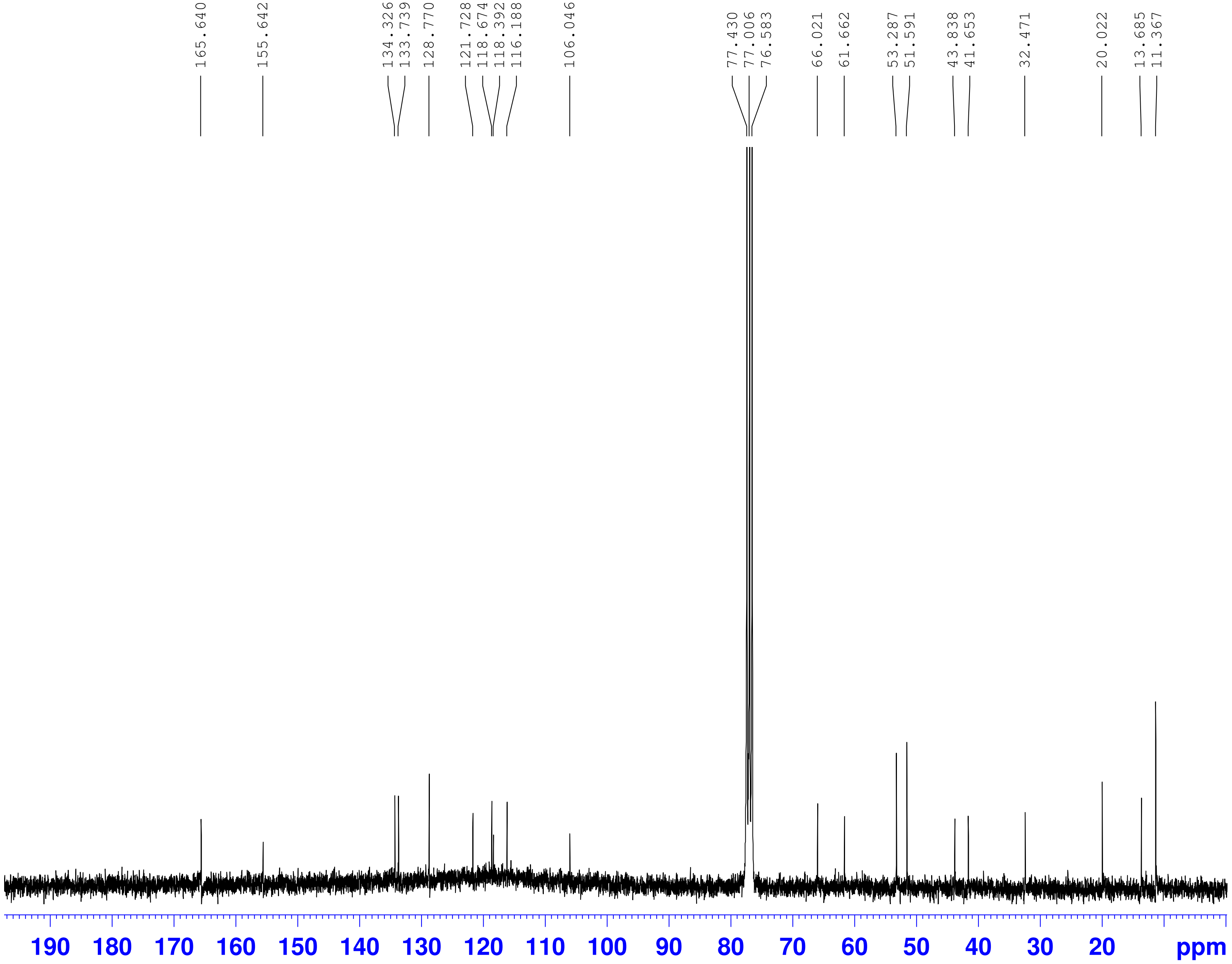


```
Current Data Parameters
NAME        Redzicka 1019
EXPNO                   2
PROCNO                  1

F2 - Acquisition Parameters
Date_            20210721
Time                21.12
INSTRUM             spect
PROBHD   5 mm BBI 1H/D-
PULPROG           zgpg30
TD                 65536
SOLVENT            CDCl3
NS                  5120
DS                     2
SWH           17985.611 Hz
FIDRES         0.274439 Hz
AQ            1.8219508 sec
RG              18390.4
DW               27.800 usec
DE                20.00 usec
TE                299.4 K
D1           2.00000000 sec
d11          0.03000000 sec
DELTA        1.89999998 sec
TD0                    1

======== CHANNEL f1 ========
NUC1                 13C
P1                 11.90 usec
PL1                -6.00 dB
SFO1          75.4803248 MHz

======== CHANNEL f2 ========
CPDPRG2          waltz16
NUC2                  1H
PCPD2             100.00 usec
PL2                 2.00 dB
PL12               21.66 dB
PL13               23.00 dB
SFO2         300.1512006 MHz

F2 - Processing parameters
SI                 32768
SF            75.4727782 MHz
WDW                   EM
SSB                    0
LB                  1.00 Hz
GB                     0
PC                  2.00
```

3f H

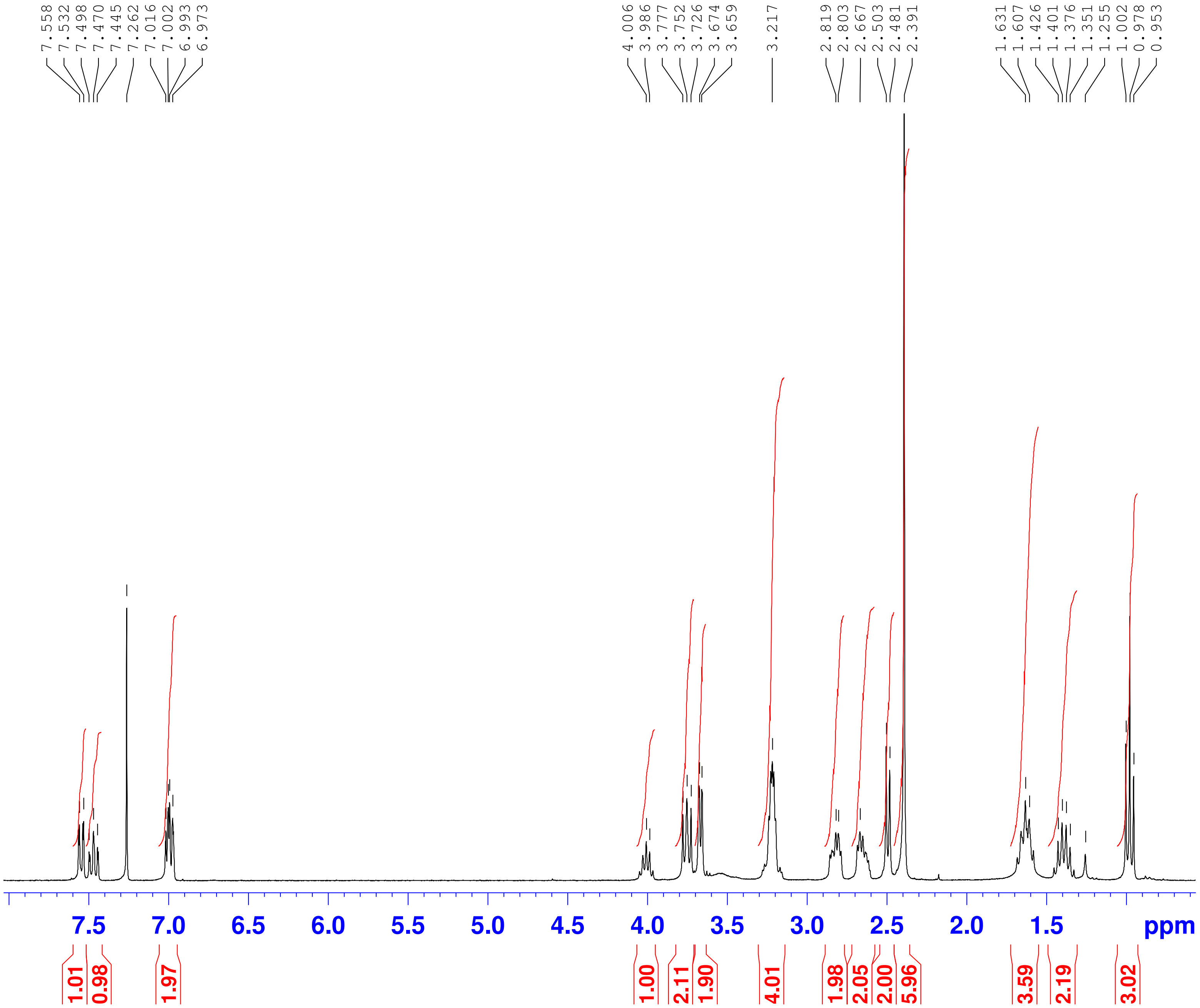

Current Data Parameters
NAME Redzicka 1019
EXPNO 1
PROCNO 1

F2 - Acquisition Parameters
Date_ 20210721
Time 15.38
INSTRUM spect
PROBHD 5 mm BBI 1H/D-
PULPROG zg30
TD 65536
SOLVENT CDCl3
NS 16
DS 0
SWH 6172.839 Hz
FIDRES 0.094190 Hz
AQ 5.3084660 sec
RG 406.4
DW 81.000 usec
DE 8.00 usec
TE 298.2 K
D1 1.00000000 sec
TD0 1

======== CHANNEL f1 ========
NUC1 1H
P1 10.40 usec
PL1 2.00 dB
SFO1 300.1518535 MHz

F2 - Processing parameters
SI 32768
SF 300.1500045 MHz
WDW EM
SSB 0
LB 0.30 Hz
GB 0
PC 20.00

3g C

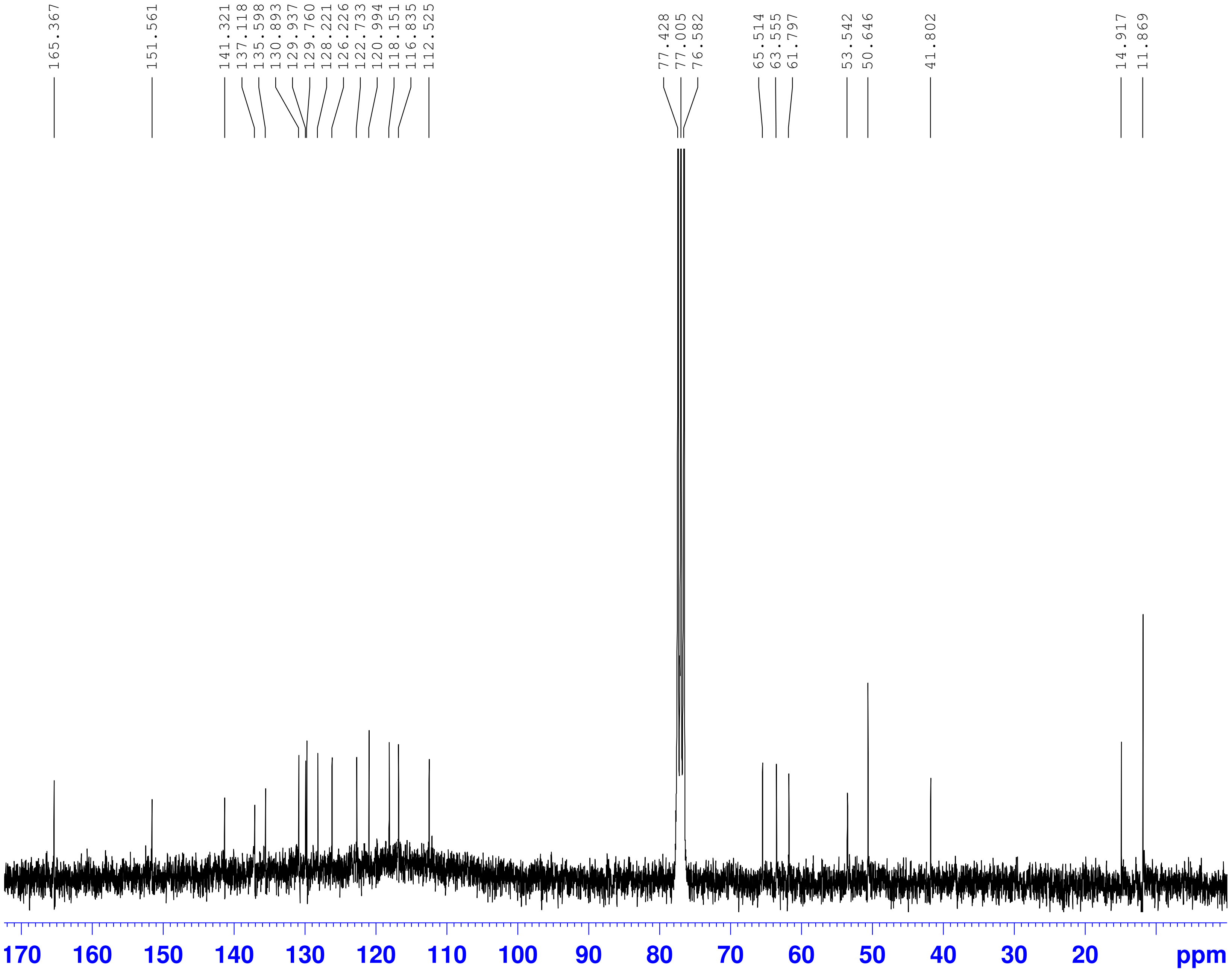

Current Data Parameters
NAME Redzicka 1010
EXPNO 2
PROCNO 1

F2 - Acquisition Parameters
Date_ 20210630
Time 22.16
INSTRUM spect
PROBHD 5 mm BBI 1H/D-
PULPROG zgpg30
TD 65536
SOLVENT CDCl3
NS 5120
DS 2
SWH 17985.611 Hz
FIDRES 0.274439 Hz
AQ 1.8219508 sec
RG 14596.5
DW 27.800 usec
DE 20.00 usec
TE 299.3 K
D1 2.00000000 sec
d11 0.03000000 sec
DELTA 1.89999998 sec
TD0 1

======== CHANNEL f1 ========
NUC1 13C
P1 11.90 usec
PL1 -6.00 dB
SFO1 75.4803248 MHz

======== CHANNEL f2 ========
CPDPRG2 waltz16
NUC2 1H
PCPD2 100.00 usec
PL2 2.00 dB
PL12 21.66 dB
PL13 23.00 dB
SFO2 300.1512006 MHz

F2 - Processing parameters
SI 32768
SF 75.4727782 MHz
WDW EM
SSB 0
LB 1.00 Hz
GB 0
PC 2.00

3g H

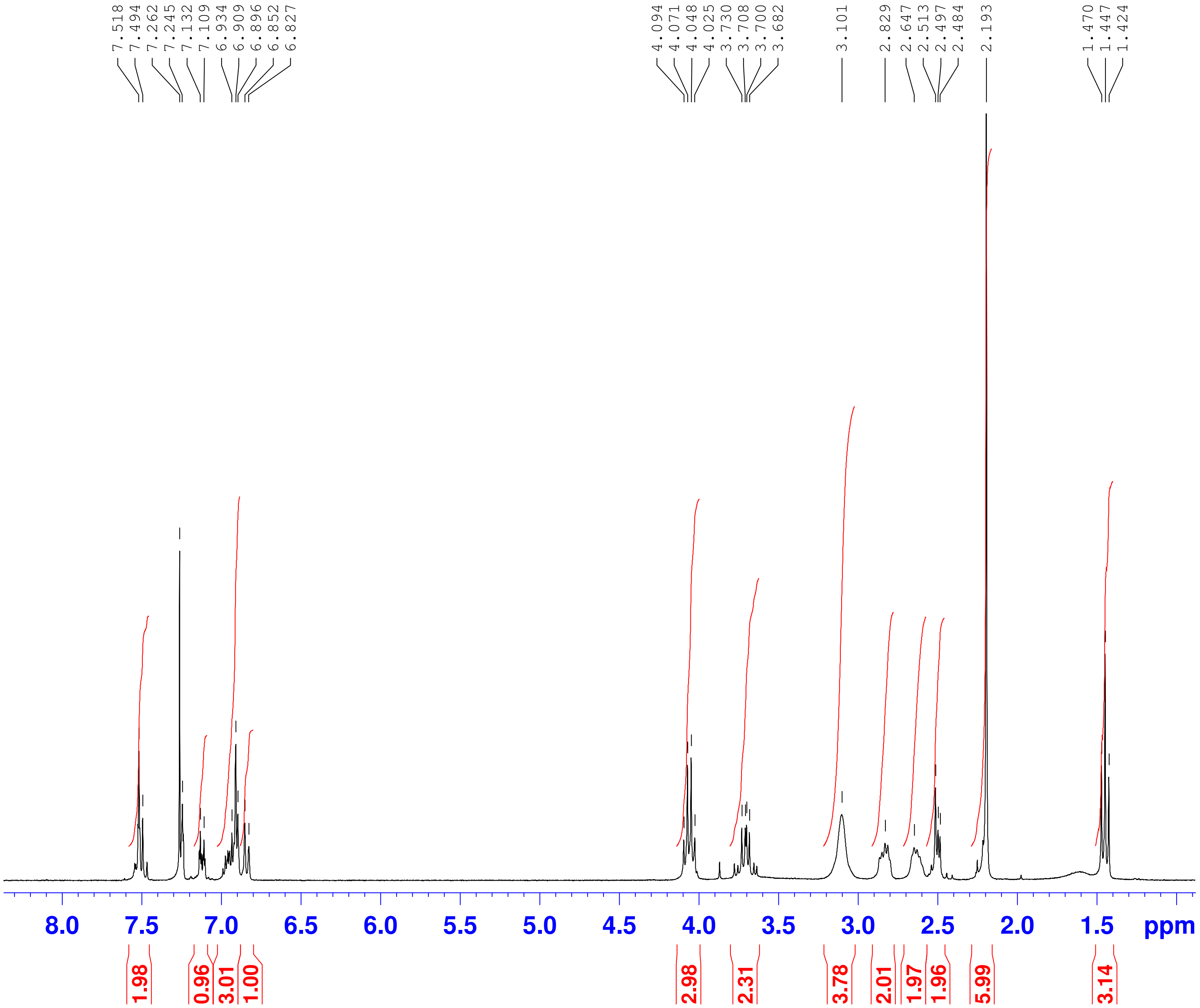

BRUKER

Current Data Parameters
NAME Redzicka 1010
EXPNO 1
PROCNO 1

F2 - Acquisition Parameters
Date_ 20210630
Time 16.41
INSTRUM spect
PROBHD 5 mm BBI 1H/D-
PULPROG zg30
TD 65536
SOLVENT CDCl3
NS 16
DS 0
SWH 6172.839 Hz
FIDRES 0.094190 Hz
AQ 5.3084660 sec
RG 512
DW 81.000 usec
DE 8.00 usec
TE 297.9 K
D1 1.00000000 sec
TD0 1

======== CHANNEL f1 ========
NUC1 1H
P1 10.40 usec
PL1 2.00 dB
SFO1 300.1518535 MHz

F2 - Processing parameters
SI 32768
SF 300.1500045 MHz
WDW EM
SSB 0
LB 0.30 Hz
GB 0
PC 20.00

3h C

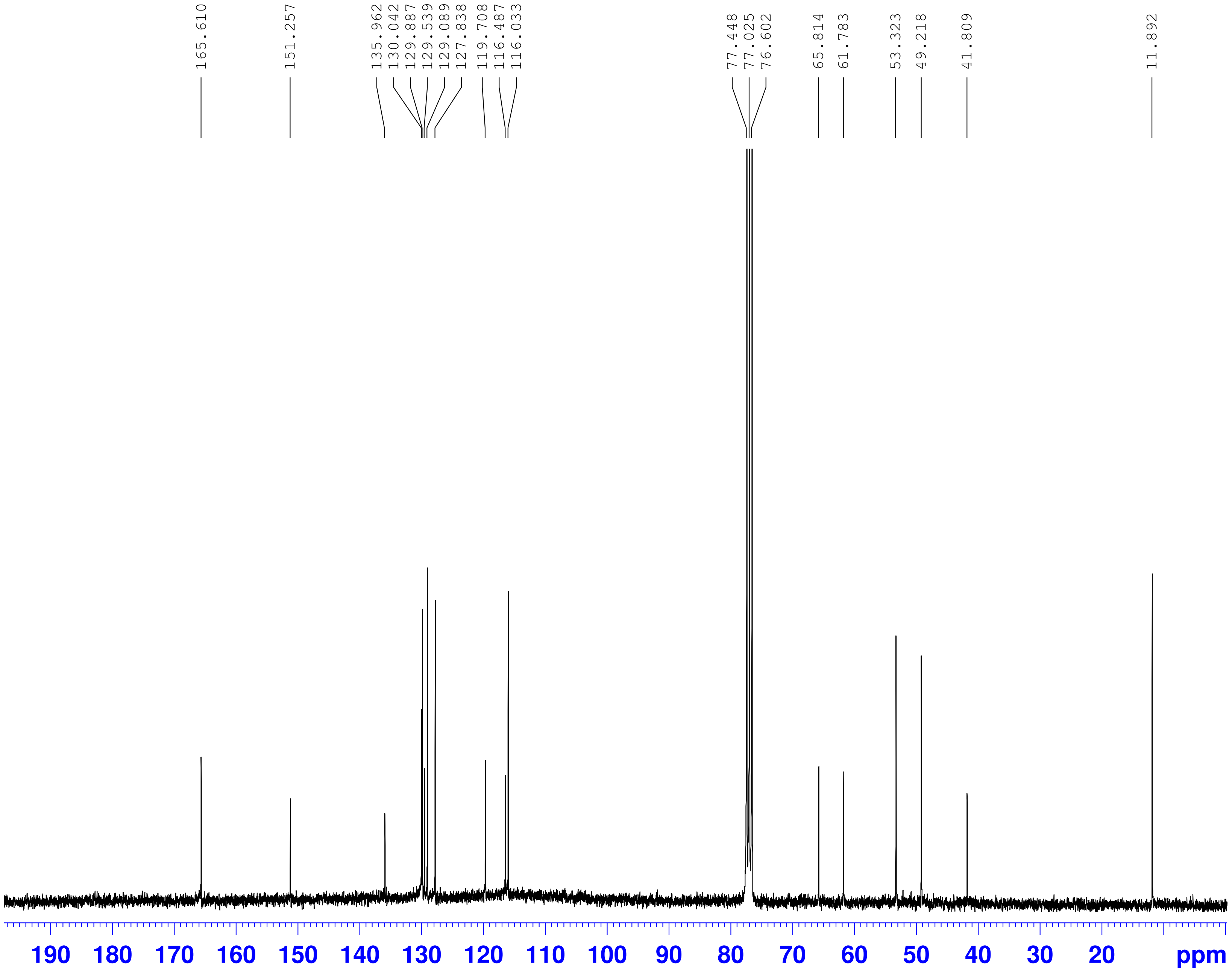

Current Data Parameters
NAME Redzicka 1021
EXPNO 2
PROCNO 1

F2 - Acquisition Parameters
Date_ 20210722
Time 22.46
INSTRUM spect
PROBHD 5 mm BBI 1H/D-
PULPROG zgpg30
TD 65536
SOLVENT CDCl3
NS 5120
DS 2
SWH 17985.611 Hz
FIDRES 0.274439 Hz
AQ 1.8219508 sec
RG 14596.5
DW 27.800 usec
DE 20.00 usec
TE 299.3 K
D1 2.00000000 sec
d11 0.03000000 sec
DELTA 1.89999998 sec
TD0 1

======== CHANNEL f1 ========
NUC1 13C
P1 11.90 usec
PL1 -6.00 dB
SFO1 75.4803248 MHz

======== CHANNEL f2 ========
CPDPRG2 waltz16
NUC2 1H
PCPD2 100.00 usec
PL2 2.00 dB
PL12 21.66 dB
PL13 23.00 dB
SFO2 300.1512006 MHz

F2 - Processing parameters
SI 32768
SF 75.4727782 MHz
WDW EM
SSB 0
LB 1.00 Hz
GB 0
PC 2.00

3h H

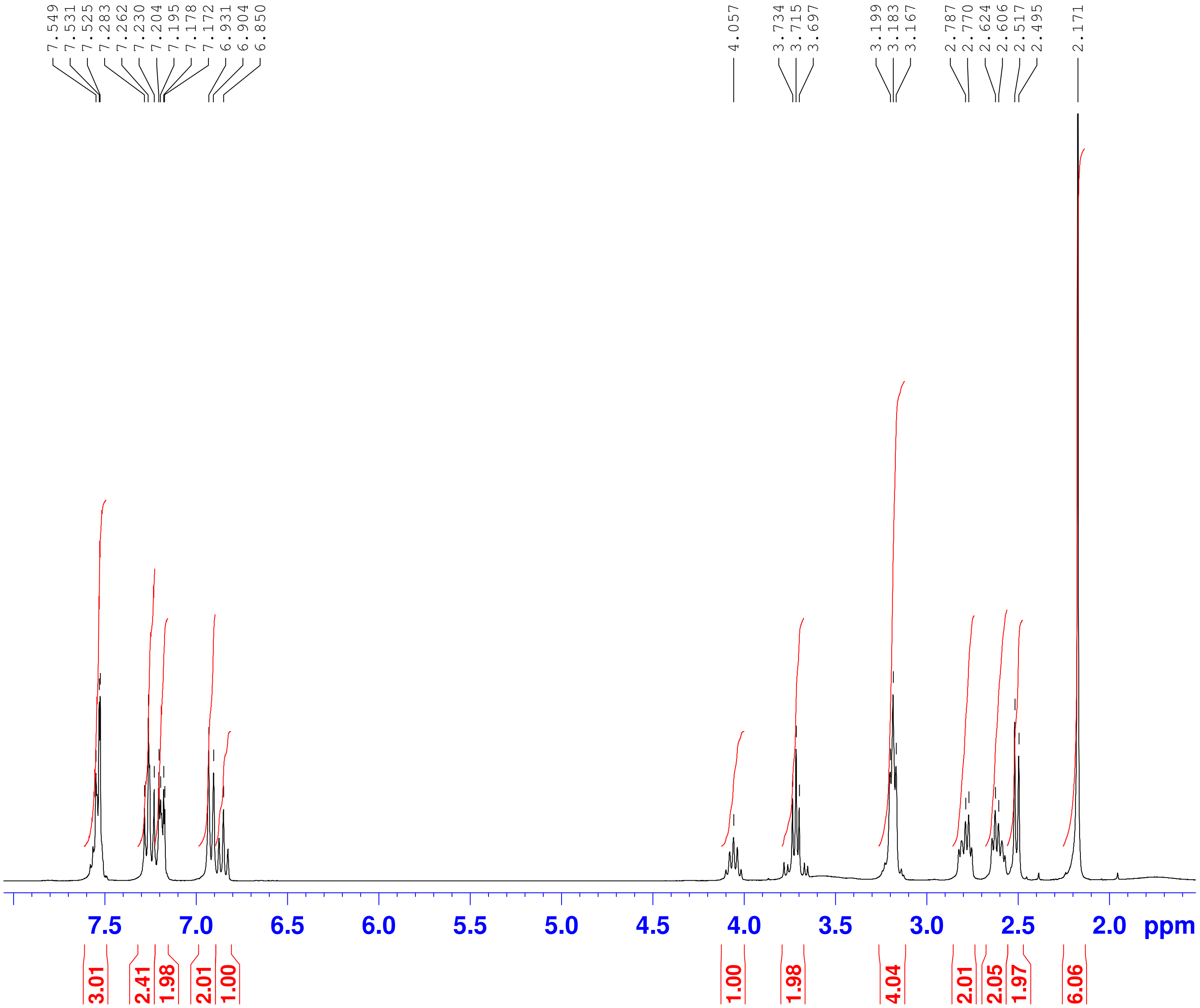

BRUKER

```
Current Data Parameters
NAME        Redzicka 1021
EXPNO                   1
PROCNO                  1

F2 - Acquisition Parameters
Date_            20210722
Time                17.11
INSTRUM             spect
PROBHD      5 mm BBI 1H/D-
PULPROG              zg30
TD                  65536
SOLVENT             CDCl3
NS                     16
DS                      0
SWH              6172.839 Hz
FIDRES           0.094190 Hz
AQ              5.3084660 sec
RG                    181
DW                 81.000 usec
DE                   8.00 usec
TE                  298.3 K
D1             1.00000000 sec
TD0                     1

======== CHANNEL f1 ========
NUC1                   1H
P1                  10.40 usec
PL1                  2.00 dB
SFO1          300.1518535 MHz

F2 - Processing parameters
SI                  32768
SF            300.1500045 MHz
WDW                    EM
SSB                     0
LB                   0.30 Hz
GB                      0
PC                  20.00
```

3i C

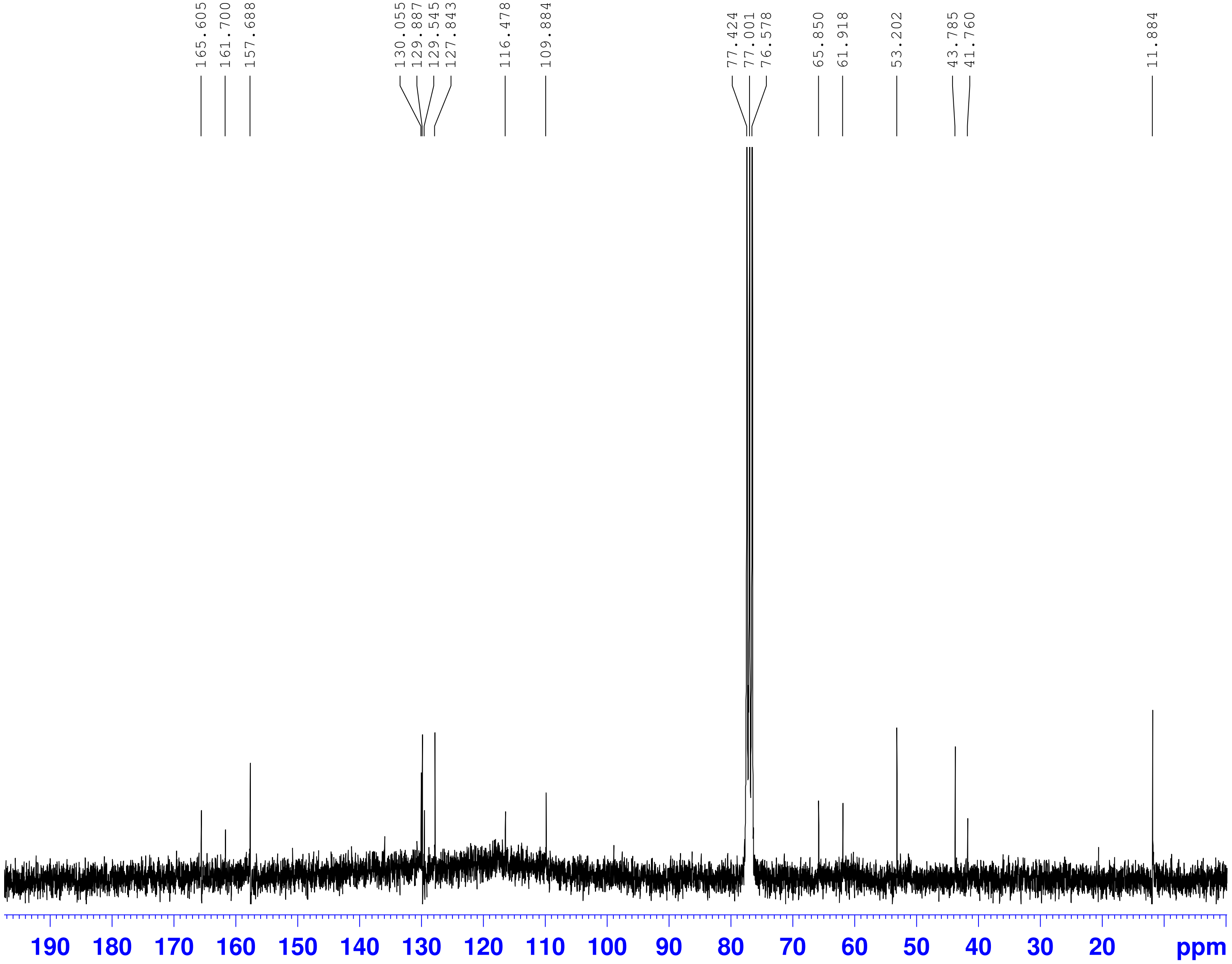

Current Data Parameters
NAME Redzicka 1011
EXPNO 2
PROCNO 1

F2 - Acquisition Parameters
Date_ 20210701
Time 14.45
INSTRUM spect
PROBHD 5 mm BBI 1H/D-
PULPROG zgpg30
TD 65536
SOLVENT CDCl3
NS 5120
DS 2
SWH 17985.611 Hz
FIDRES 0.274439 Hz
AQ 1.8219508 sec
RG 18390.4
DW 27.800 usec
DE 20.00 usec
TE 299.3 K
D1 2.00000000 sec
d11 0.03000000 sec
DELTA 1.89999998 sec
TD0 1

======== CHANNEL f1 ========
NUC1 13C
P1 11.90 usec
PL1 -6.00 dB
SFO1 75.4803248 MHz

======== CHANNEL f2 ========
CPDPRG2 waltz16
NUC2 1H
PCPD2 100.00 usec
PL2 2.00 dB
PL12 21.66 dB
PL13 23.00 dB
SFO2 300.1512006 MHz

F2 - Processing parameters
SI 32768
SF 75.4727782 MHz
WDW EM
SSB 0
LB 1.00 Hz
GB 0
PC 2.00

3i H

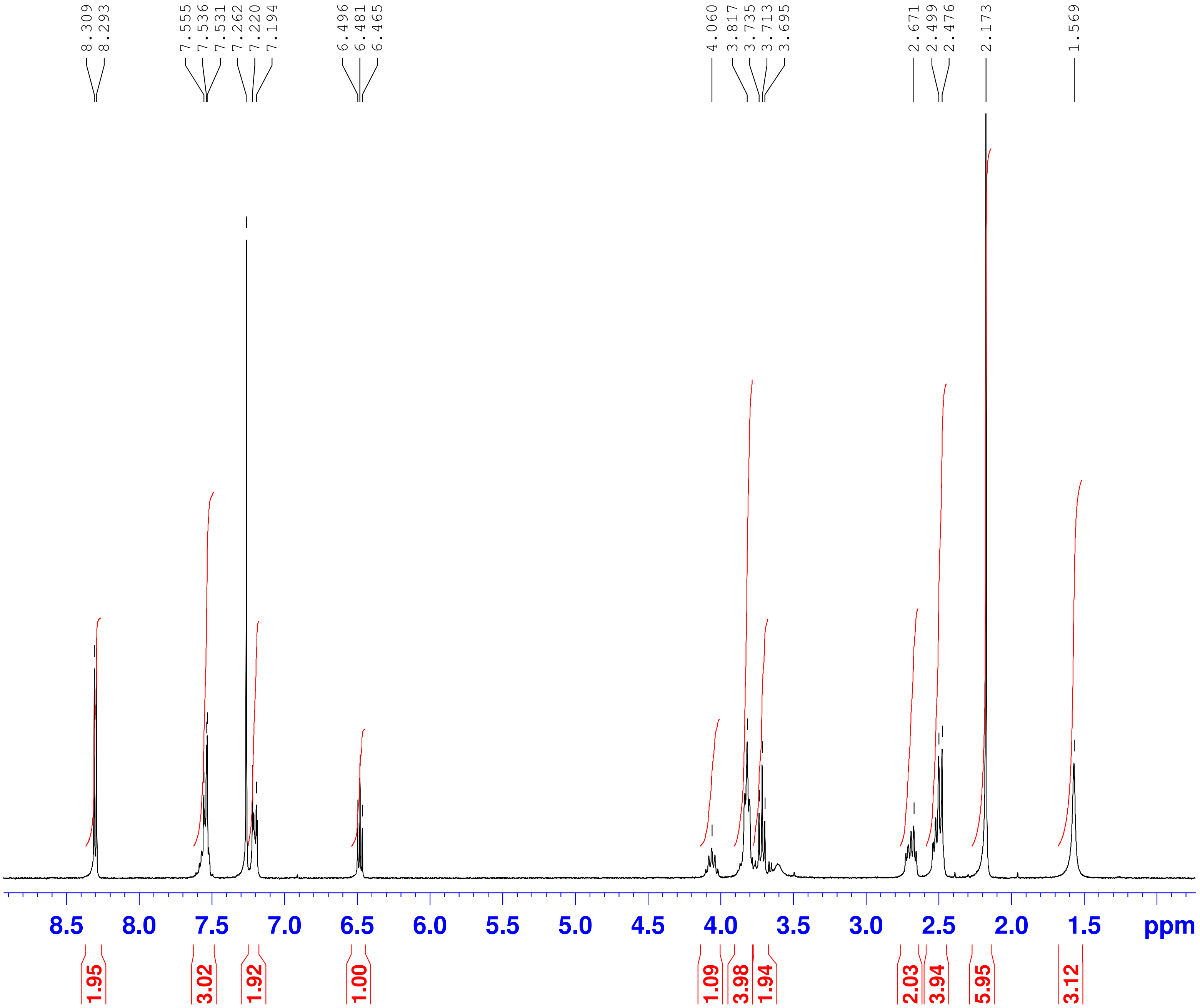


```
Current Data Parameters
NAME       Redzicka 1011
EXPNO                  1
PROCNO                 1

F2 - Acquisition Parameters
Date_           20210701
Time                9.11
INSTRUM            spect
PROBHD   5 mm BBI 1H/D-
PULPROG             zg30
TD                 65536
SOLVENT            CDCl3
NS                    16
DS                     0
SWH             6172.839 Hz
FIDRES          0.094190 Hz
AQ             5.3084660 sec
RG                 812.7
DW                81.000 usec
DE                  8.00 usec
TE                 298.4 K
D1            1.00000000 sec
TD0                    1

======== CHANNEL f1 ========
NUC1                  1H
P1                 10.40 usec
PL1                 2.00 dB
SFO1         300.1518535 MHz

F2 - Processing parameters
SI                 32768
SF           300.1500045 MHz
WDW                   EM
SSB                    0
LB                  0.30 Hz
GB                     0
PC                 20.00
```

3j C

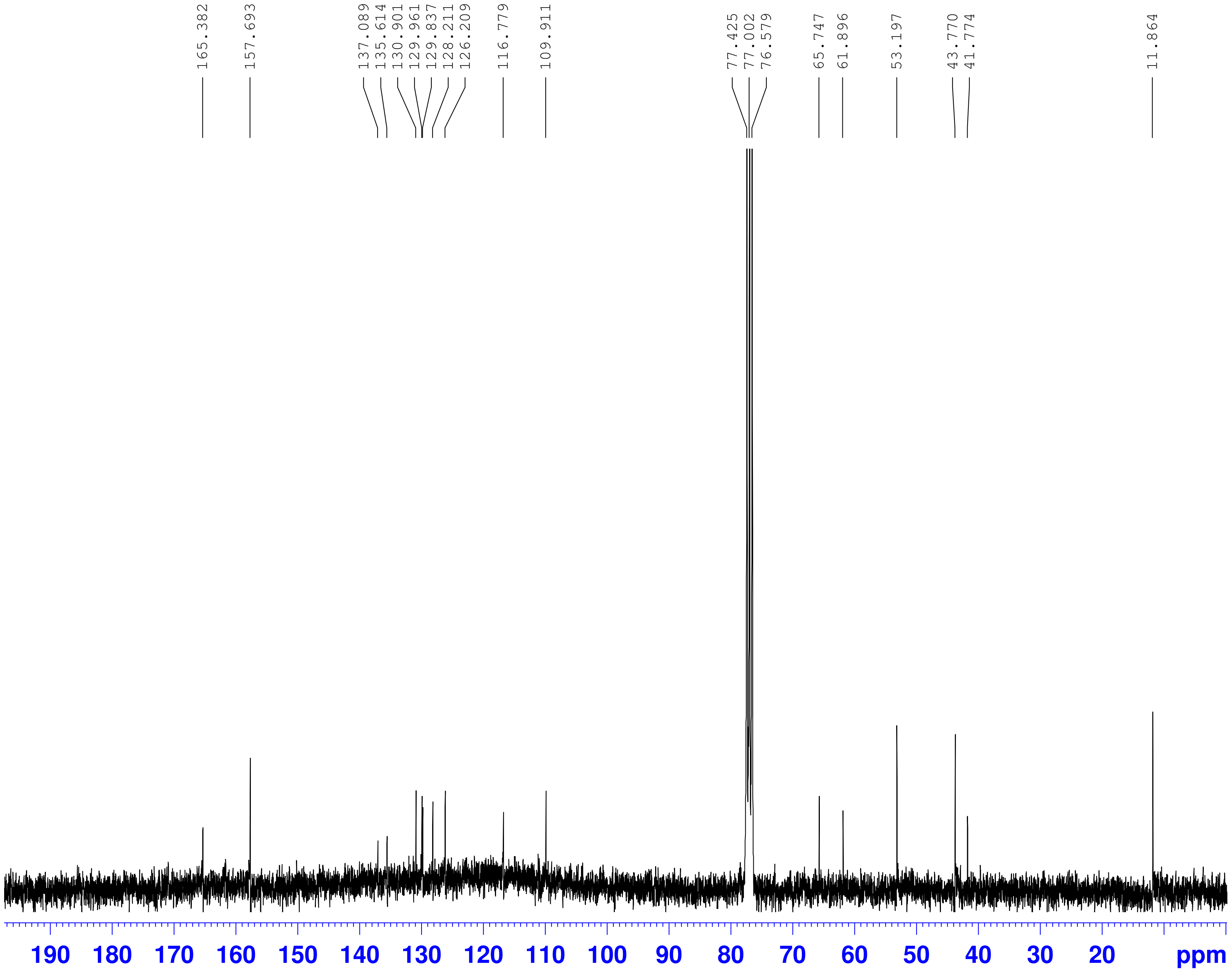

BRUKER

Current Data Parameters		
NAME	Redzicka 1013	
EXPNO	2	
PROCNO	1	
F2 - Acquisition Parameters		
Date_	20210702	
Time	14.41	
INSTRUM	spect	
PROBHD	5 mm BBI 1H/D-	
PULPROG	zgpg30	
TD	65536	
SOLVENT	CDCl3	
NS	5120	
DS	2	
SWH	17985.611	Hz
FIDRES	0.274439	Hz
AQ	1.8219508	sec
RG	14596.5	
DW	27.800	usec
DE	20.00	usec
TE	299.4	K
D1	2.00000000	sec
d11	0.03000000	sec
DELTA	1.89999998	sec
TD0	1	
======== CHANNEL f1 ========		
NUC1	13C	
P1	11.90	usec
PL1	-6.00	dB
SFO1	75.4803248	MHz
======== CHANNEL f2 ========		
CPDPRG2	waltz16	
NUC2	1H	
PCPD2	100.00	usec
PL2	2.00	dB
PL12	21.66	dB
PL13	23.00	dB
SFO2	300.1512006	MHz
F2 - Processing parameters		
SI	32768	
SF	75.4727782	MHz
WDW	EM	
SSB	0	
LB	1.00	Hz
GB	0	
PC	2.00	

3j H

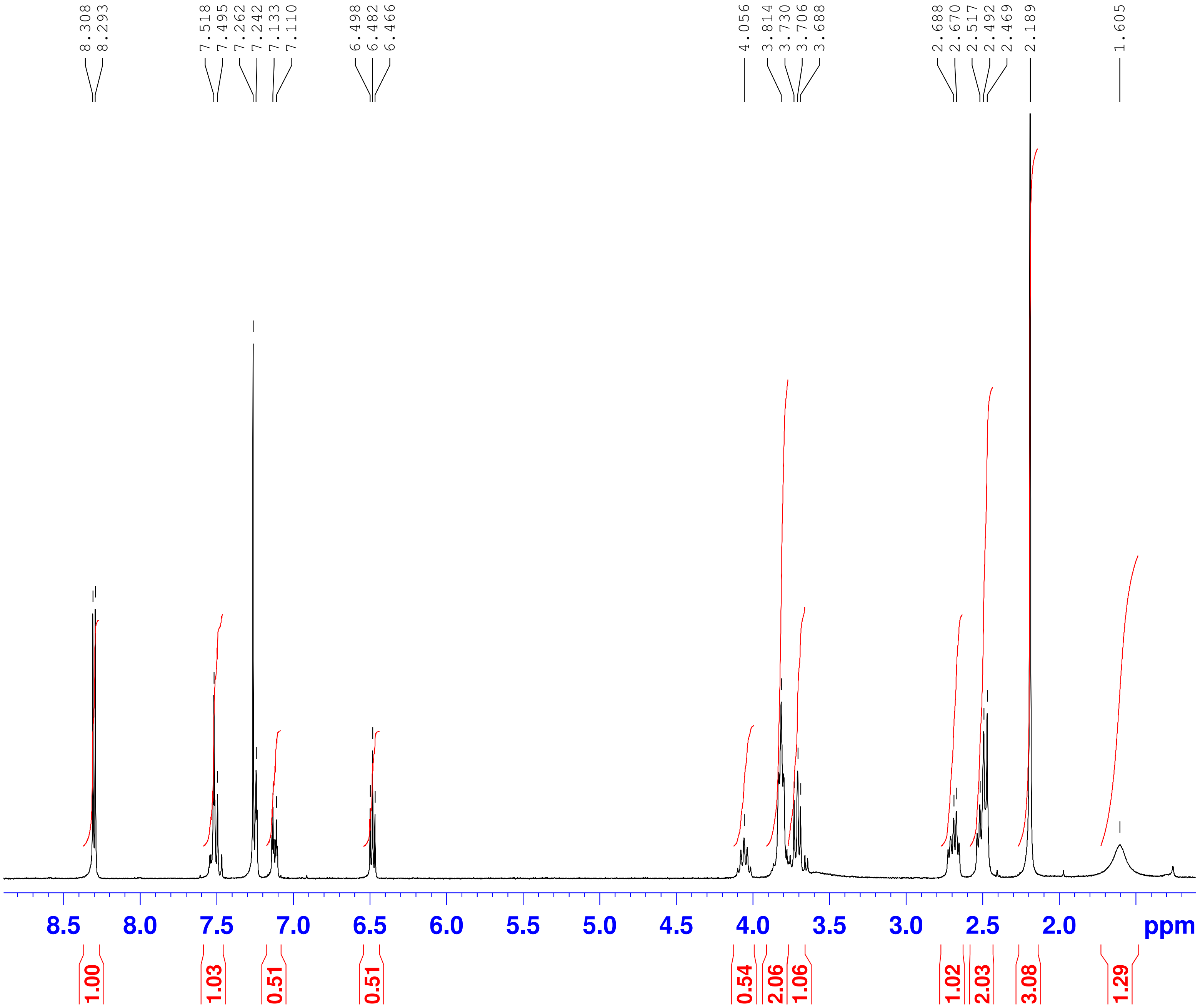

BRUKER

Current Data Parameters
NAME Redzicka 1013
EXPNO 1
PROCNO 1

F2 - Acquisition Parameters
Date_ 20210702
Time 9.07
INSTRUM spect
PROBHD 5 mm BBI 1H/D-
PULPROG zg30
TD 65536
SOLVENT CDCl3
NS 16
DS 0
SWH 6172.839 Hz
FIDRES 0.094190 Hz
AQ 5.3084660 sec
RG 645.1
DW 81.000 usec
DE 8.00 usec
TE 298.2 K
D1 1.00000000 sec
TD0 1

======== CHANNEL f1 ========
NUC1 1H
P1 10.40 usec
PL1 2.00 dB
SFO1 300.1518535 MHz

F2 - Processing parameters
SI 32768
SF 300.1500045 MHz
WDW EM
SSB 0
LB 0.30 Hz
GB 0
PC 20.00

3k C

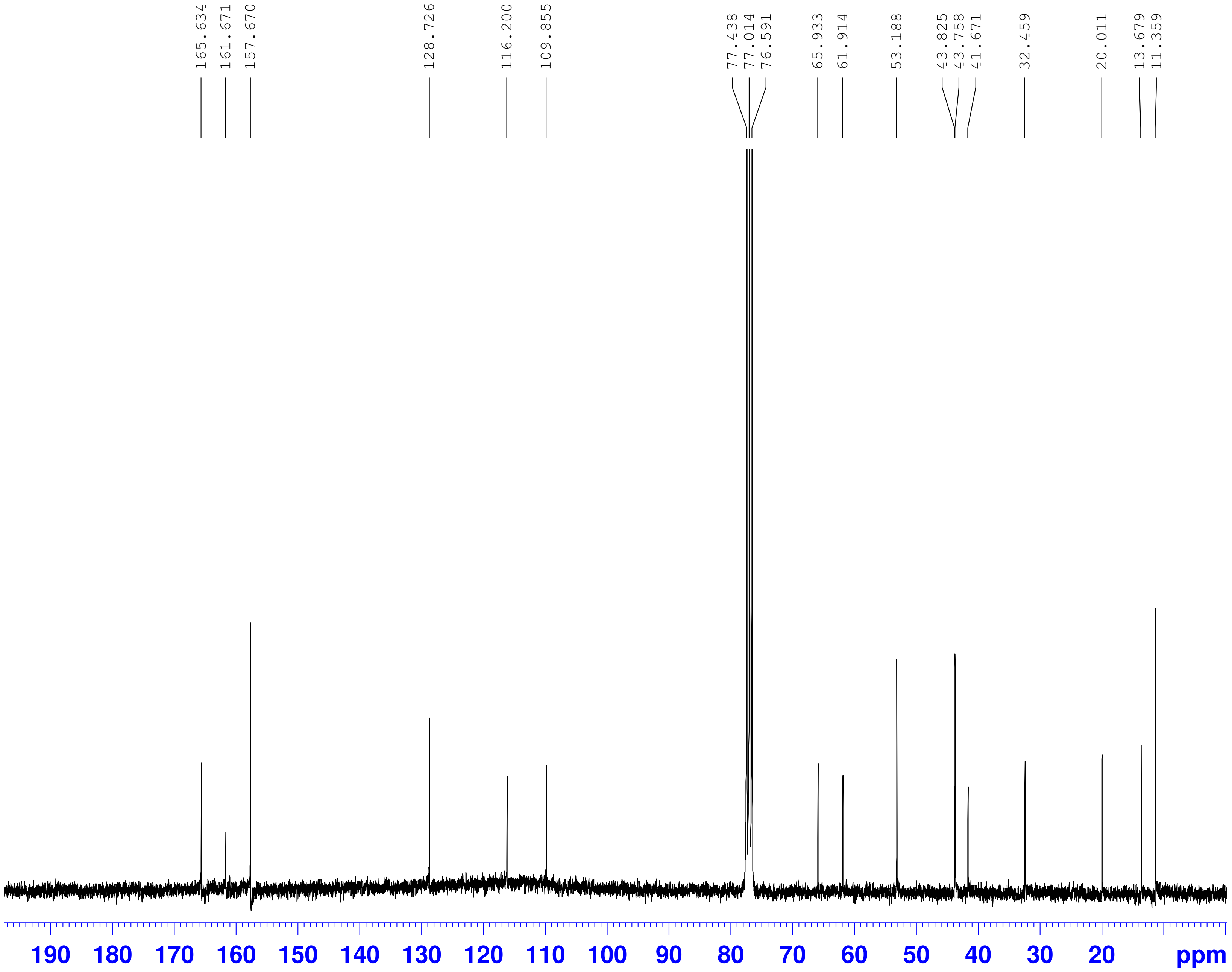

BRUKER

Current Data Parameters
NAME Redzicka 1014
EXPNO 2
PROCNO 1

F2 - Acquisition Parameters
Date_ 20210719
Time 15.15
INSTRUM spect
PROBHD 5 mm BBI 1H/D-
PULPROG zgpg30
TD 65536
SOLVENT CDCl3
NS 5120
DS 2
SWH 17985.611 Hz
FIDRES 0.274439 Hz
AQ 1.8219508 sec
RG 16384
DW 27.800 usec
DE 20.00 usec
TE 299.3 K
D1 2.00000000 sec
d11 0.03000000 sec
DELTA 1.89999998 sec
TD0 1

======== CHANNEL f1 ========
NUC1 13C
P1 11.90 usec
PL1 -6.00 dB
SFO1 75.4803248 MHz

======== CHANNEL f2 ========
CPDPRG2 waltz16
NUC2 1H
PCPD2 100.00 usec
PL2 2.00 dB
PL12 21.66 dB
PL13 23.00 dB
SFO2 300.1512006 MHz

F2 - Processing parameters
SI 32768
SF 75.4727782 MHz
WDW EM
SSB 0
LB 1.00 Hz
GB 0
PC 2.00

3k H

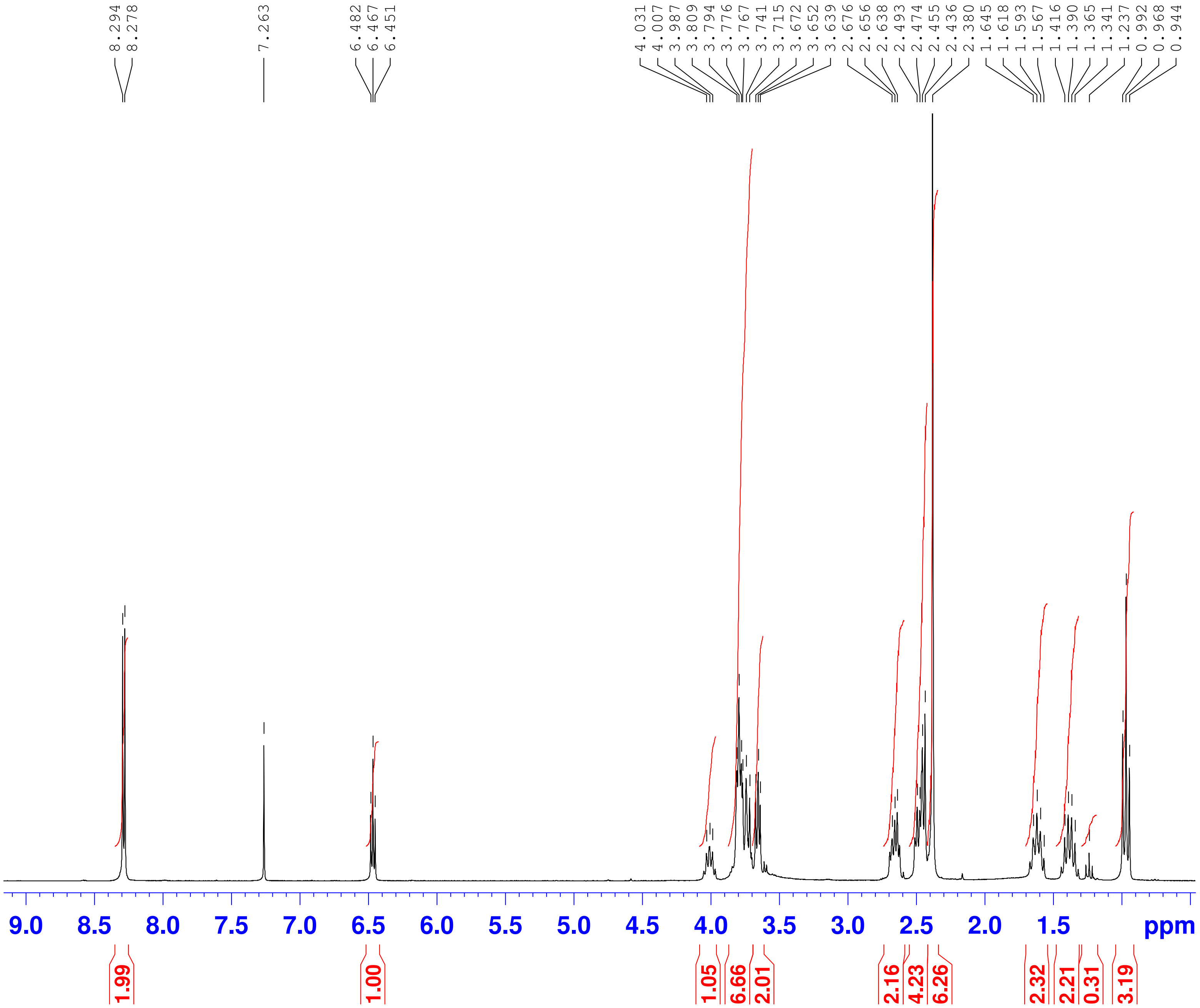

BRUKER

Current Data Parameters
NAME Redzicka 1014
EXPNO 1
PROCNO 1

F2 - Acquisition Parameters
Date_ 20210719
Time 9.36
INSTRUM spect
PROBHD 5 mm BBI 1H/D-
PULPROG zg30
TD 65536
SOLVENT CDCl3
NS 16
DS 0
SWH 6172.839 Hz
FIDRES 0.094190 Hz
AQ 5.3084660 sec
RG 203.2
DW 81.000 usec
DE 8.00 usec
TE 298.1 K
D1 1.00000000 sec
TD0 1

======== CHANNEL f1 ========
NUC1 1H
P1 10.40 usec
PL1 2.00 dB
SFO1 300.1518535 MHz

F2 - Processing parameters
SI 32768
SF 300.1500045 MHz
WDW EM
SSB 0
LB 0.30 Hz
GB 0
PC 20.00

31 C

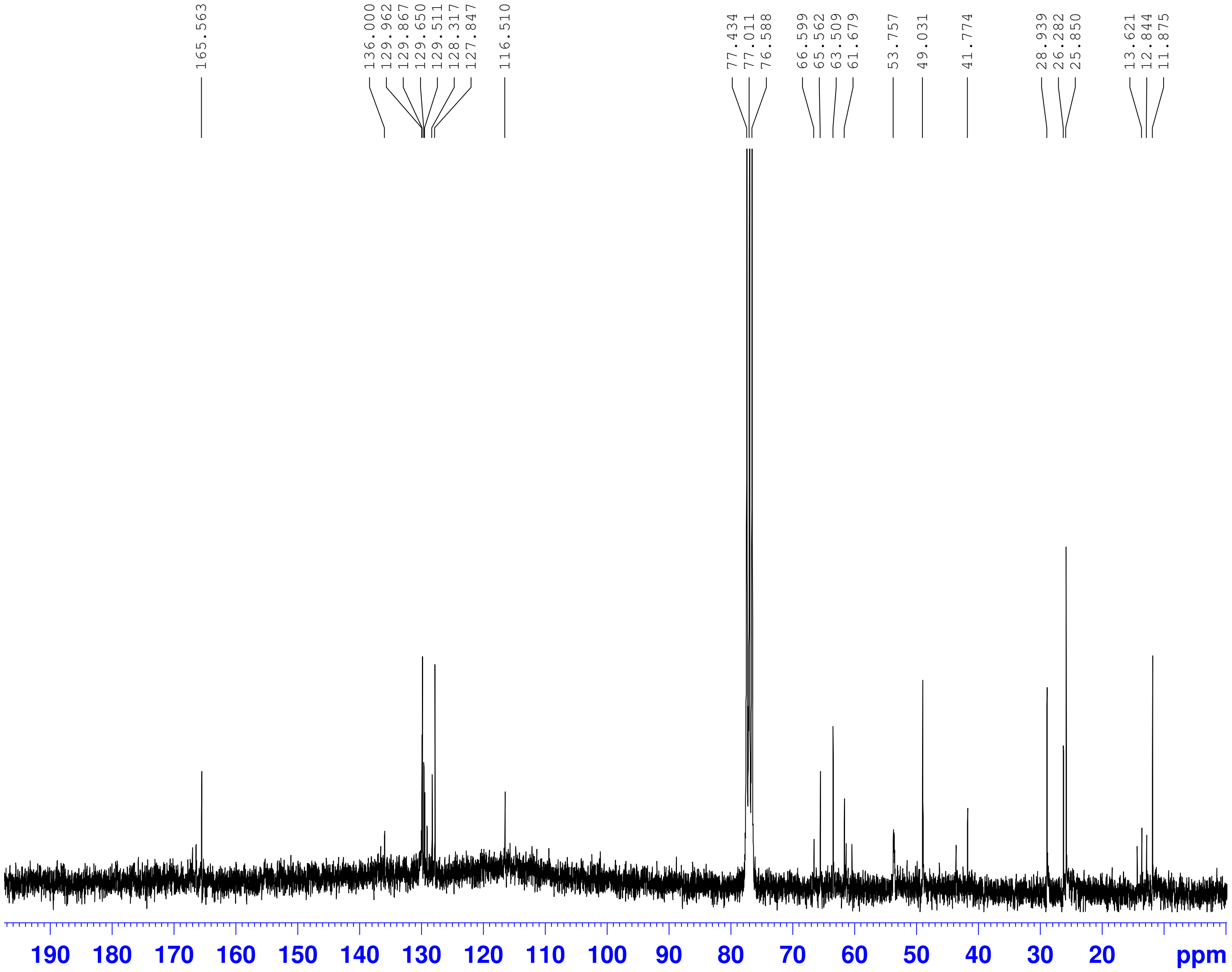

Current Data Parameters
NAME Redzicka 1012
EXPNO 2
PROCNO 1

F2 - Acquisition Parameters
Date_ 20210701
Time 20.42
INSTRUM spect
PROBHD 5 mm BBI 1H/D-
PULPROG zgpg30
TD 65536
SOLVENT CDCl3
NS 5120
DS 2
SWH 17985.611 Hz
FIDRES 0.274439 Hz
AQ 1.8219508 sec
RG 29193
DW 27.800 usec
DE 20.00 usec
TE 299.3 K
D1 2.00000000 sec
d11 0.03000000 sec
DELTA 1.89999998 sec
TD0 1

======== CHANNEL f1 ========
NUC1 13C
P1 11.90 usec
PL1 -6.00 dB
SFO1 75.4803248 MHz

======== CHANNEL f2 ========
CPDPRG2 waltz16
NUC2 1H
PCPD2 100.00 usec
PL2 2.00 dB
PL12 21.66 dB
PL13 23.00 dB
SFO2 300.1512006 MHz

F2 - Processing parameters
SI 32768
SF 75.4727782 MHz
WDW EM
SSB 0
LB 1.00 Hz
GB 0
PC 2.00

31 H

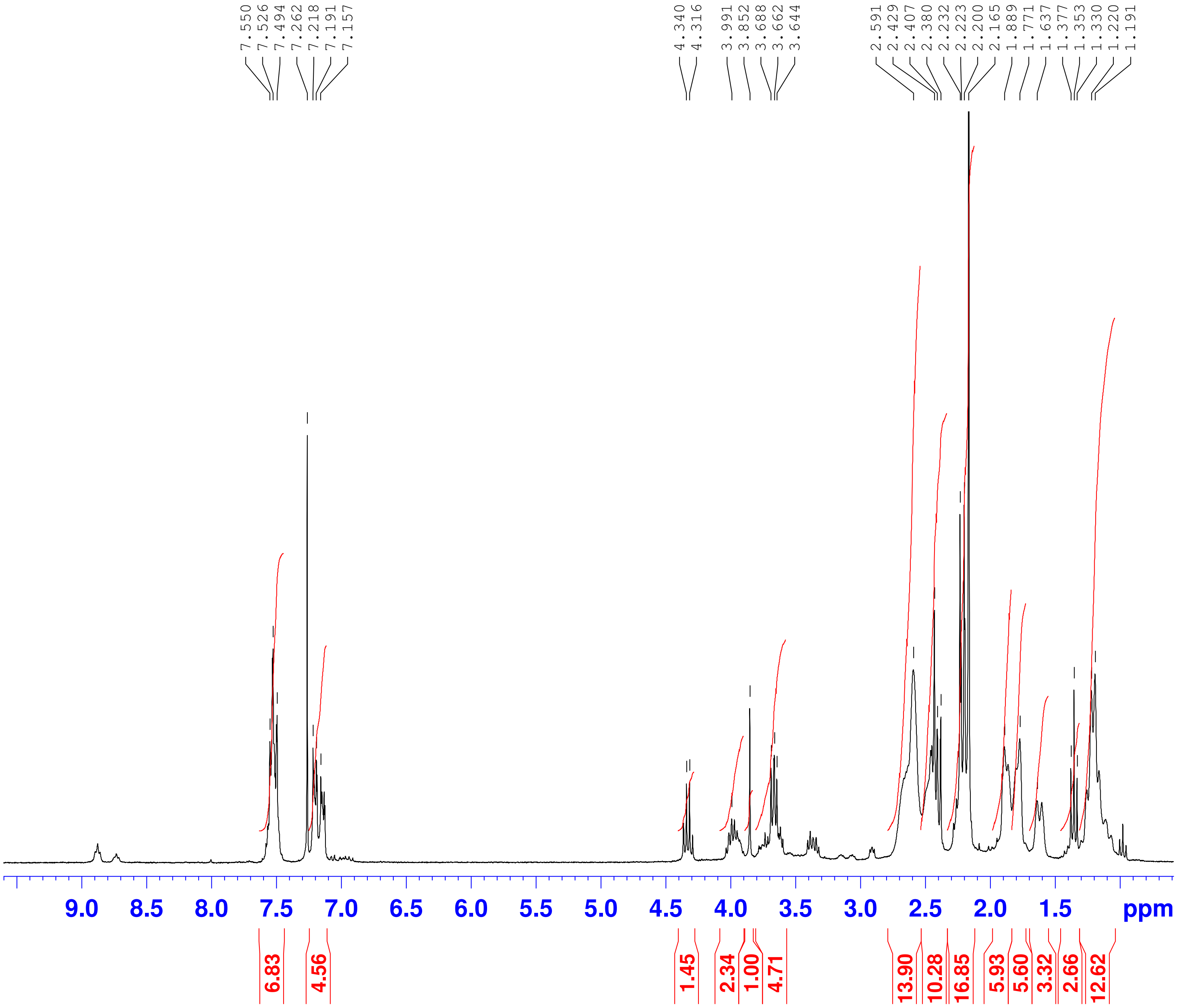


```
Current Data Parameters
NAME        Redzicka 1012
EXPNO                   1
PROCNO                  1

F2 - Acquisition Parameters
Date_            20210701
Time                15.07
INSTRUM             spect
PROBHD     5 mm BBI 1H/D-
PULPROG              zg30
TD                  65536
SOLVENT             CDCl3
NS                     16
DS                      0
SWH              6172.839 Hz
FIDRES           0.094190 Hz
AQ              5.3084660 sec
RG                    256
DW                 81.000 usec
DE                   8.00 usec
TE                  298.1 K
D1             1.00000000 sec
TD0                     1

======== CHANNEL f1 ========
NUC1                   1H
P1                  10.40 usec
PL1                  2.00 dB
SFO1          300.1518535 MHz

F2 - Processing parameters
SI                  32768
SF            300.1500045 MHz
WDW                    EM
SSB                     0
LB                   0.30 Hz
GB                      0
PC                  20.00
```

3m C

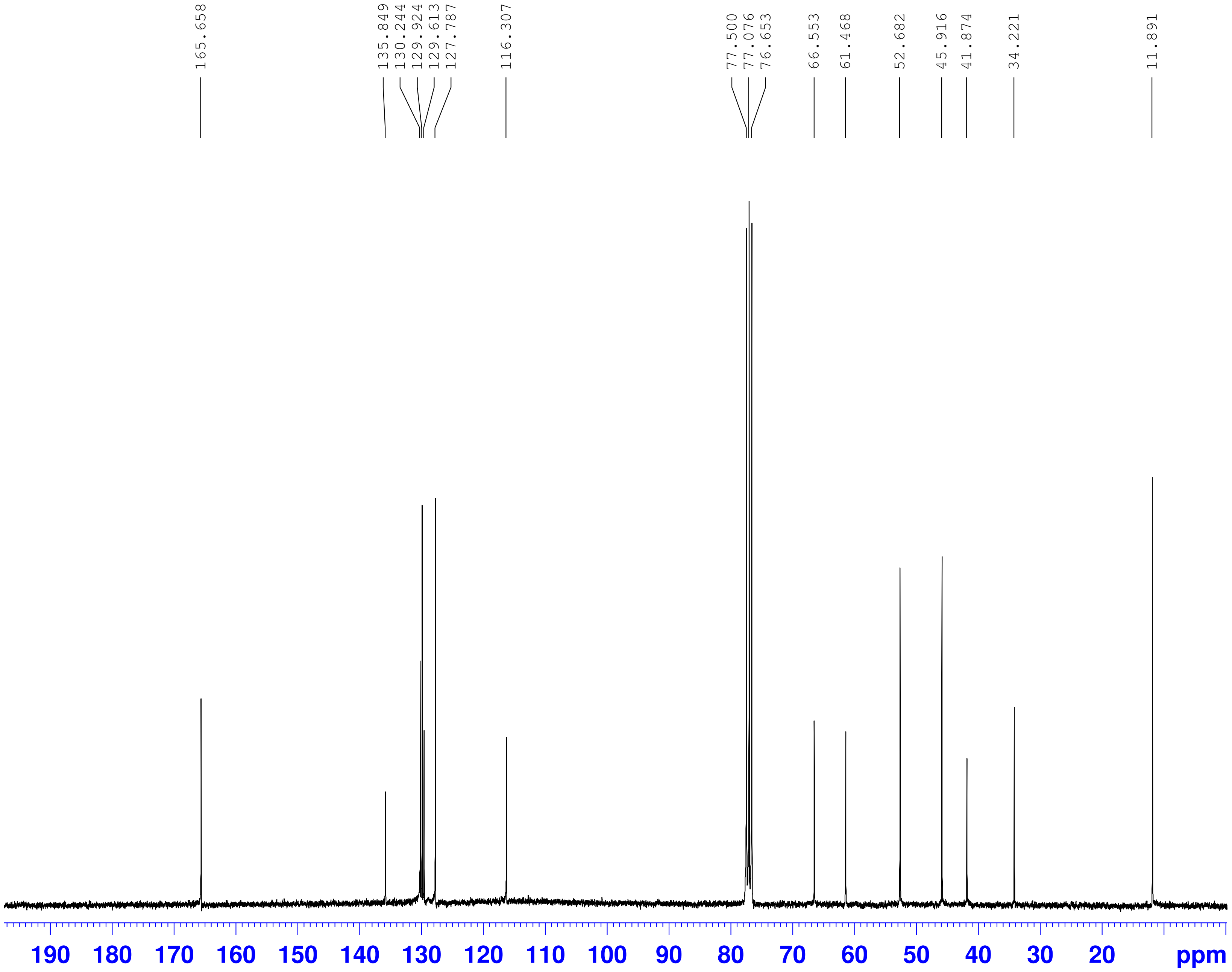

Current Data Parameters
NAME Redzicka 1017
EXPNO 2
PROCNO 1

F2 - Acquisition Parameters
Date_ 20210721
Time 2.01
INSTRUM spect
PROBHD 5 mm BBI 1H/D-
PULPROG zgpg30
TD 65536
SOLVENT CDCl3
NS 5120
DS 2
SWH 17985.611 Hz
FIDRES 0.274439 Hz
AQ 1.8219508 sec
RG 18390.4
DW 27.800 usec
DE 20.00 usec
TE 299.6 K
D1 2.00000000 sec
d11 0.03000000 sec
DELTA 1.89999998 sec
TD0 1

======== CHANNEL f1 ========
NUC1 13C
P1 11.90 usec
PL1 -6.00 dB
SFO1 75.4803248 MHz

======== CHANNEL f2 ========
CPDPRG2 waltz16
NUC2 1H
PCPD2 100.00 usec
PL2 2.00 dB
PL12 21.66 dB
PL13 23.00 dB
SFO2 300.1512006 MHz

F2 - Processing parameters
SI 32768
SF 75.4727782 MHz
WDW EM
SSB 0
LB 1.00 Hz
GB 0
PC 2.00

3m H

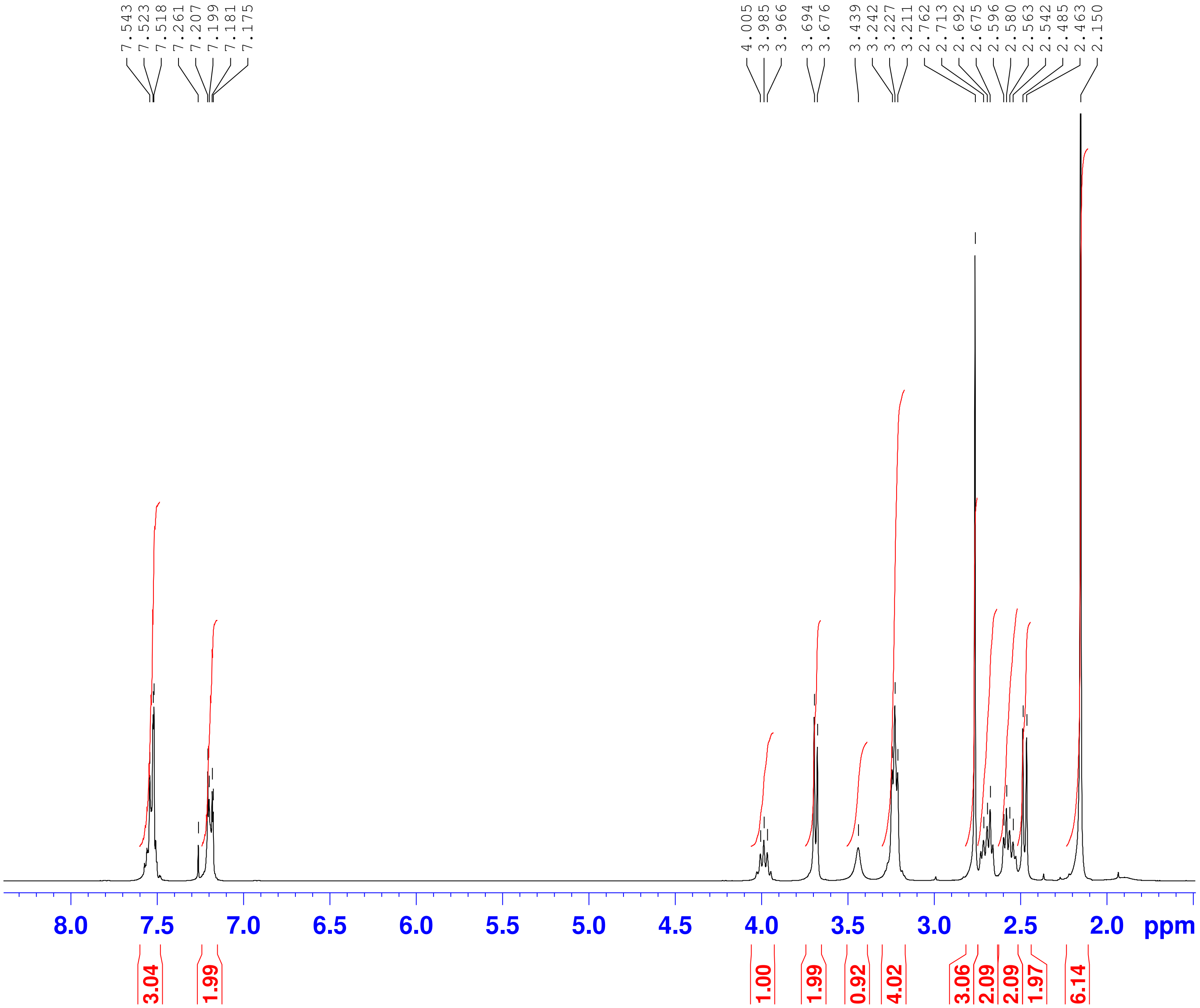

Current Data Parameters
NAME Redzicka 1017
EXPNO 1
PROCNO 1

F2 - Acquisition Parameters
Date_ 20210720
Time 20.25
INSTRUM spect
PROBHD 5 mm BBI 1H/D-
PULPROG zg30
TD 65536
SOLVENT CDCl3
NS 16
DS 0
SWH 6172.839 Hz
FIDRES 0.094190 Hz
AQ 5.3084660 sec
RG 71.8
DW 81.000 usec
DE 8.00 usec
TE 298.0 K
D1 1.00000000 sec
TD0 1

======== CHANNEL f1 ========
NUC1 1H
P1 10.40 usec
PL1 2.00 dB
SFO1 300.1518535 MHz

F2 - Processing parameters
SI 32768
SF 300.1500045 MHz
WDW EM
SSB 0
LB 0.30 Hz
GB 0
PC 20.00

3n C

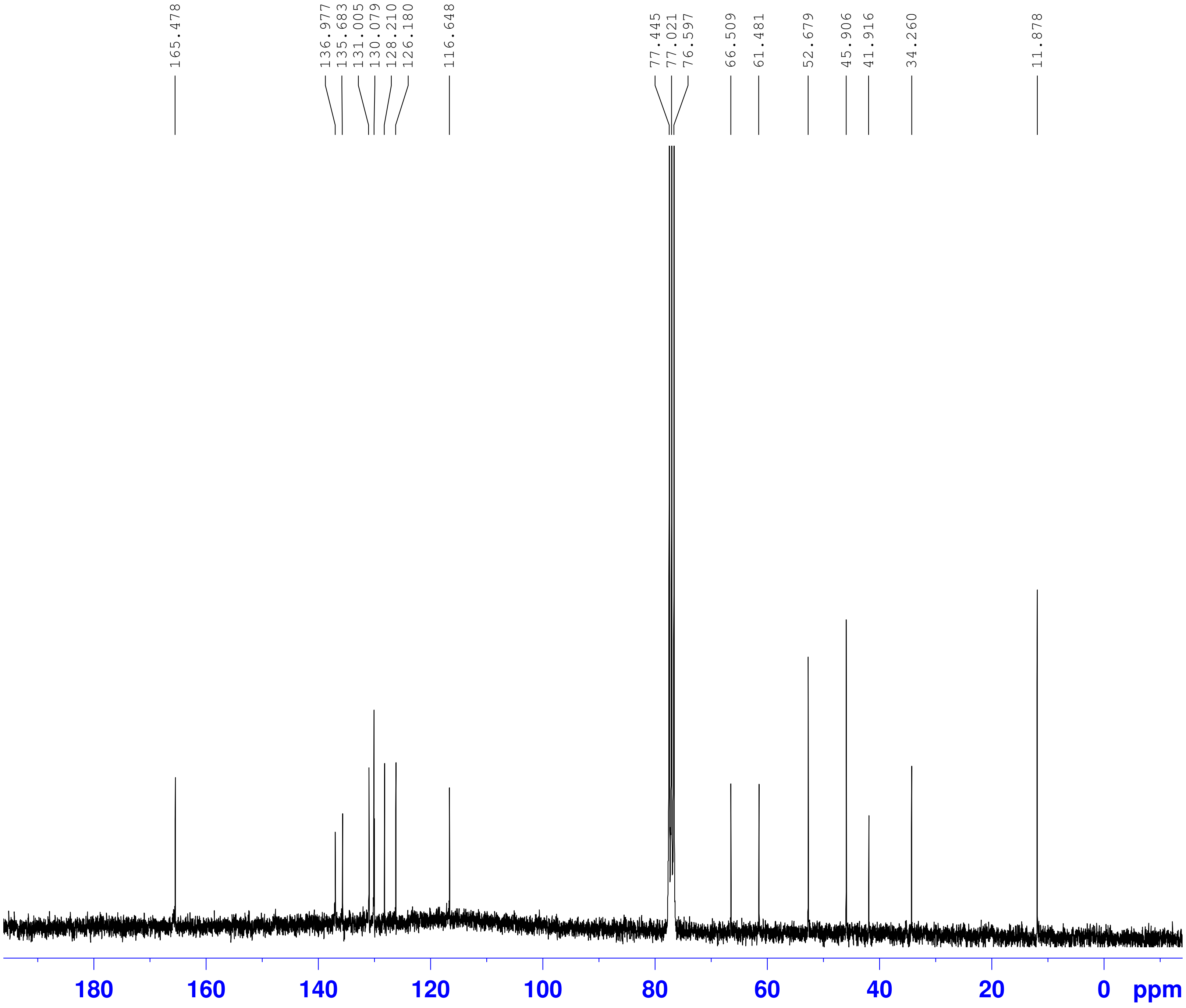

Current Data Parameters
NAME Redzicka 1113
EXPNO 3
PROCNO 1

F2 - Acquisition Parameters
Date_ 20210825
Time 15.35
INSTRUM spect
PROBHD 5 mm BBI 1H/D-
PULPROG zgpg30
TD 65536
SOLVENT CDCl3
NS 4096
DS 2
SWH 17985.611 Hz
FIDRES 0.274439 Hz
AQ 1.8219508 sec
RG 9195.2
DW 27.800 usec
DE 20.00 usec
TE 299.6 K
D1 2.00000000 sec
d11 0.03000000 sec
DELTA 1.89999998 sec
TD0 1

======== CHANNEL f1 ========
NUC1 13C
P1 11.90 usec
PL1 -6.00 dB
SFO1 75.4803248 MHz

======== CHANNEL f2 ========
CPDPRG2 waltz16
NUC2 1H
PCPD2 100.00 usec
PL2 2.00 dB
PL12 21.66 dB
PL13 23.00 dB
SFO2 300.1512006 MHz

F2 - Processing parameters
SI 32768
SF 75.4727782 MHz
WDW EM
SSB 0
LB 1.00 Hz
GB 0
PC 10.00

3n H

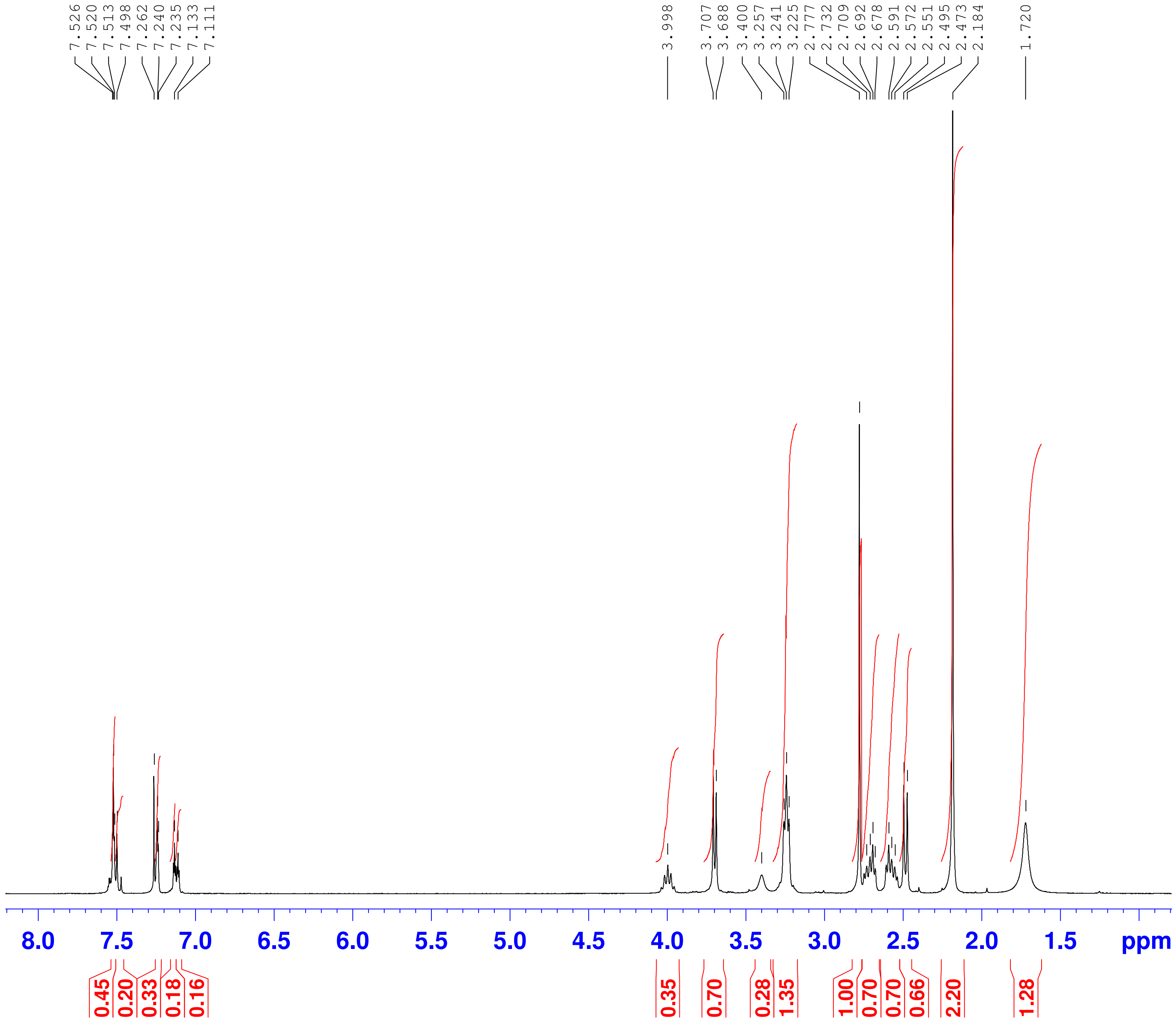

BRUKER

Current Data Parameters
NAME Redzicka 1113
EXPNO 2
PROCNO 1

F2 - Acquisition Parameters
Date_ 20210825
Time 11.05
INSTRUM spect
PROBHD 5 mm BBI 1H/D-
PULPROG zg30
TD 65536
SOLVENT CDCl3
NS 16
DS 0
SWH 6172.839 Hz
FIDRES 0.094190 Hz
AQ 5.3084660 sec
RG 181
DW 81.000 usec
DE 8.00 usec
TE 298.5 K
D1 1.00000000 sec
TD0 1

======== CHANNEL f1 ========
NUC1 1H
P1 10.40 usec
PL1 2.00 dB
SFO1 300.1518535 MHz

F2 - Processing parameters
SI 32768
SF 300.1500045 MHz
WDW EM
SSB 0
LB 0.30 Hz
GB 0
PC 10.00

30 C

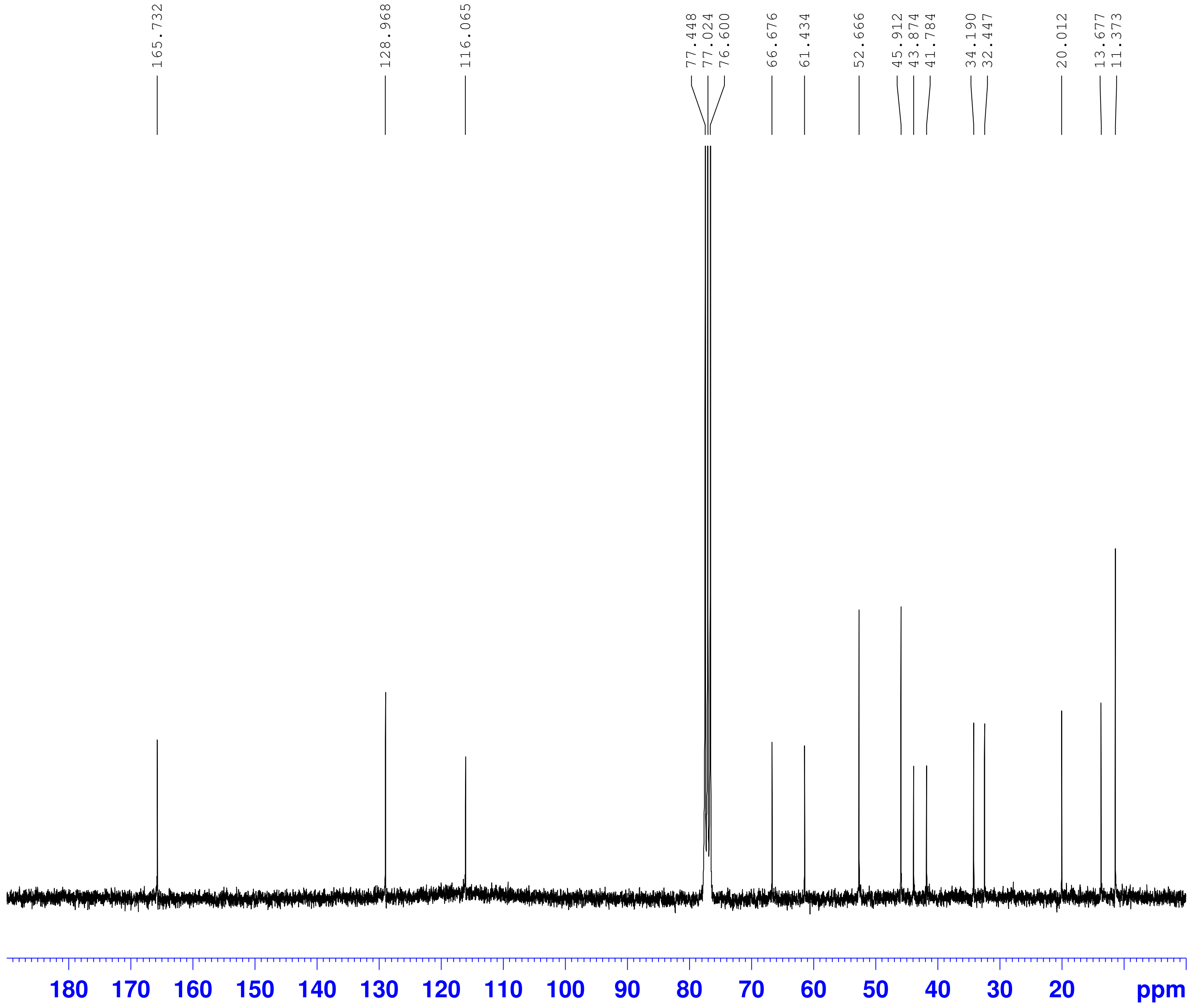


```
Current Data Parameters
NAME       Redzicka 1112
EXPNO                  2
PROCNO                 1

F2 - Acquisition Parameters
Date_           20210824
Time               21.25
INSTRUM            spect
PROBHD    5 mm BBI 1H/D-
PULPROG          zgpg30
TD                 65536
SOLVENT            CDCl3
NS                  5120
DS                     2
SWH            17985.611 Hz
FIDRES          0.274439 Hz
AQ             1.8219508 sec
RG               11585.2
DW                27.800 usec
DE                 20.00 usec
TE                 299.5 K
D1            2.00000000 sec
d11           0.03000000 sec
DELTA         1.89999998 sec
TD0                    1

======== CHANNEL f1 ========
NUC1                 13C
P1                 11.00 usec
PL1                -6.00 dB
SFO1          75.4803248 MHz

======== CHANNEL f2 ========
CPDPRG2          waltz16
NUC2                  1H
PCPD2             100.00 usec
PL2                 2.00 dB
PL12               21.45 dB
PL13               23.00 dB
SFO2         300.1512006 MHz

F2 - Processing parameters
SI                 32768
SF            75.4727780 MHz
WDW                   EM
SSB                    0
LB                  1.00 Hz
GB                     0
PC                  2.00
```

3o H

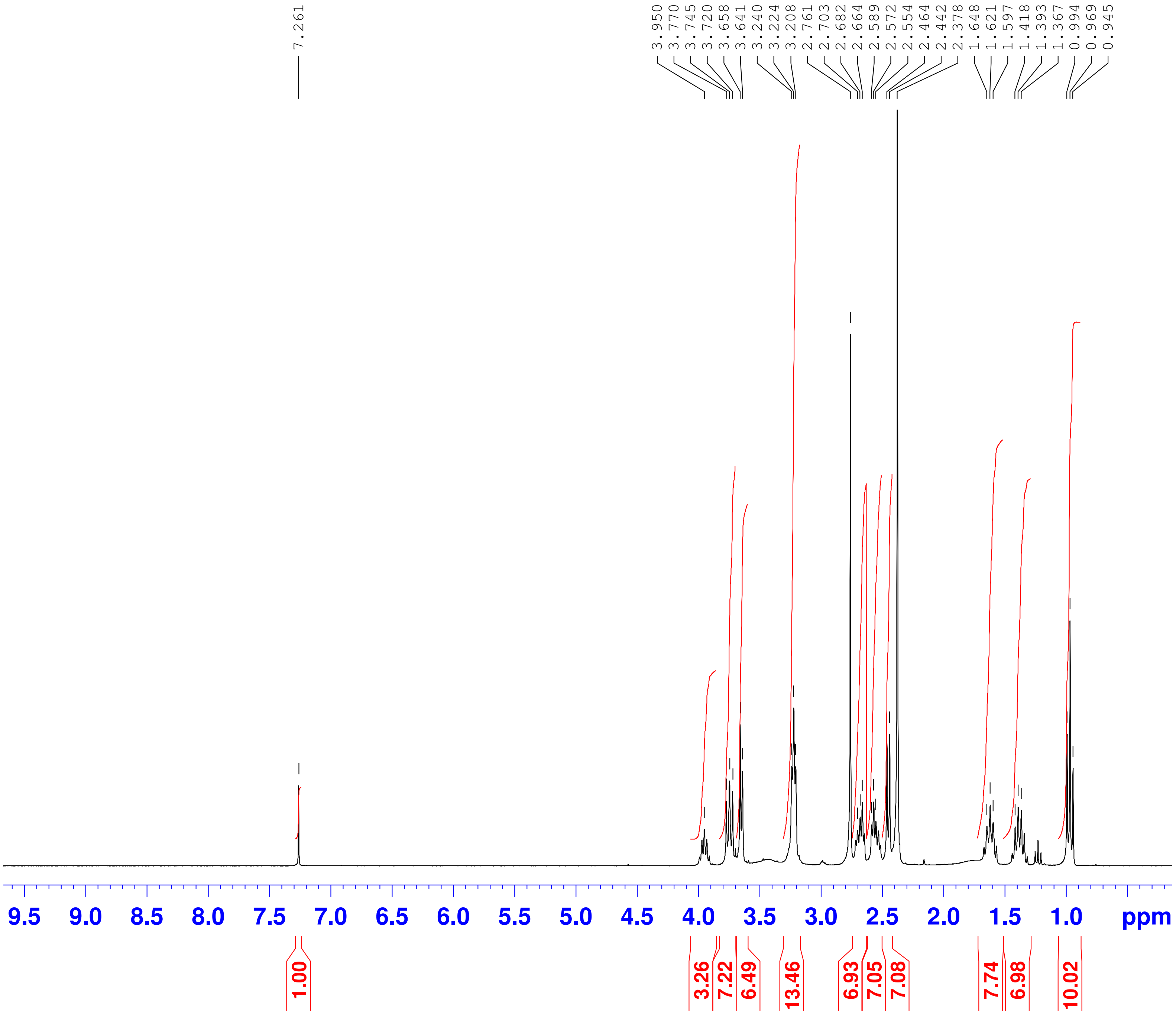

Current Data Parameters	
NAME	Redzicka 1112
EXPNO	1
PROCNO	1

F2 - Acquisition Parameters	
Date_	20210825
Time	8.31
INSTRUM	spect
PROBHD	5 mm BBI 1H/D-
PULPROG	zg30
TD	65536
SOLVENT	CDCl3
NS	16
DS	0
SWH	6172.839 Hz
FIDRES	0.094190 Hz
AQ	5.3084660 sec
RG	80.6
DW	81.000 usec
DE	8.00 usec
TE	298.5 K
D1	1.00000000 sec
TD0	1

======== CHANNEL f1 ========	
NUC1	1H
P1	10.40 usec
PL1	2.00 dB
SFO1	300.1518535 MHz

F2 - Processing parameters	
SI	32768
SF	300.1500045 MHz
WDW	EM
SSB	0
LB	0.30 Hz
GB	0
PC	0.50

2c C

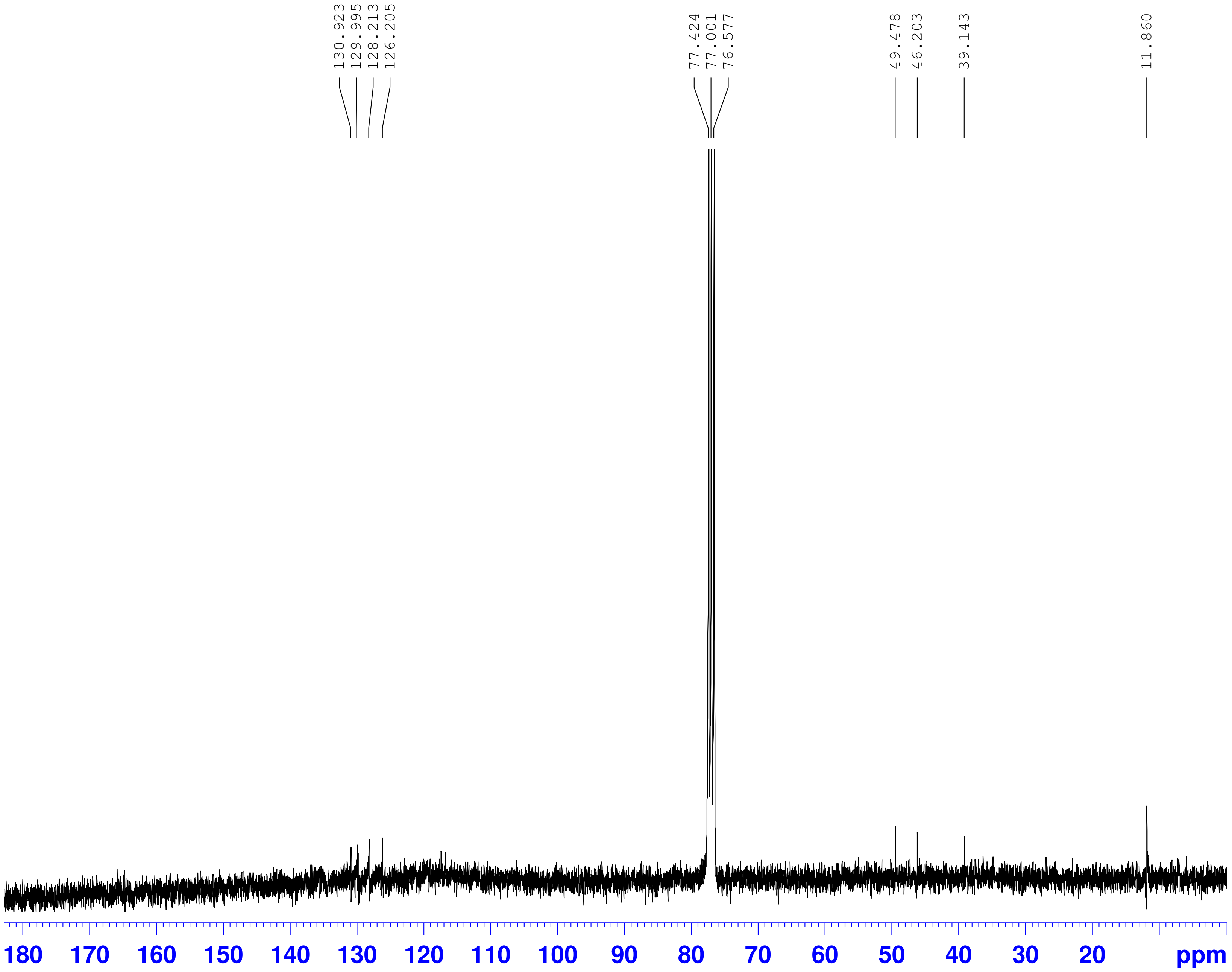

Current Data Parameters
NAME Redzicka 1100
EXPNO 2
PROCNO 1

F2 - Acquisition Parameters

Date_	20210817	
Time	20.38	
INSTRUM	spect	
PROBHD	5 mm BBI 1H/D-	
PULPROG	zgpg30	
TD	65536	
SOLVENT	CDCl3	
NS	5120	
DS	2	
SWH	17985.611	Hz
FIDRES	0.274439	Hz
AQ	1.8219508	sec
RG	26008	
DW	27.800	usec
DE	20.00	usec
TE	299.4	K
D1	2.00000000	sec
d11	0.03000000	sec
DELTA	1.89999998	sec
TD0	1	

======== CHANNEL f1 ========

NUC1	13C	
P1	11.00	usec
PL1	-6.00	dB
SFO1	75.4803248	MHz

======== CHANNEL f2 ========

CPDPRG2	waltz16	
NUC2	1H	
PCPD2	100.00	usec
PL2	2.00	dB
PL12	21.45	dB
PL13	23.00	dB
SFO2	300.1512006	MHz

F2 - Processing parameters

SI	32768	
SF	75.4727780	MHz
WDW	EM	
SSB	0	
LB	1.00	Hz
GB	0	
PC	2.00	

2c H

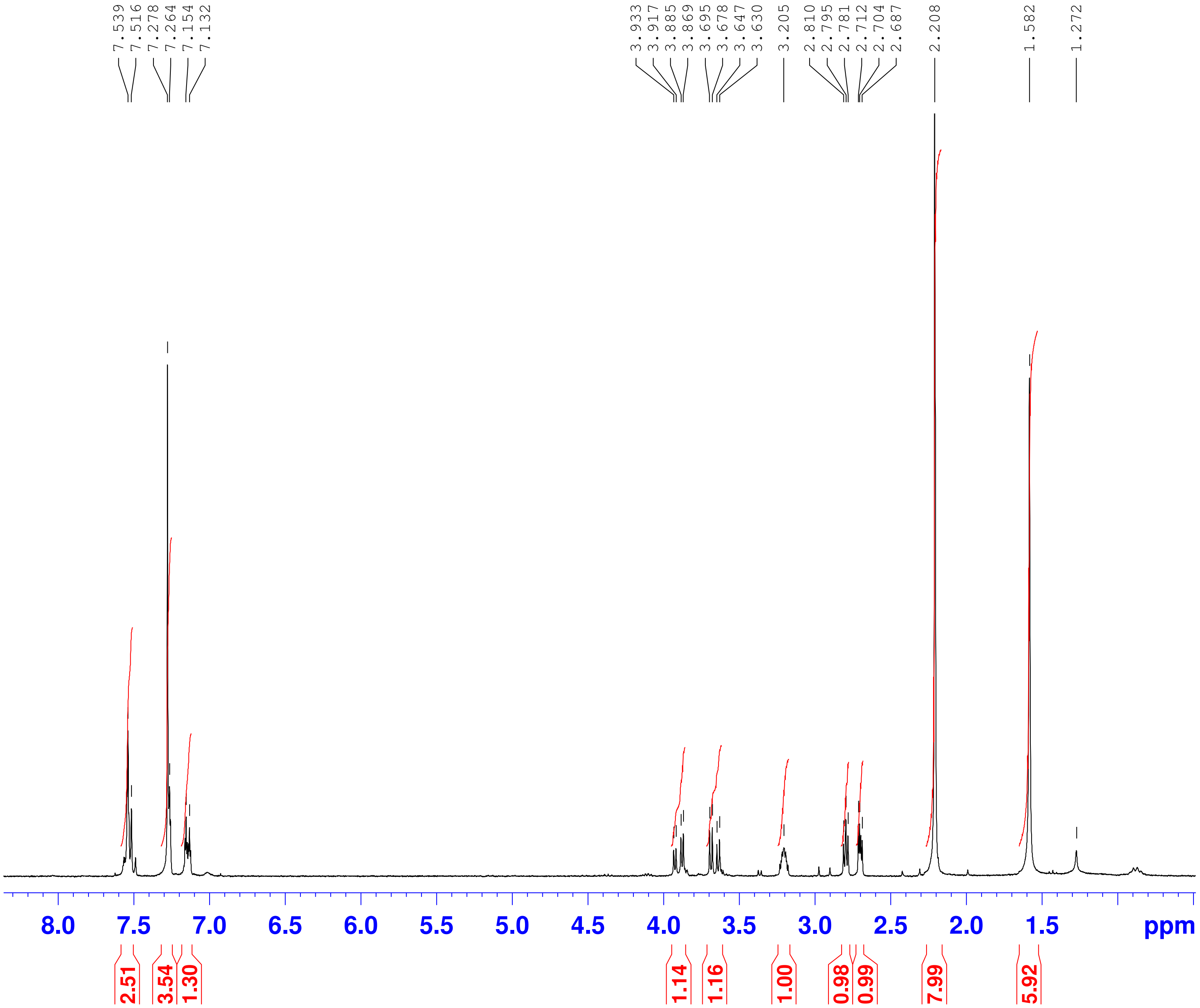


```
Current Data Parameters
NAME         Redzicka 1100
EXPNO                    1
PROCNO                   1

F2 - Acquisition Parameters
Date_             20210817
Time                 15.05
INSTRUM              spect
PROBHD    5 mm BBI 1H/D-
PULPROG               zg30
TD                   65536
SOLVENT              CDCl3
NS                      16
DS                       0
SWH               6172.839 Hz
FIDRES            0.094190 Hz
AQ               5.3084660 sec
RG                   812.7
DW                  81.000 usec
DE                    8.00 usec
TE                   298.1 K
D1              1.00000000 sec
TD0                      1

======== CHANNEL f1 ========
NUC1                    1H
P1                   11.00 usec
PL1                   2.50 dB
SFO1           300.1518535 MHz

F2 - Processing parameters
SI                   32768
SF             300.1500000 MHz
WDW                     EM
SSB                      0
LB                    0.30 Hz
GB                       0
PC                   20.00
```

3 a

Window Display Report

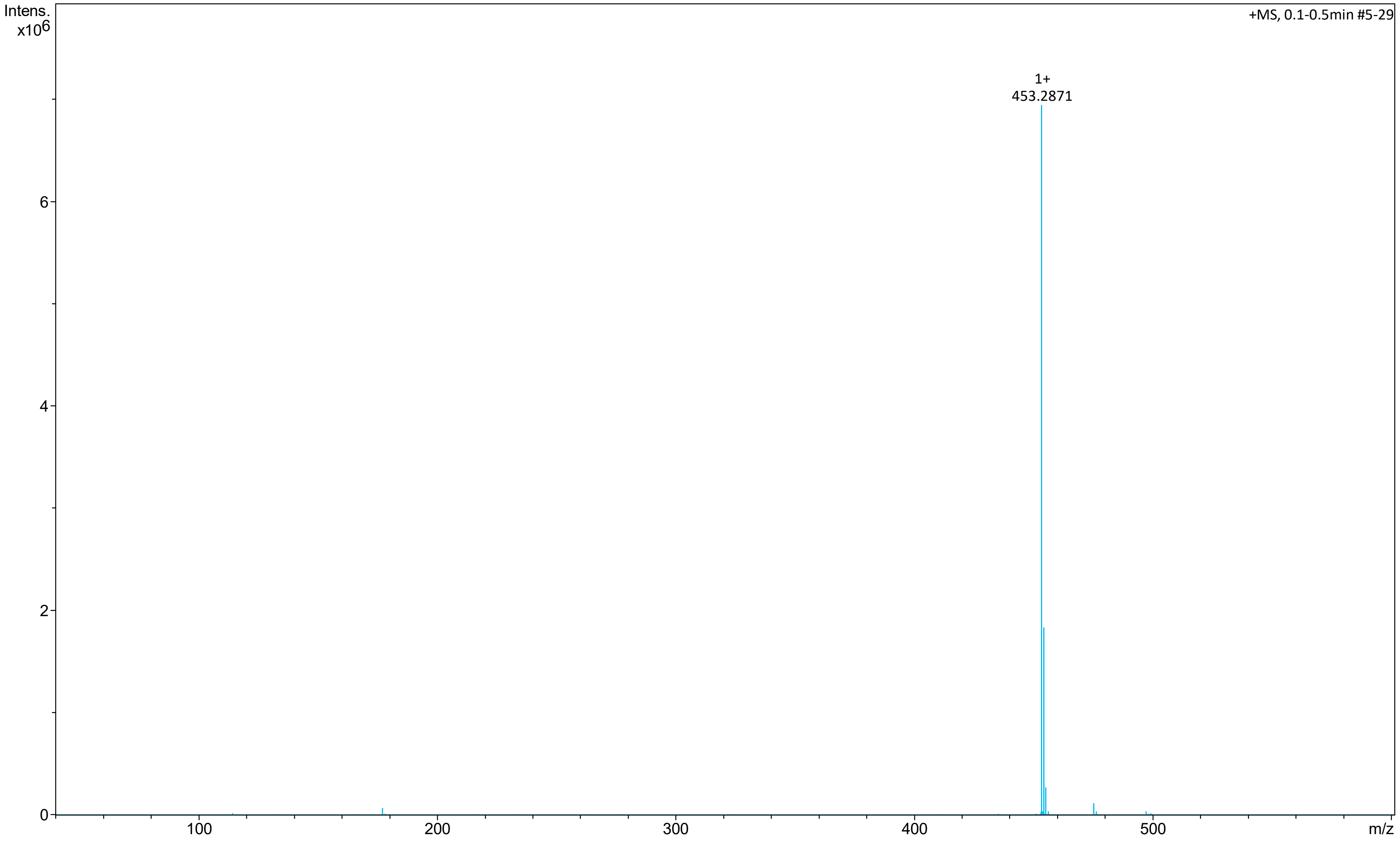

3 b

Window Display Report

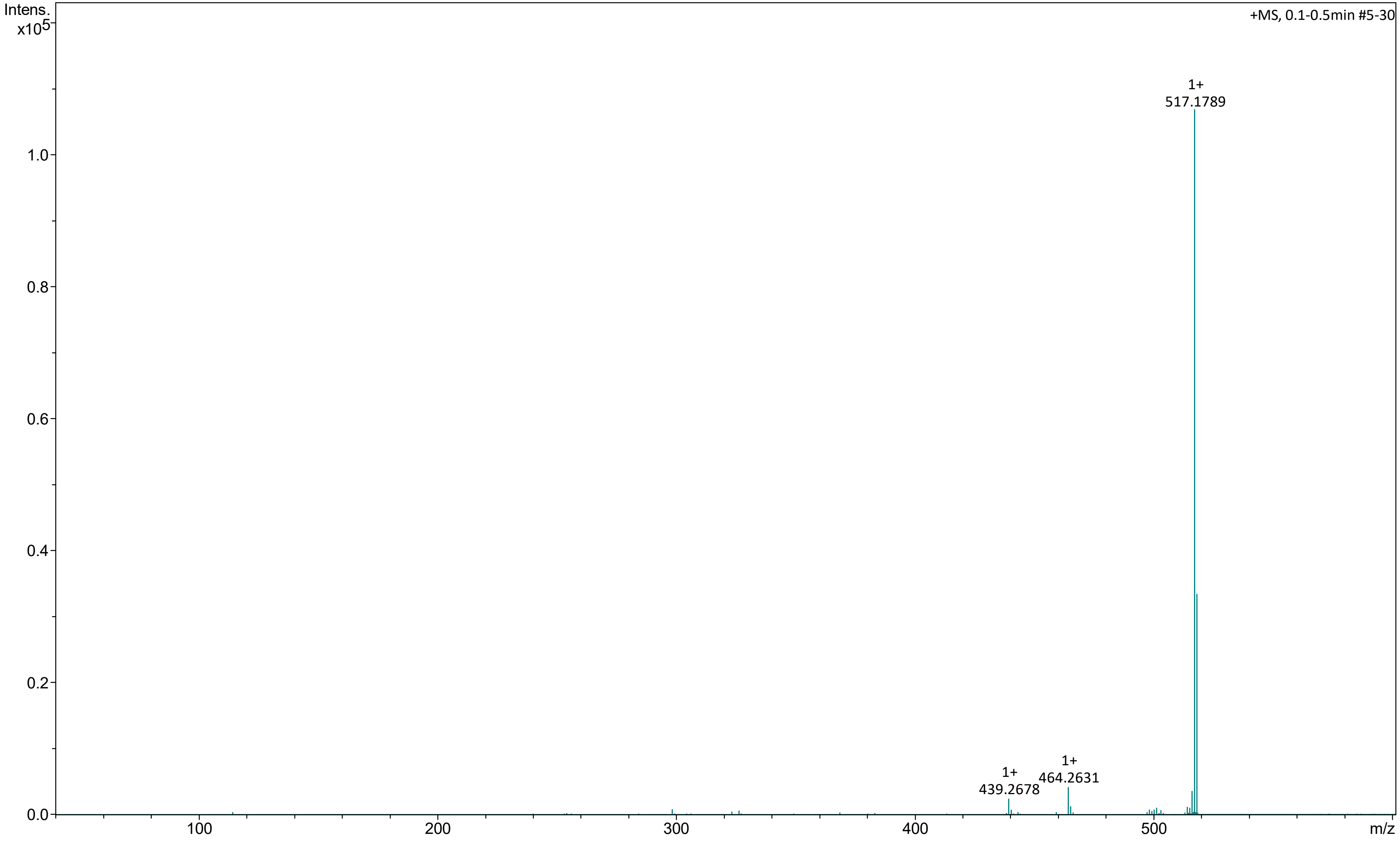

3 c

Window Display Report

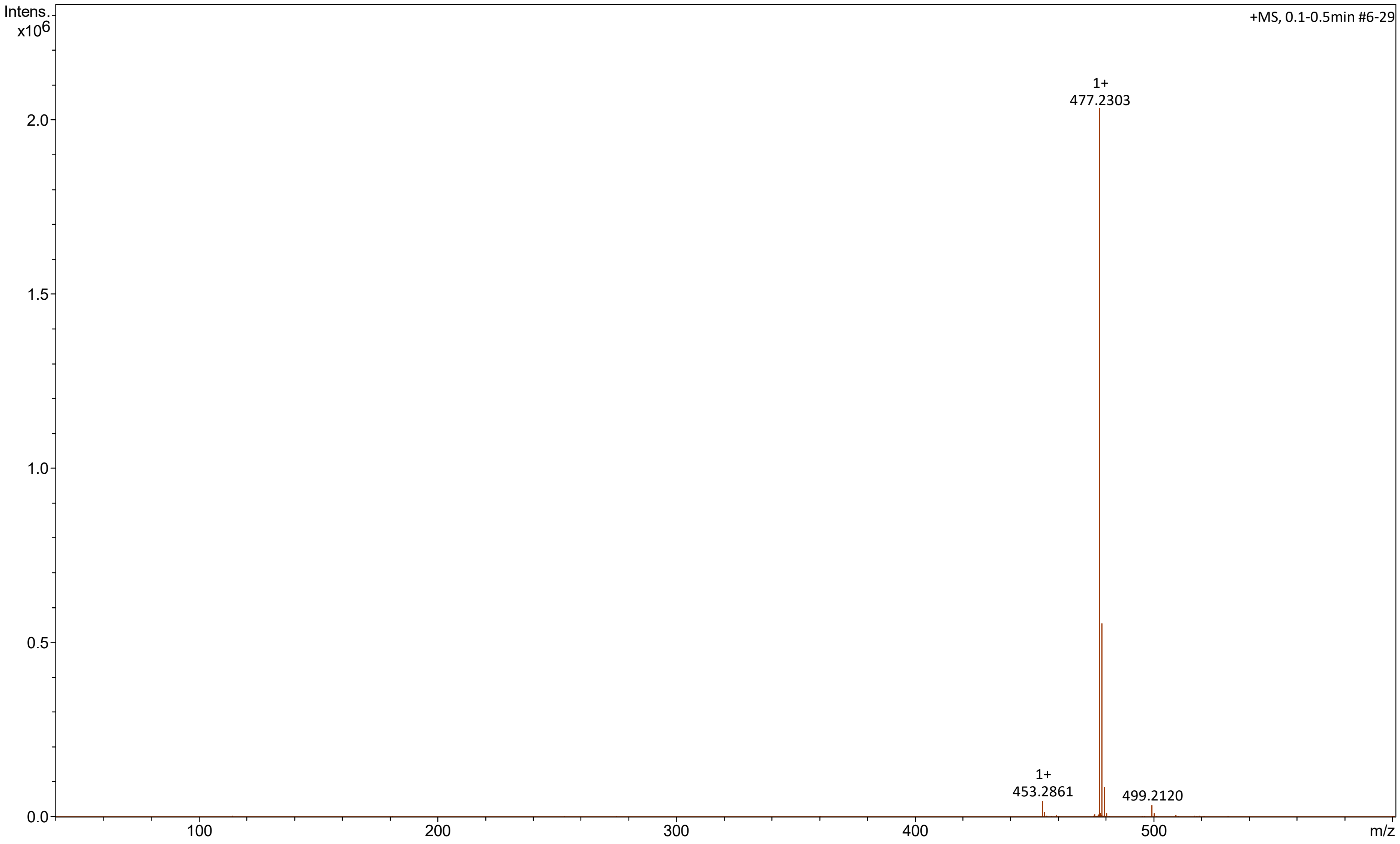

3 d

Window Display Report

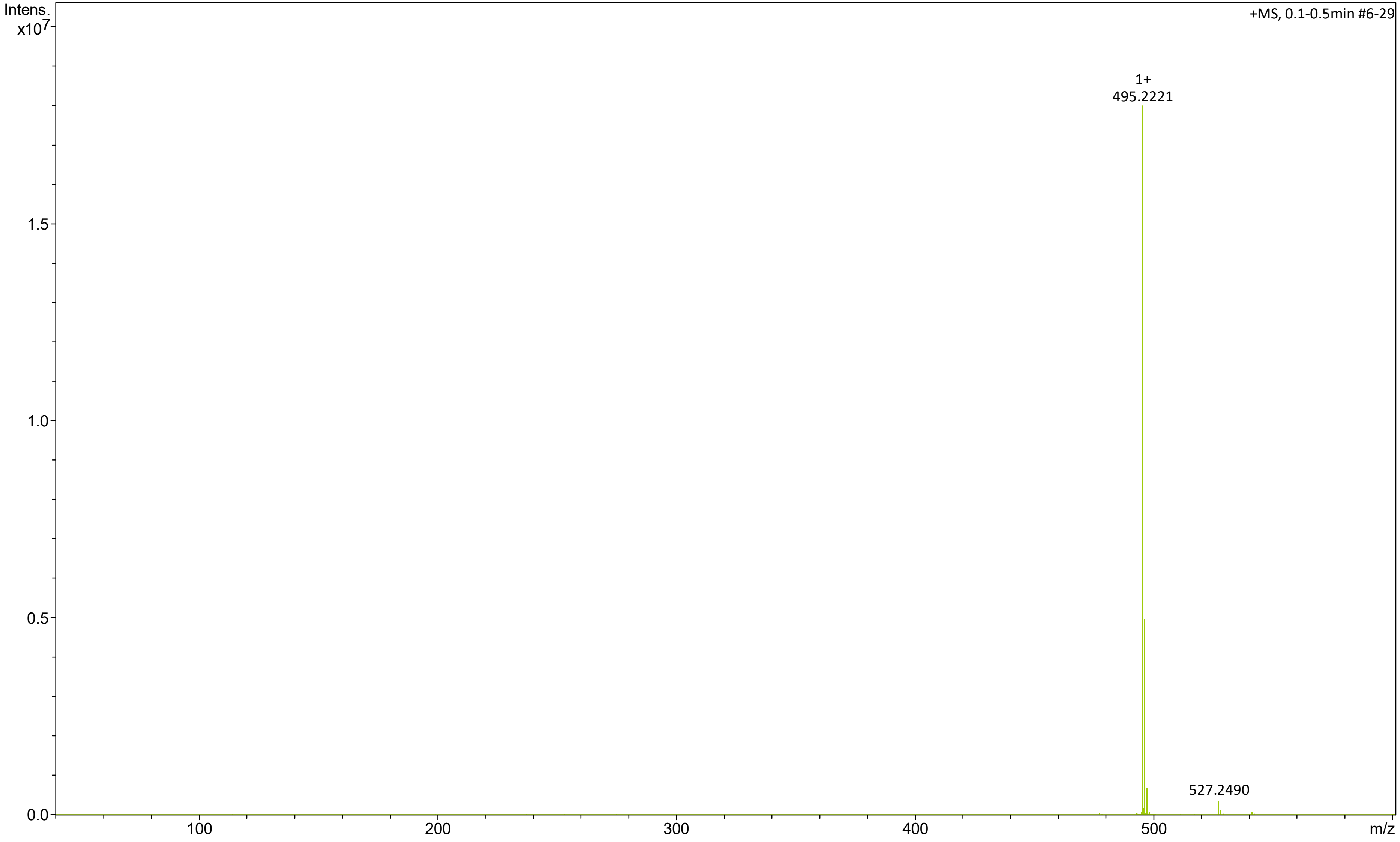

3 e

Window Display Report

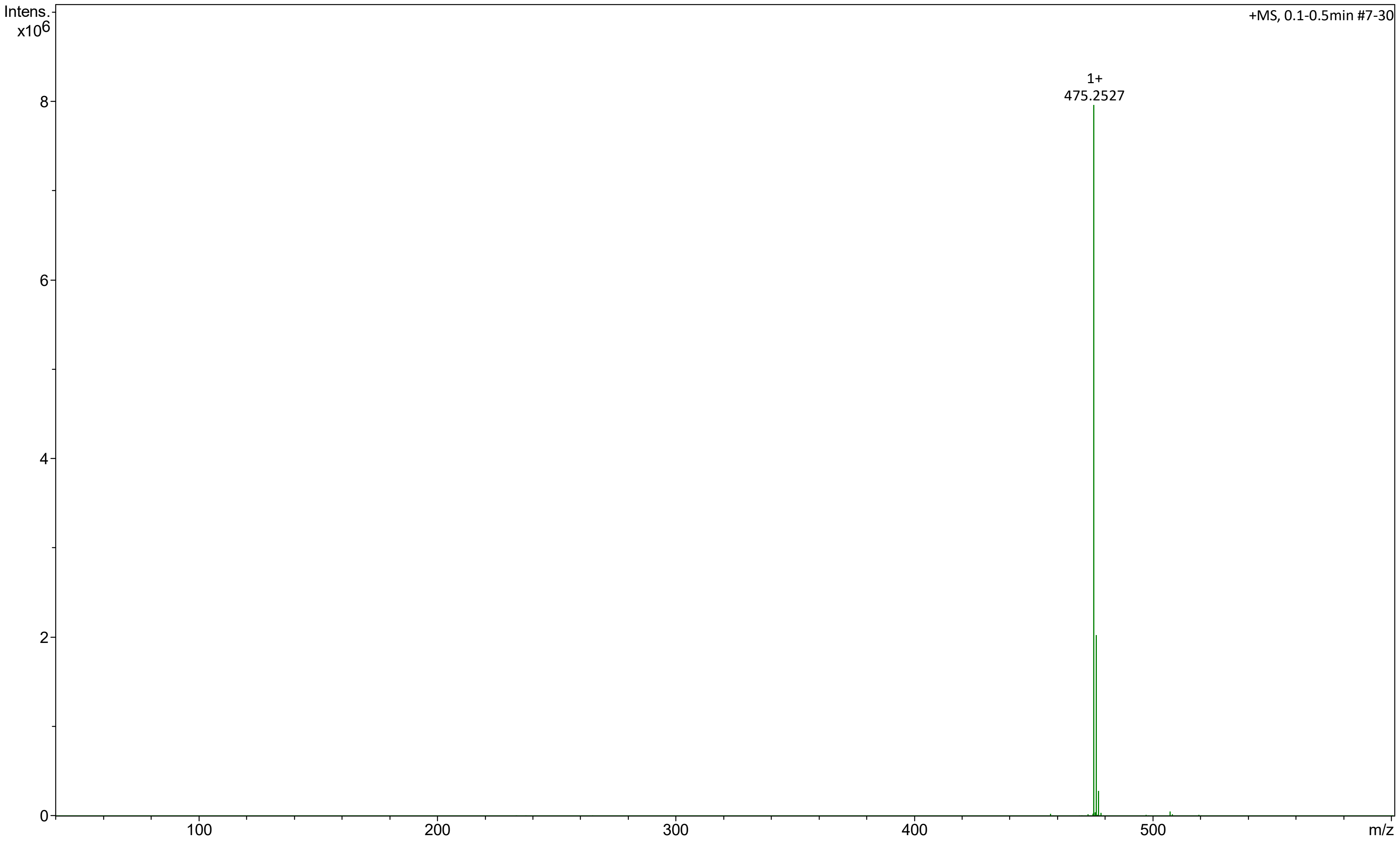

3 f

Window Display Report

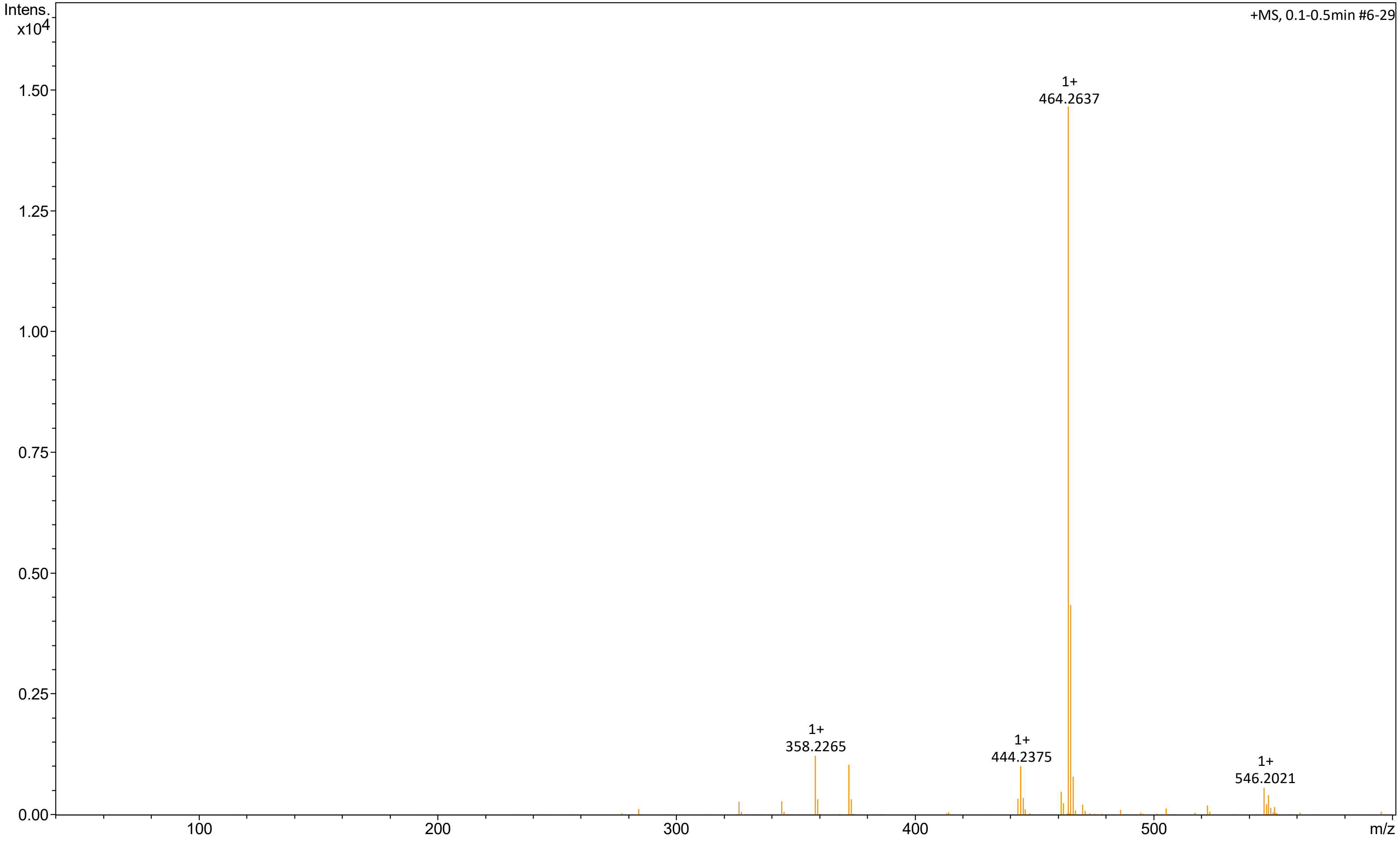

3 g

Window Display Report

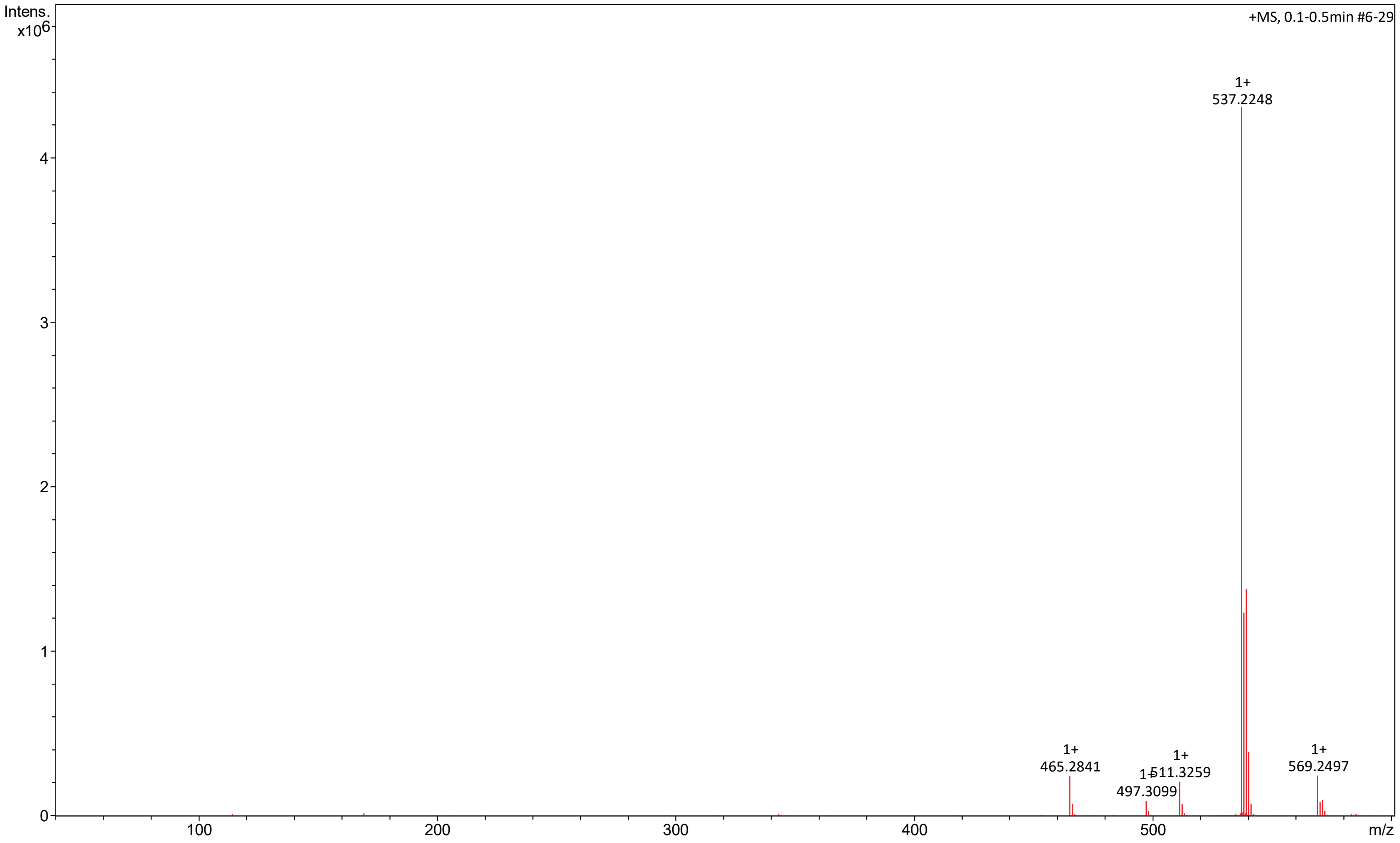

3 h

Window Display Report

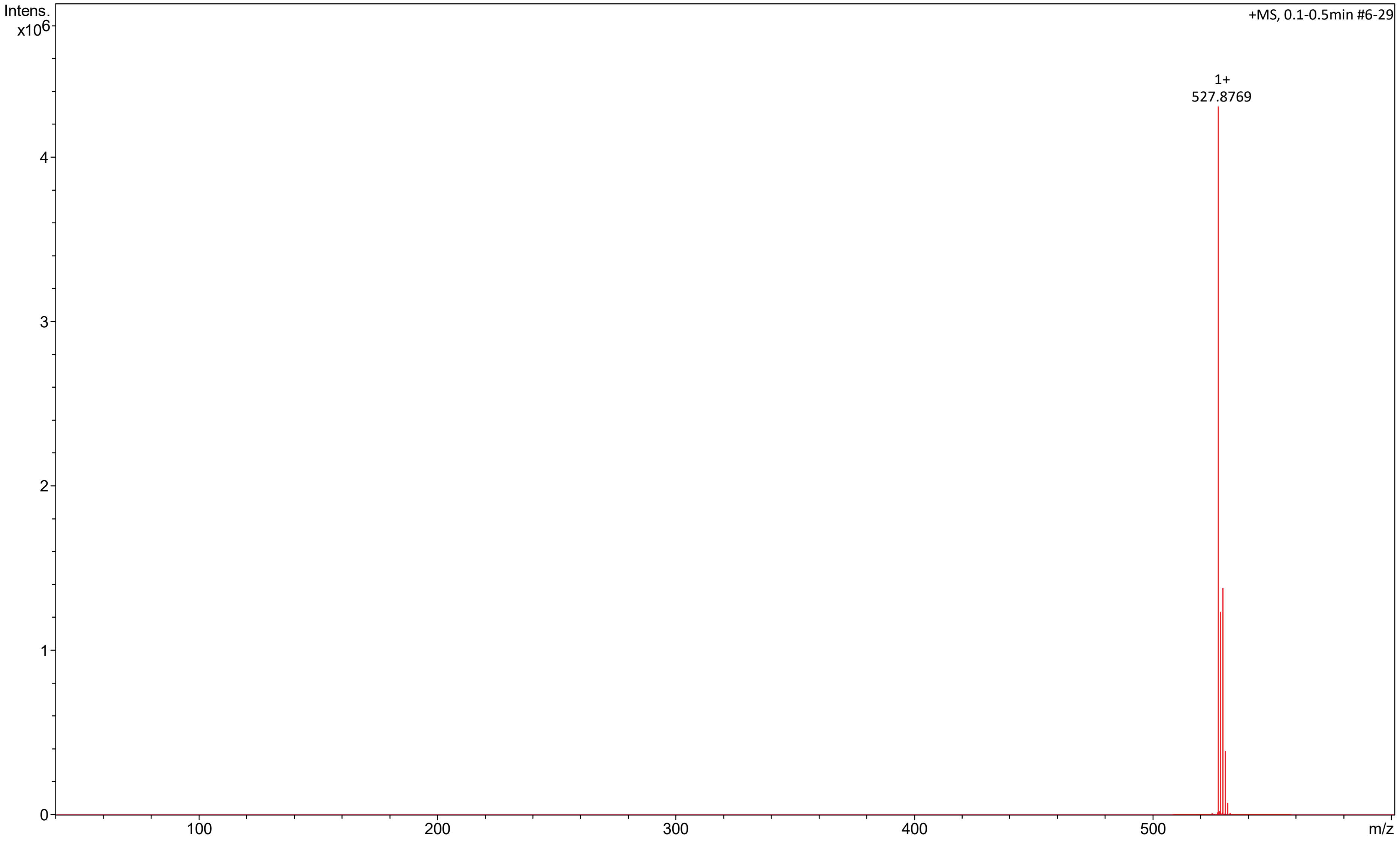

3 i

Window Display Report

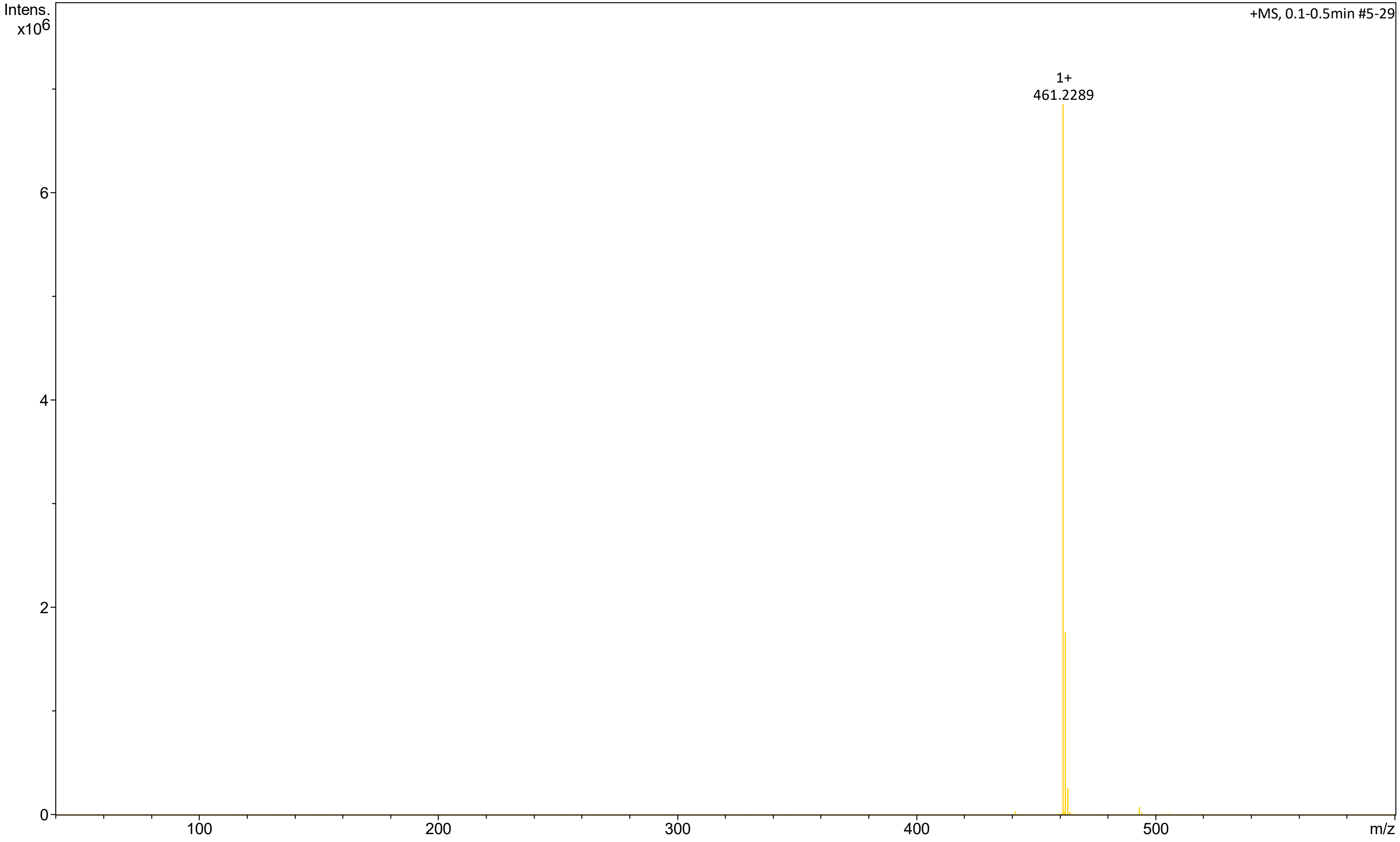

3 j

Window Display Report

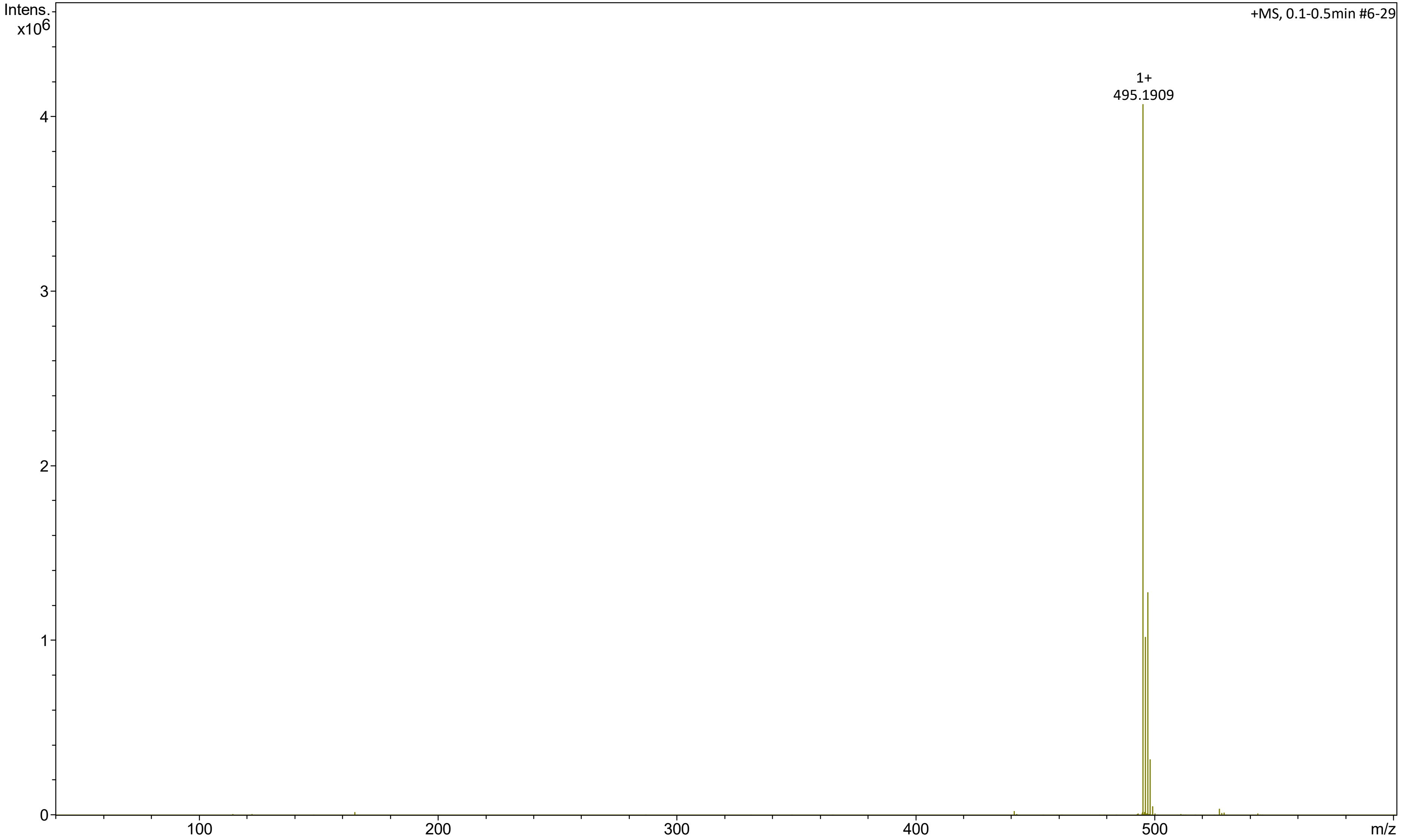

3 k

Window Display Report

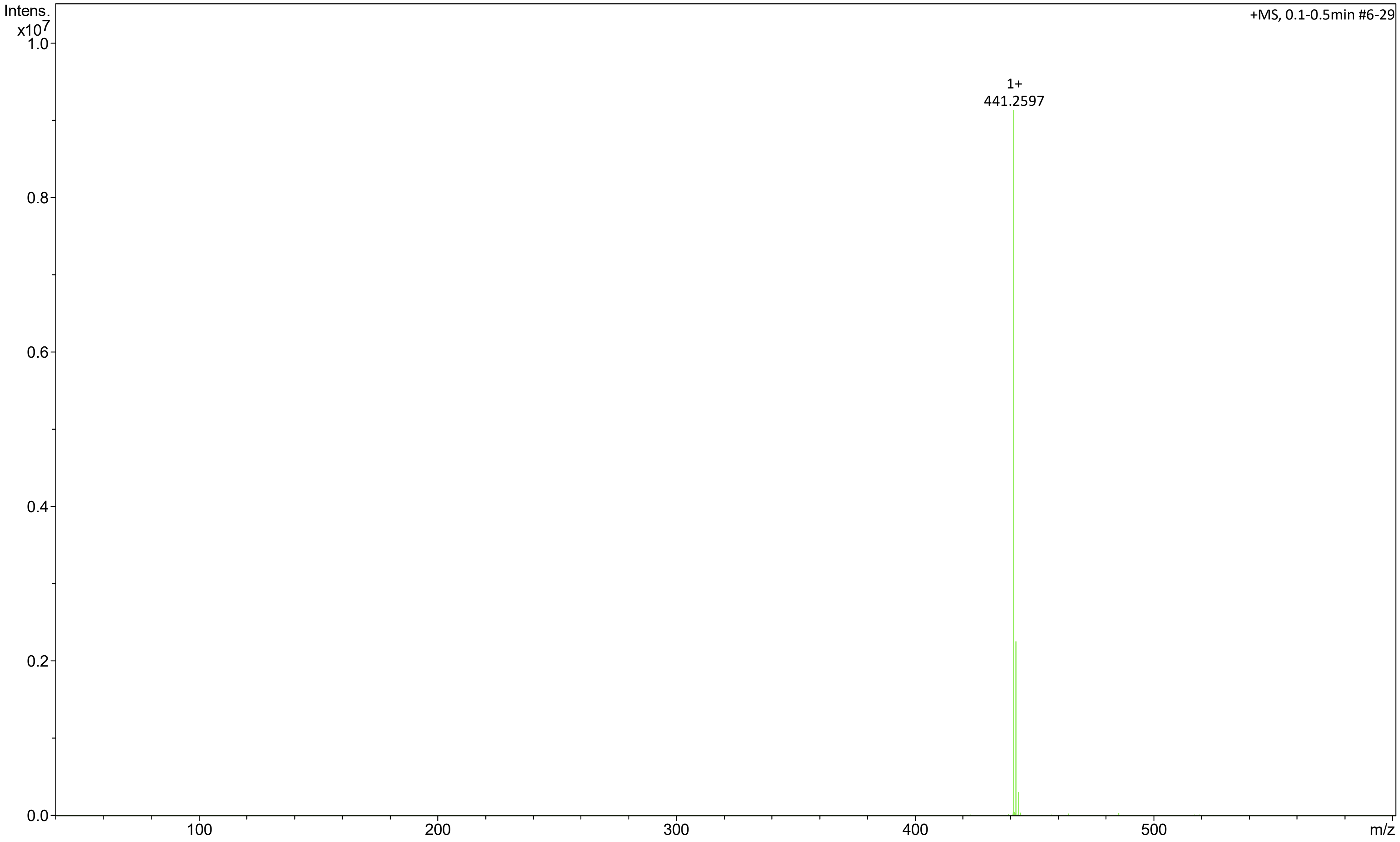

3 l

Window Display Report

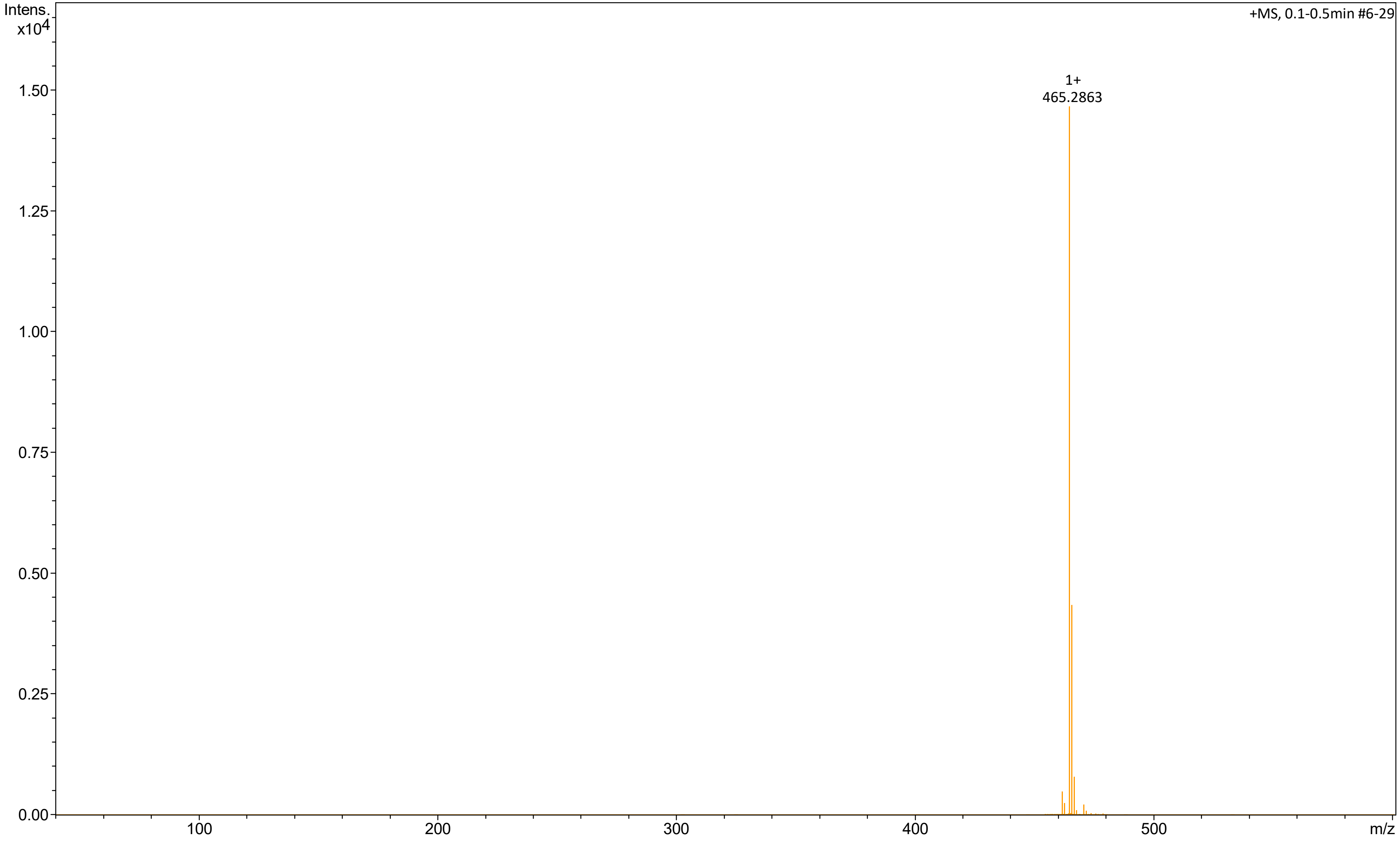

3 m

Window Display Report

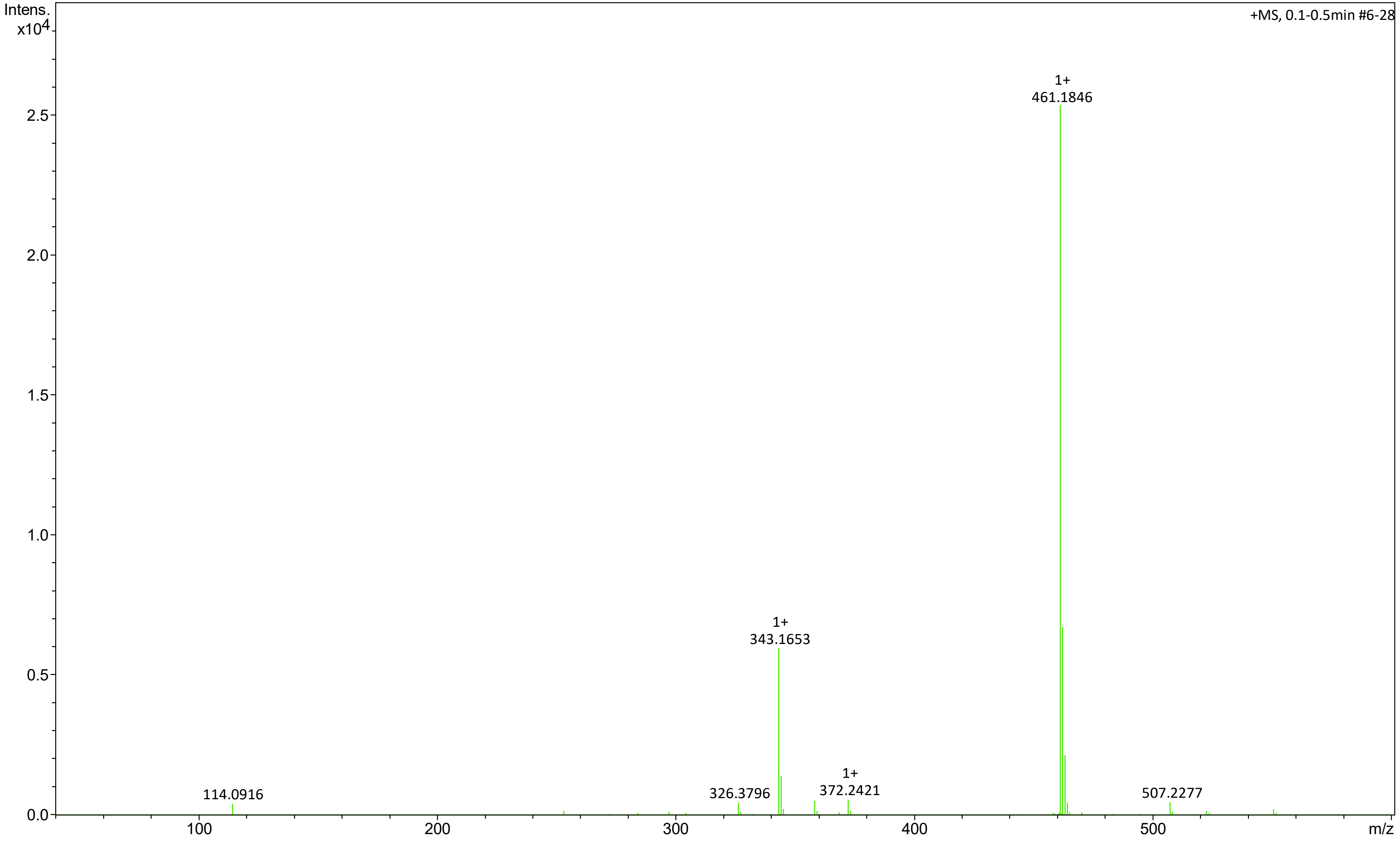

3 n

Window Display Report

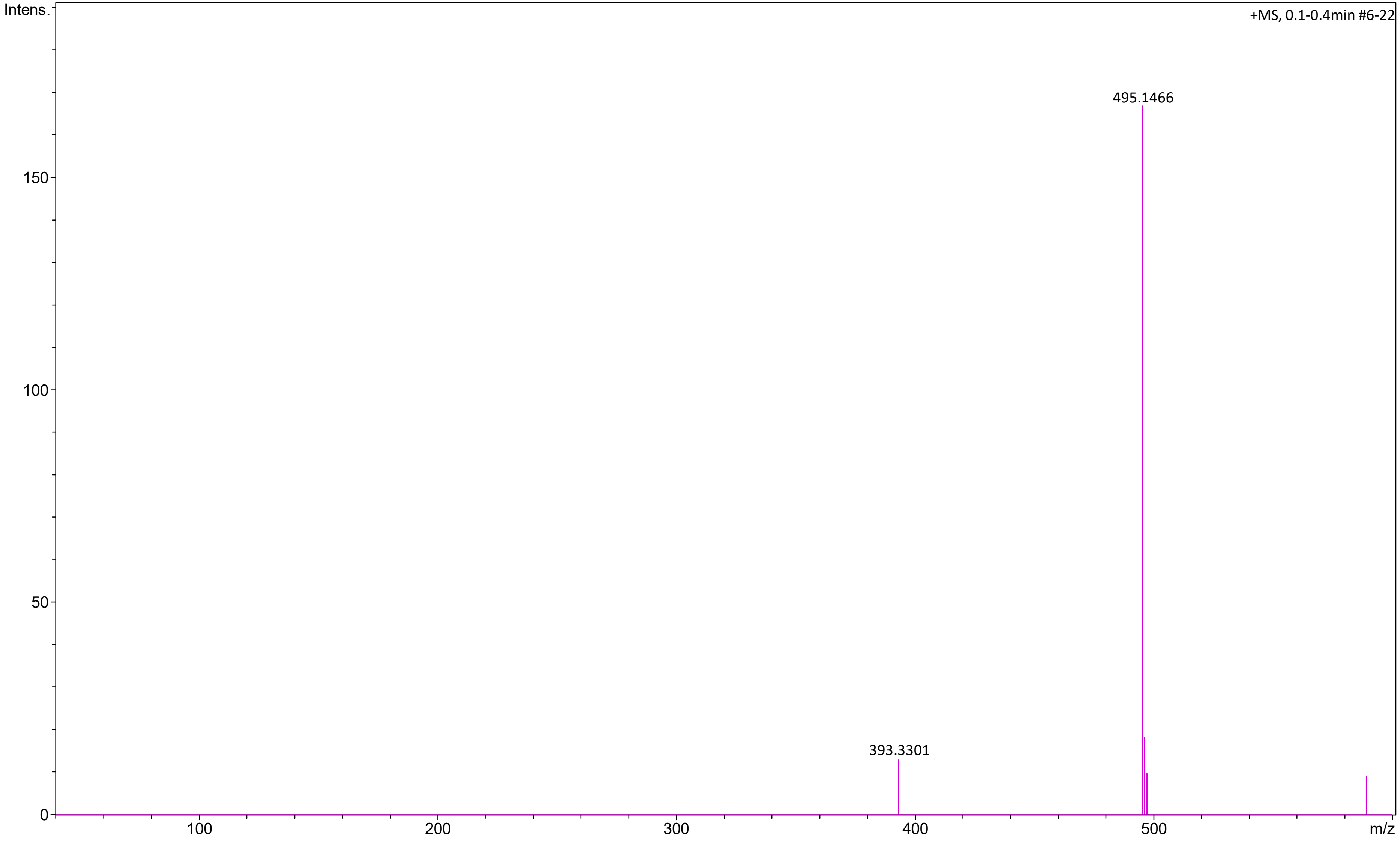

3 o

Window Display Report

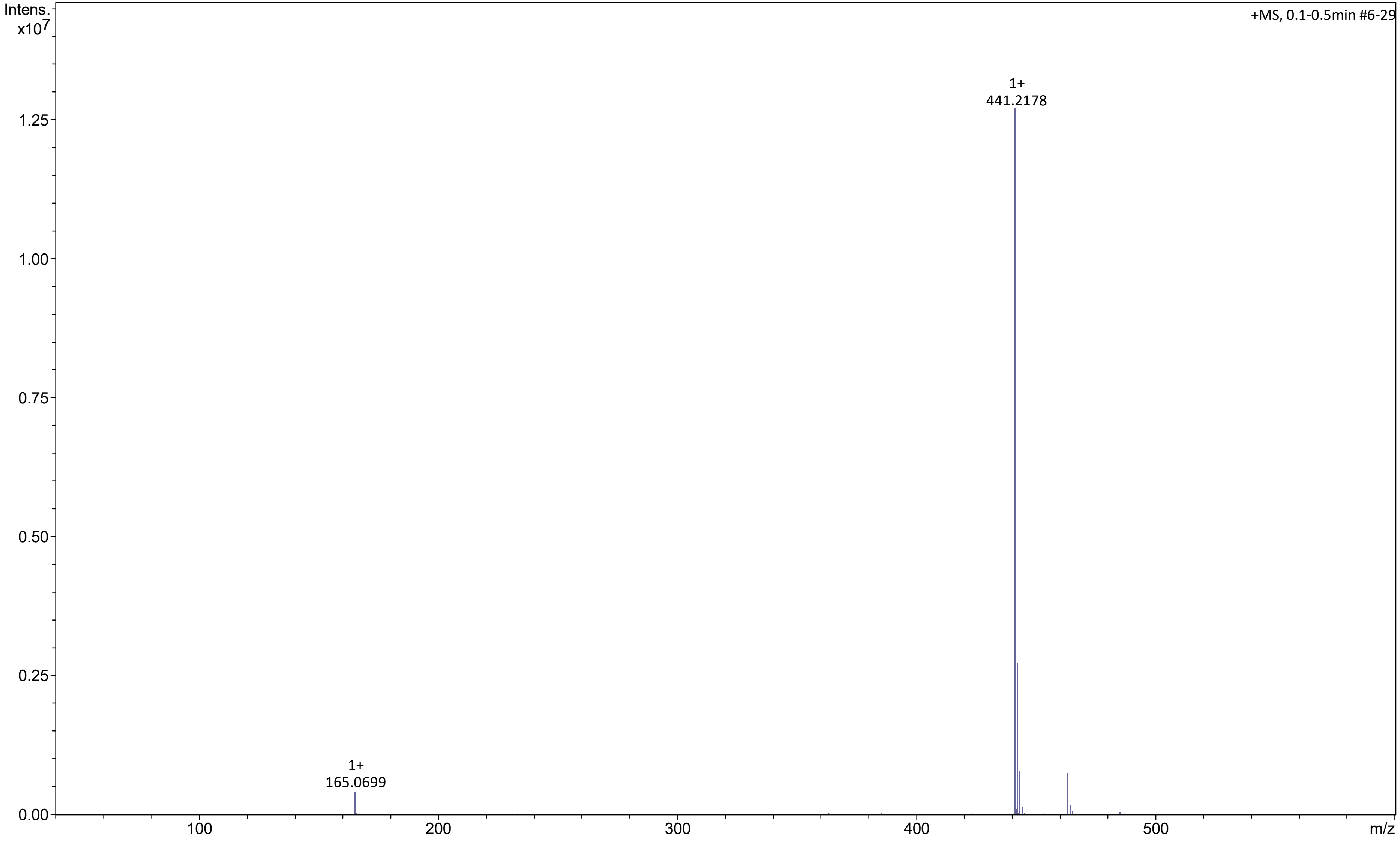

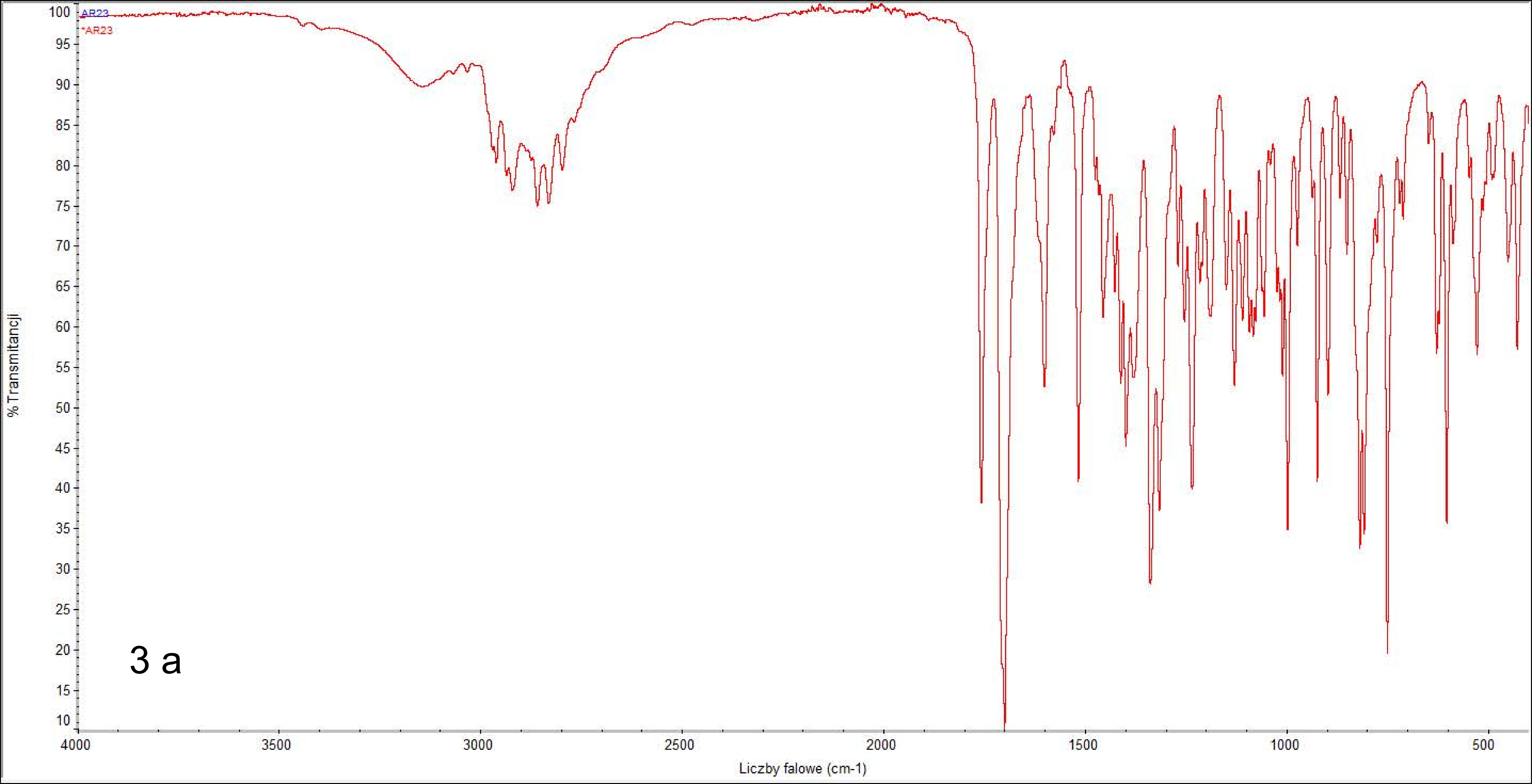

AR23
*AR23
%Transmitancji
Liczby falowe (cm-1)
3 a

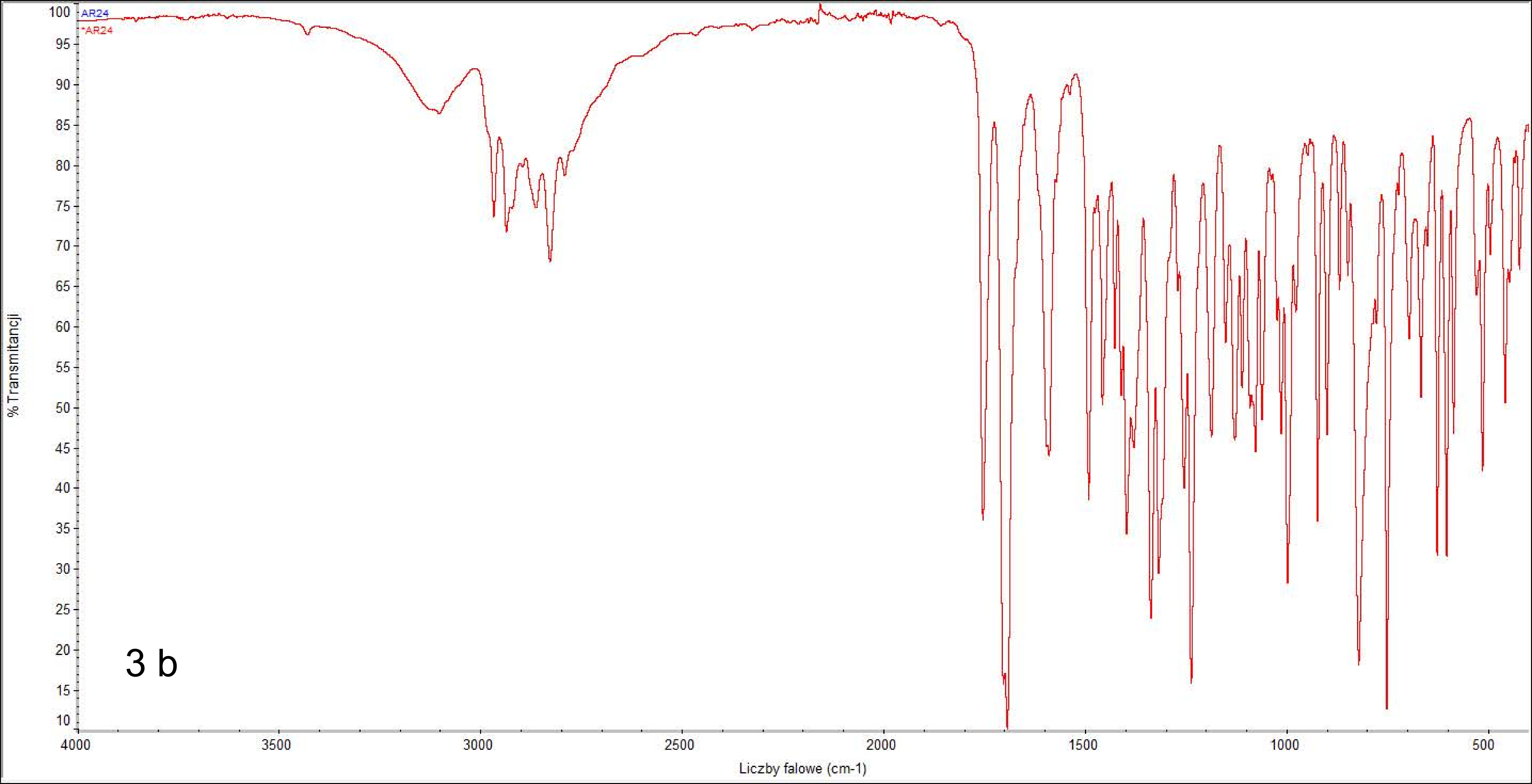

AR24
*AR24
%Transmitancji
Liczby falowe (cm-1)
3 b

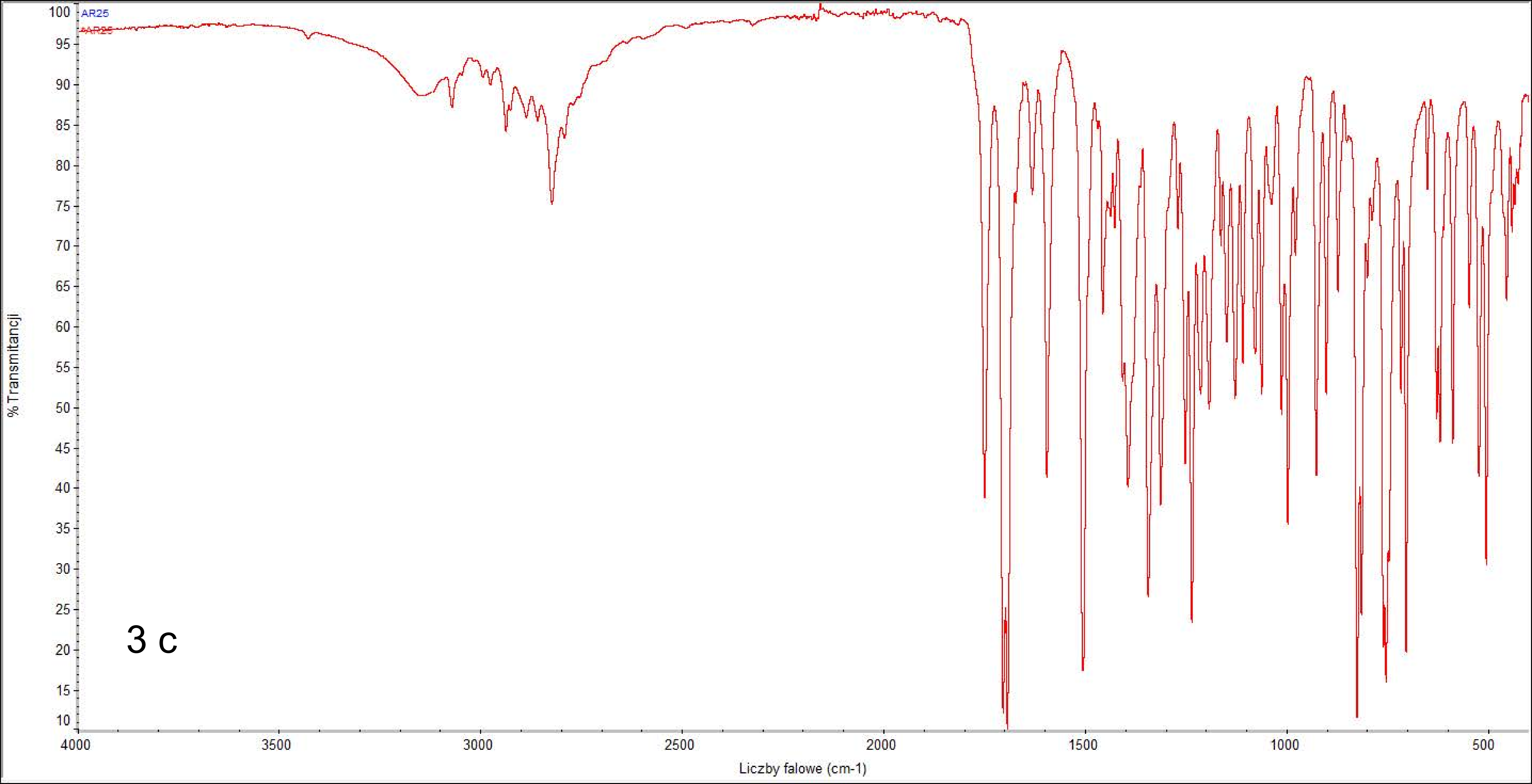
AR25
AR25
% Transmitancji
Liczby falowe (cm-1)
3 c

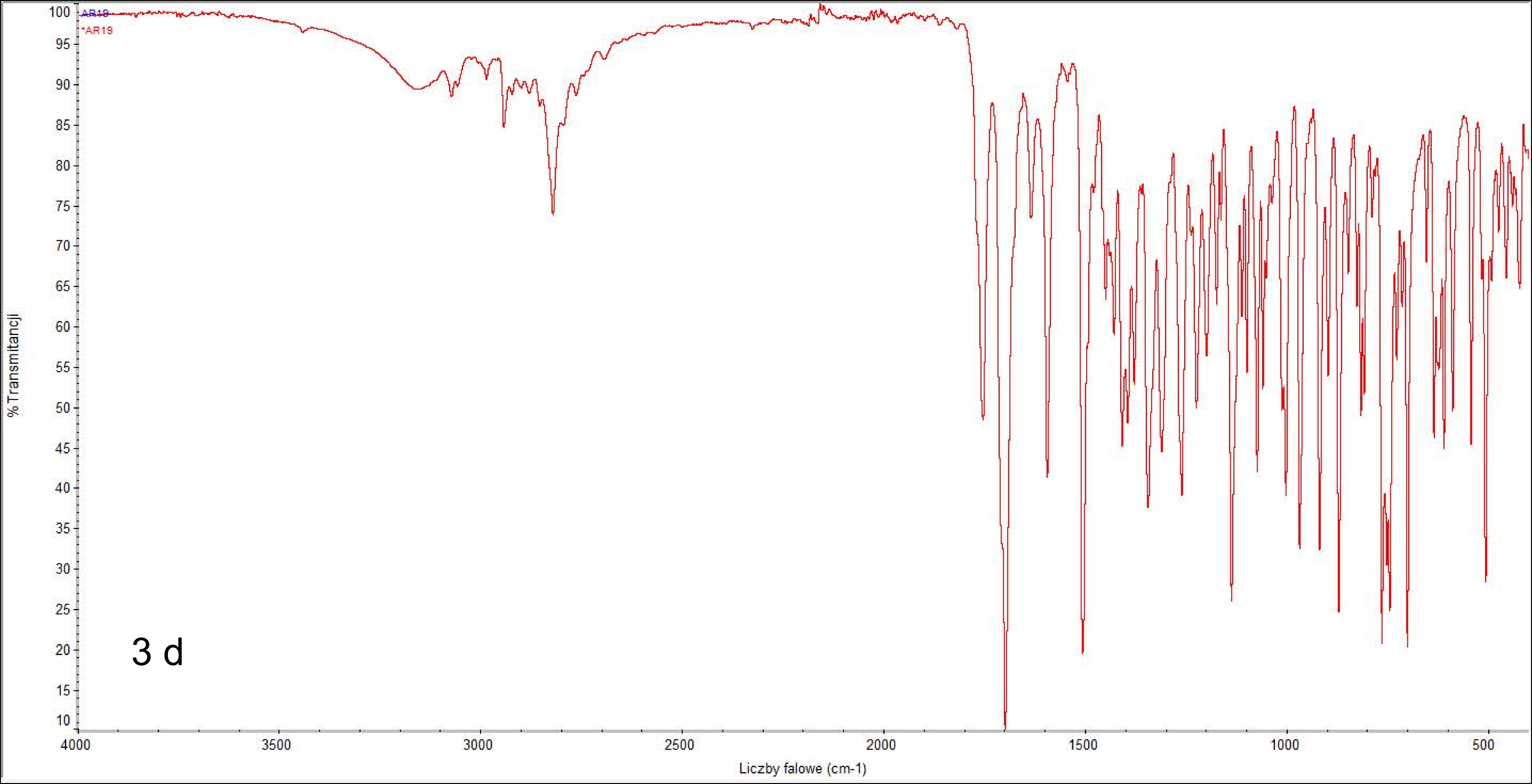

AR19
*AR19
%Transmitancji
Liczby falowe (cm-1)
3 d

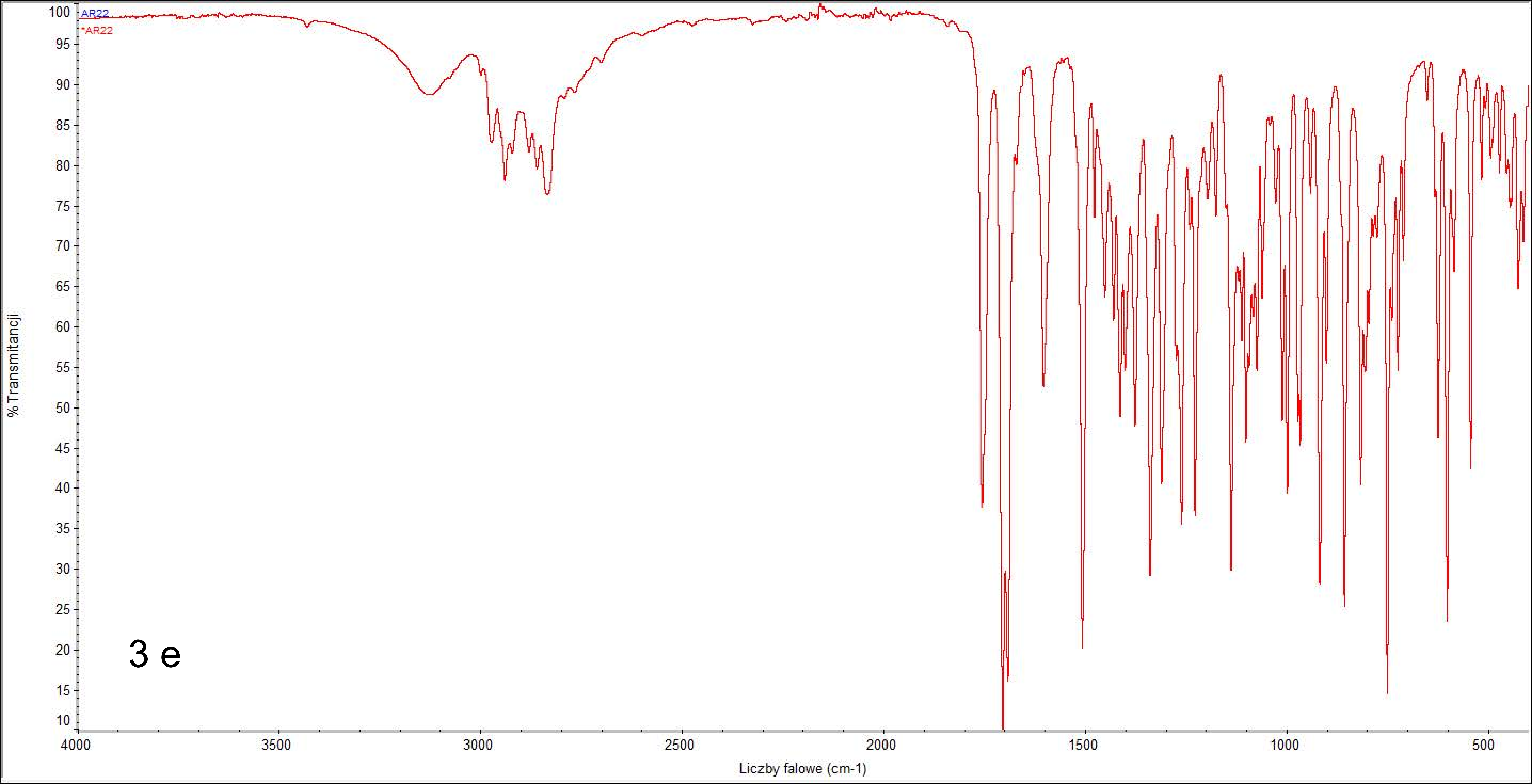
AR22
*AR22
%Transmitancji
100
95
90
85
80
75
70
65
60
55
50
45
40
35
30
25
20
15
10
4000
3500
3000
2500
2000
1500
1000
500
Liczby falowe (cm-1)
3 e

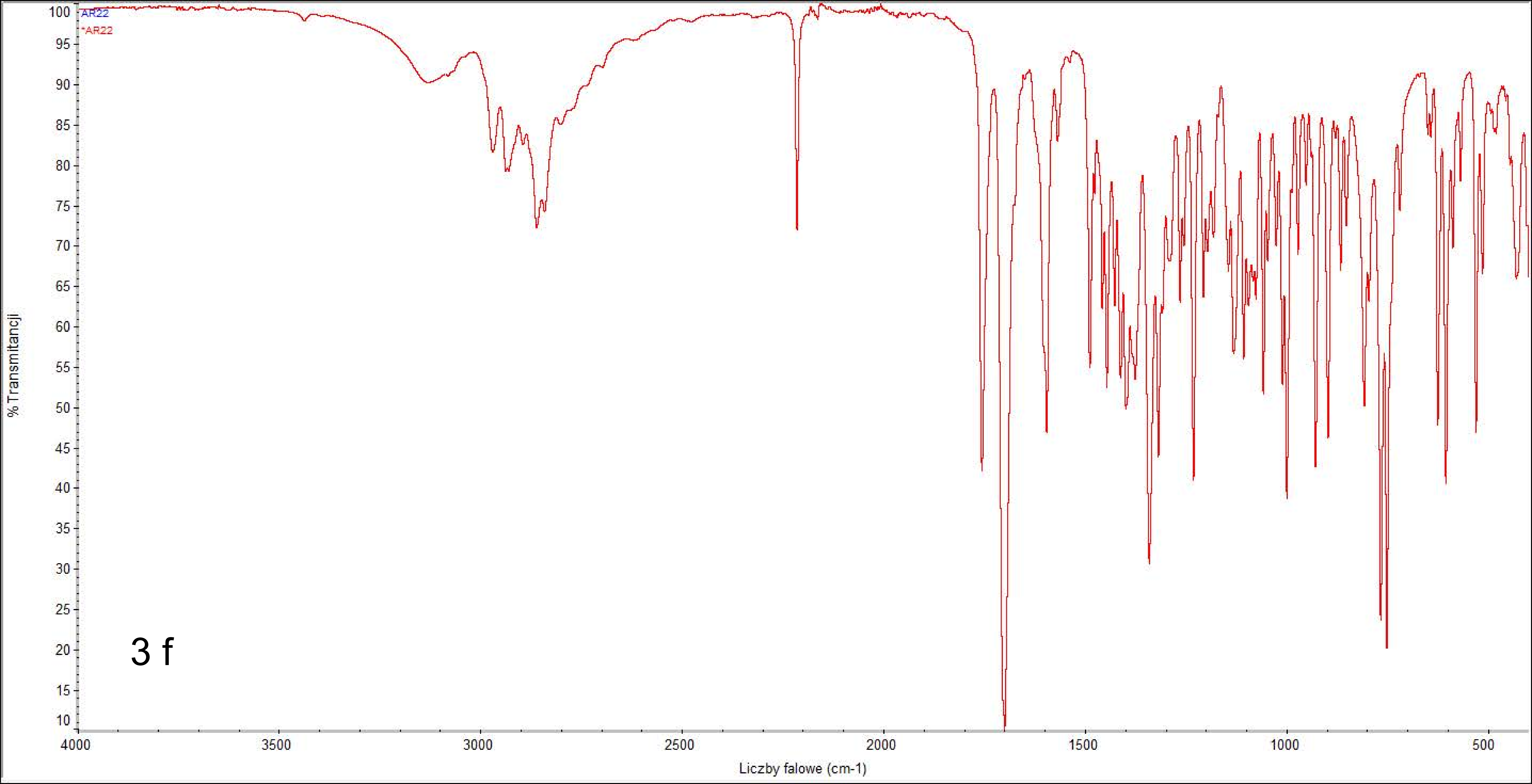

AR22
*AR22
%Transmitancji
Liczby falowe (cm-1)
3 f

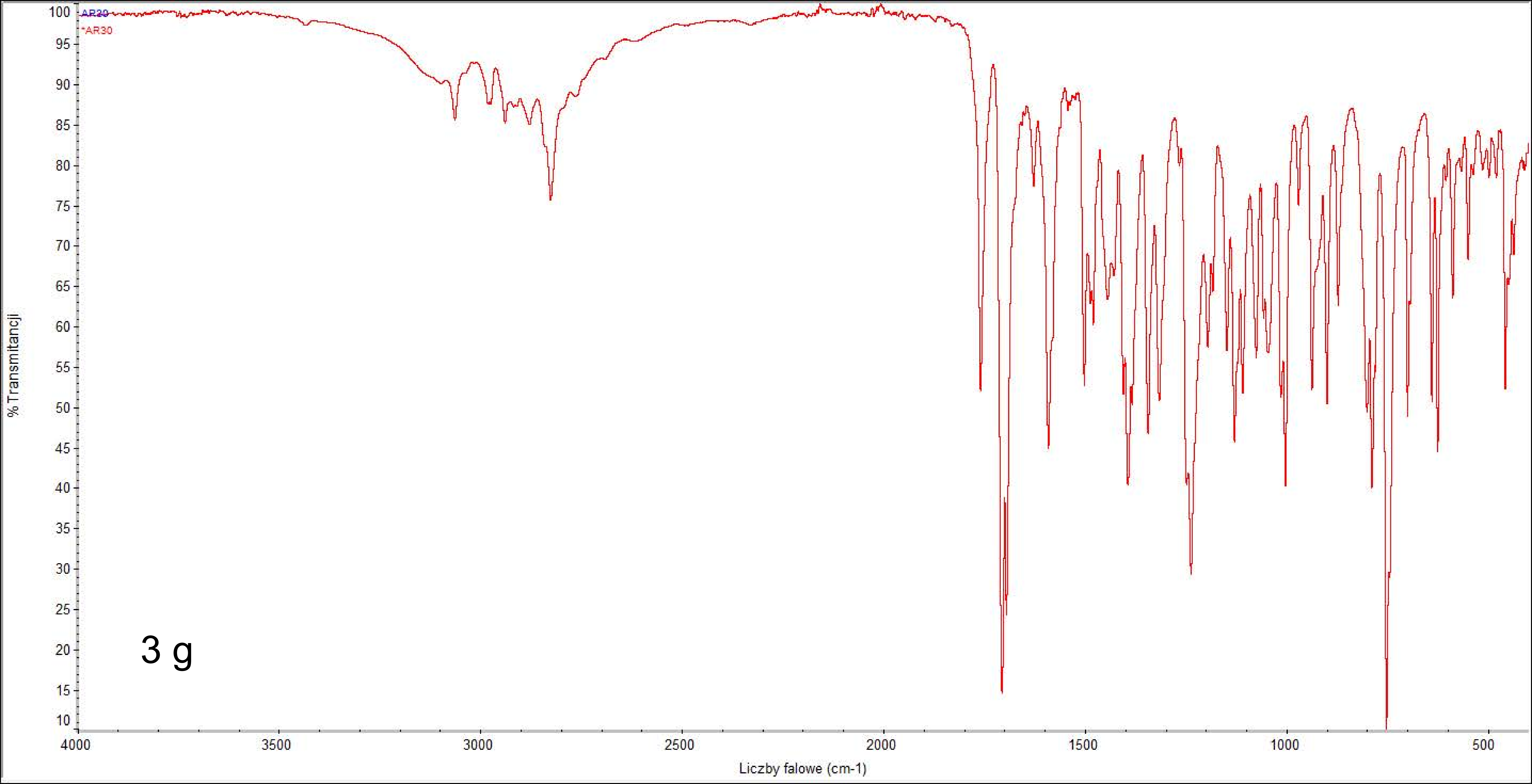
AR30
*AR30
% Transmitancji
Liczby falowe (cm-1)
3 g
100
95
90
85
80
75
70
65
60
55
50
45
40
35
30
25
20
15
10
4000
3500
3000
2500
2000
1500
1000
500

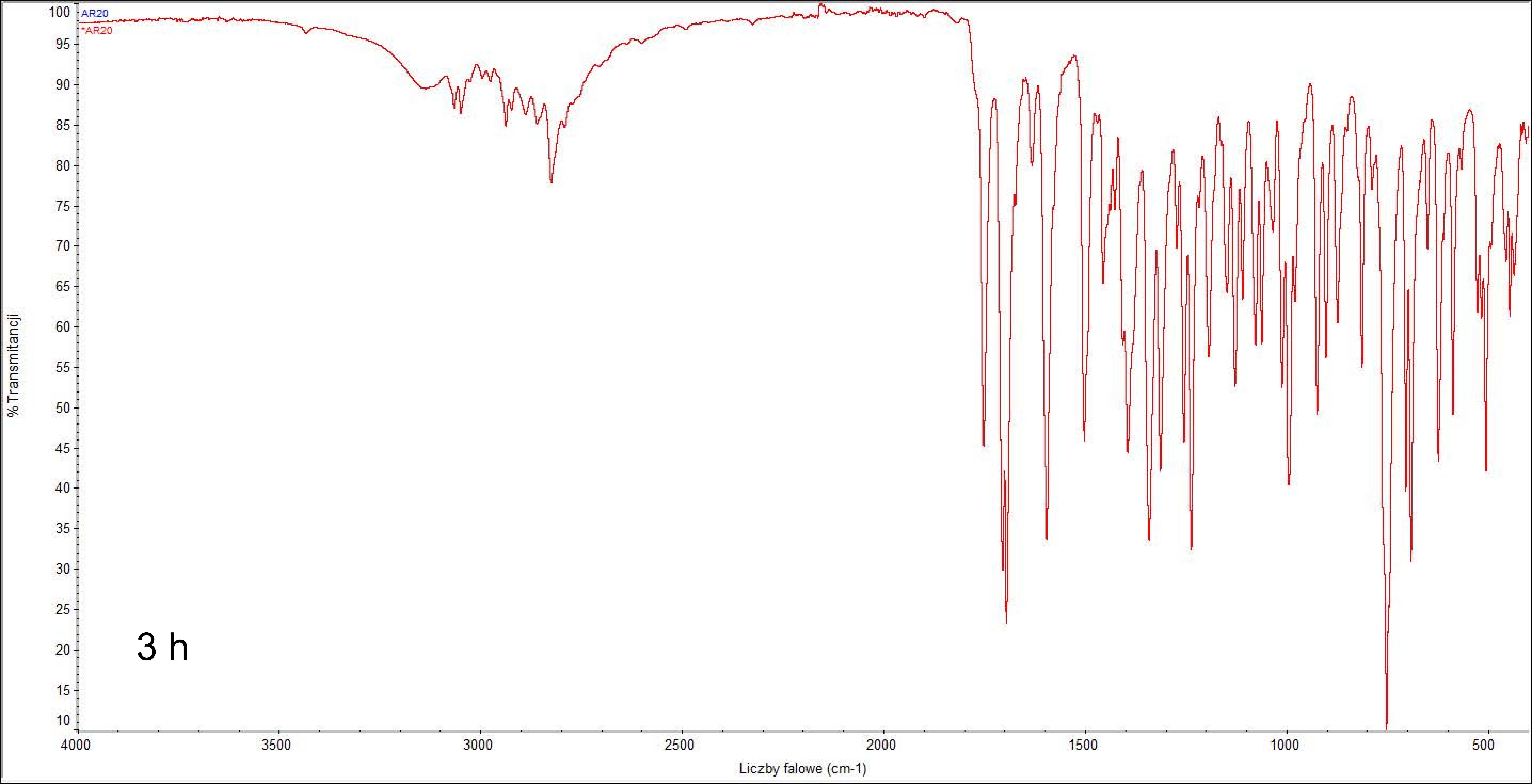

AR20
*AR20
% Transmitancji
Liczby falowe (cm-1)
3 h
100
95
90
85
80
75
70
65
60
55
50
45
40
35
30
25
20
15
10
4000
3500
3000
2500
2000
1500
1000
500

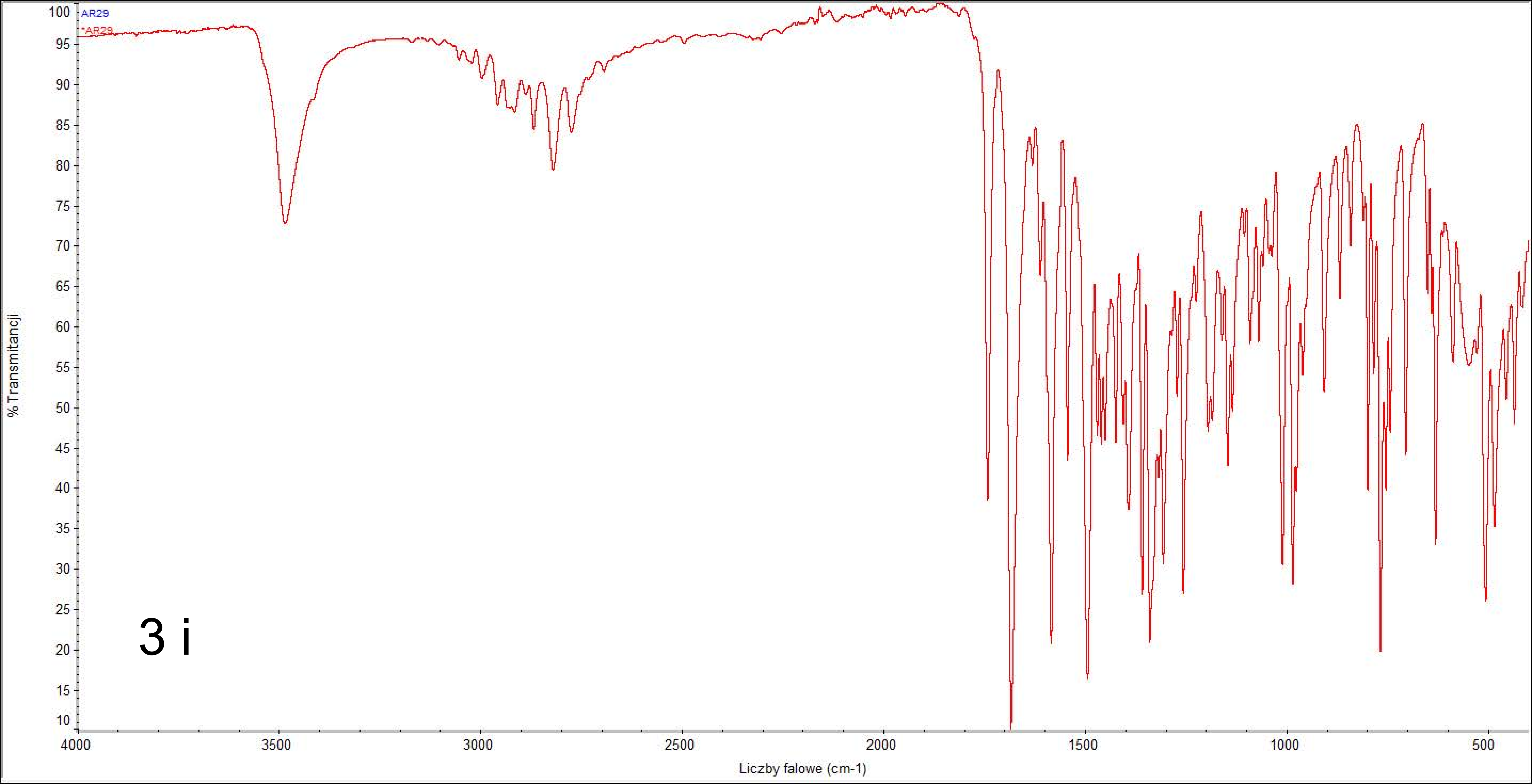
AR29
*AR29
%Transmitancji
Liczby falowe (cm-1)
3 i

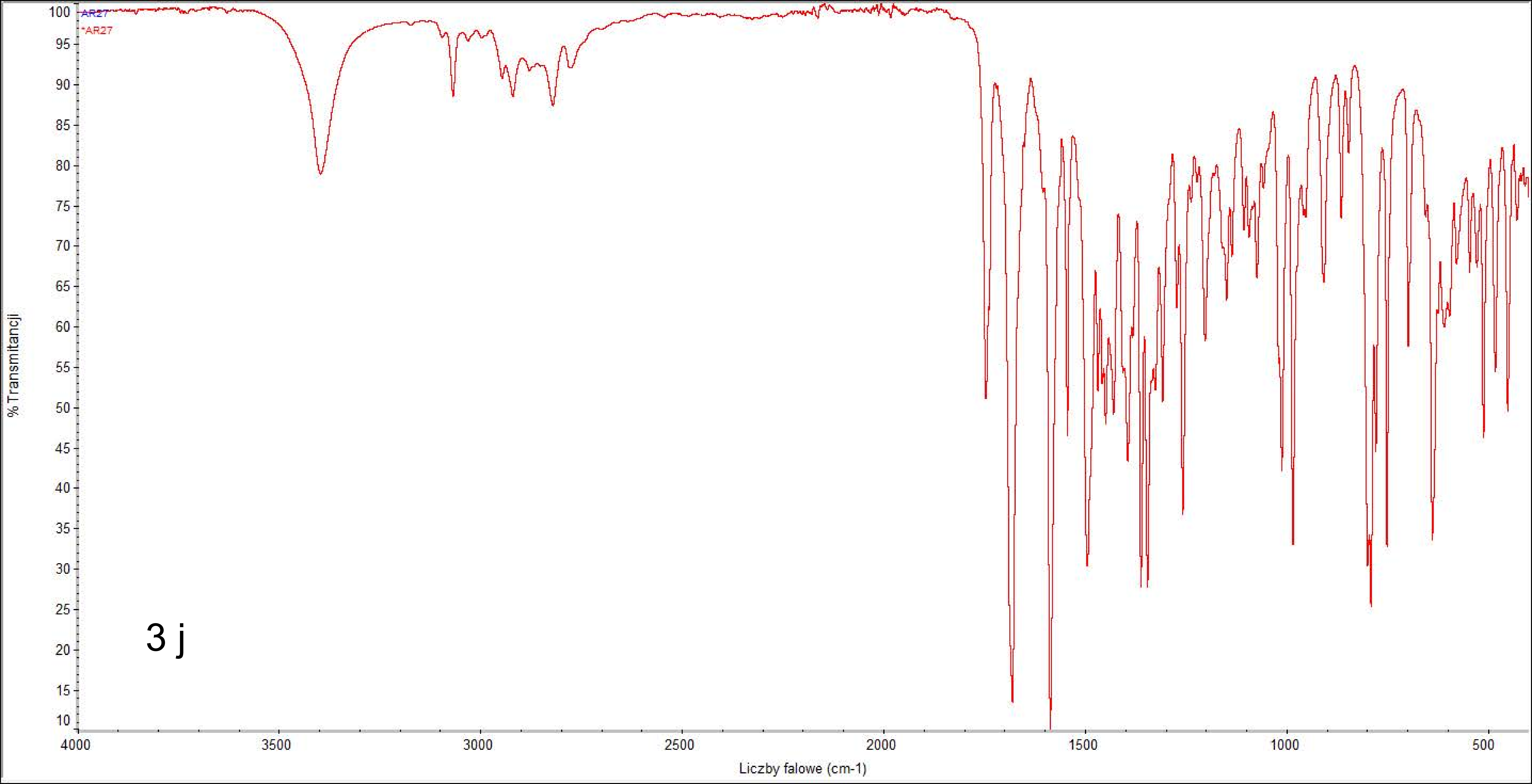

AR27
*AR27
% Transmitancji
Liczby falowe (cm-1)
3 j

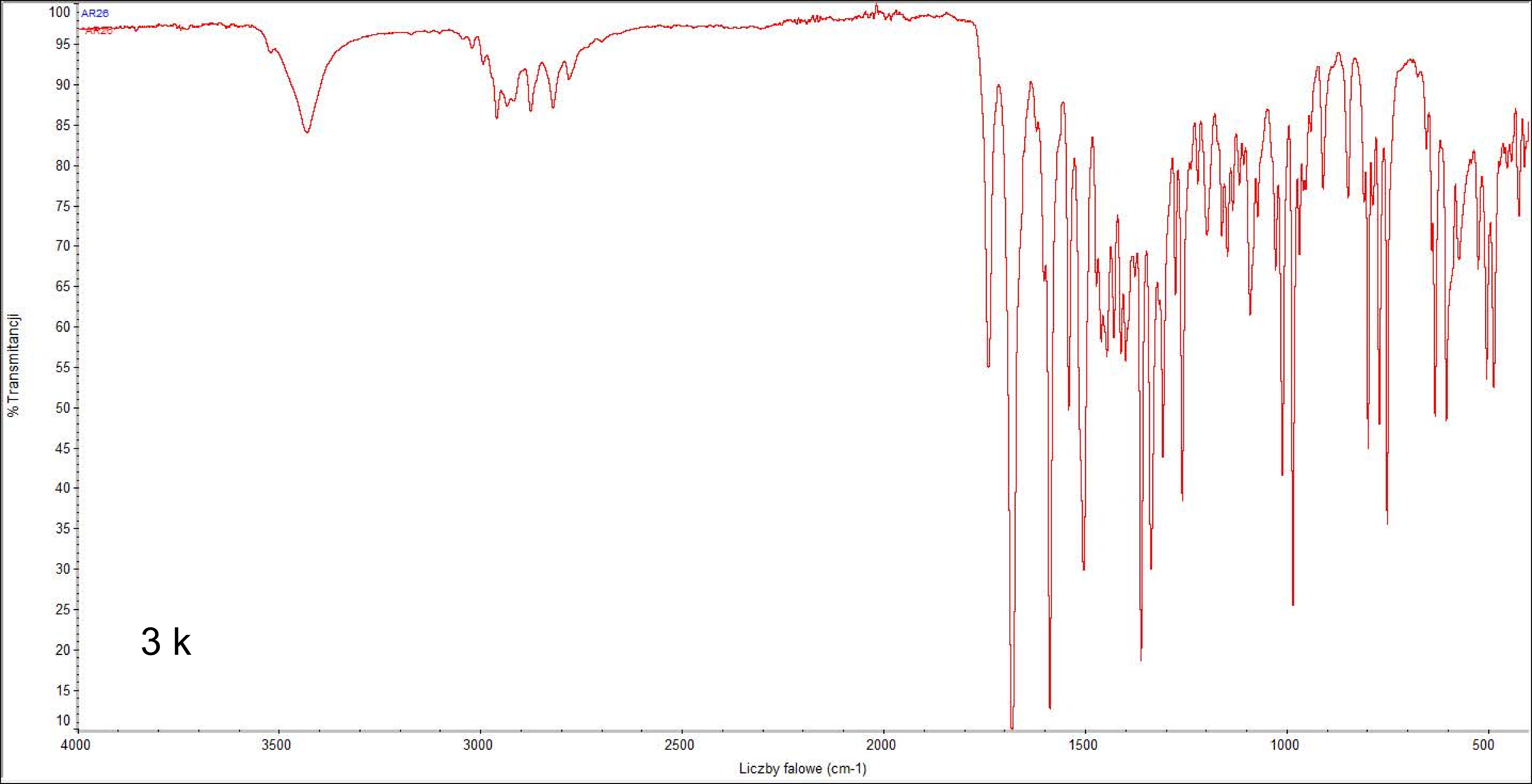
AR26
% Transmitancji
Liczby falowe (cm-1)
100
95
90
85
80
75
70
65
60
55
50
45
40
35
30
25
20
15
10
4000
3500
3000
2500
2000
1500
1000
500
3 k

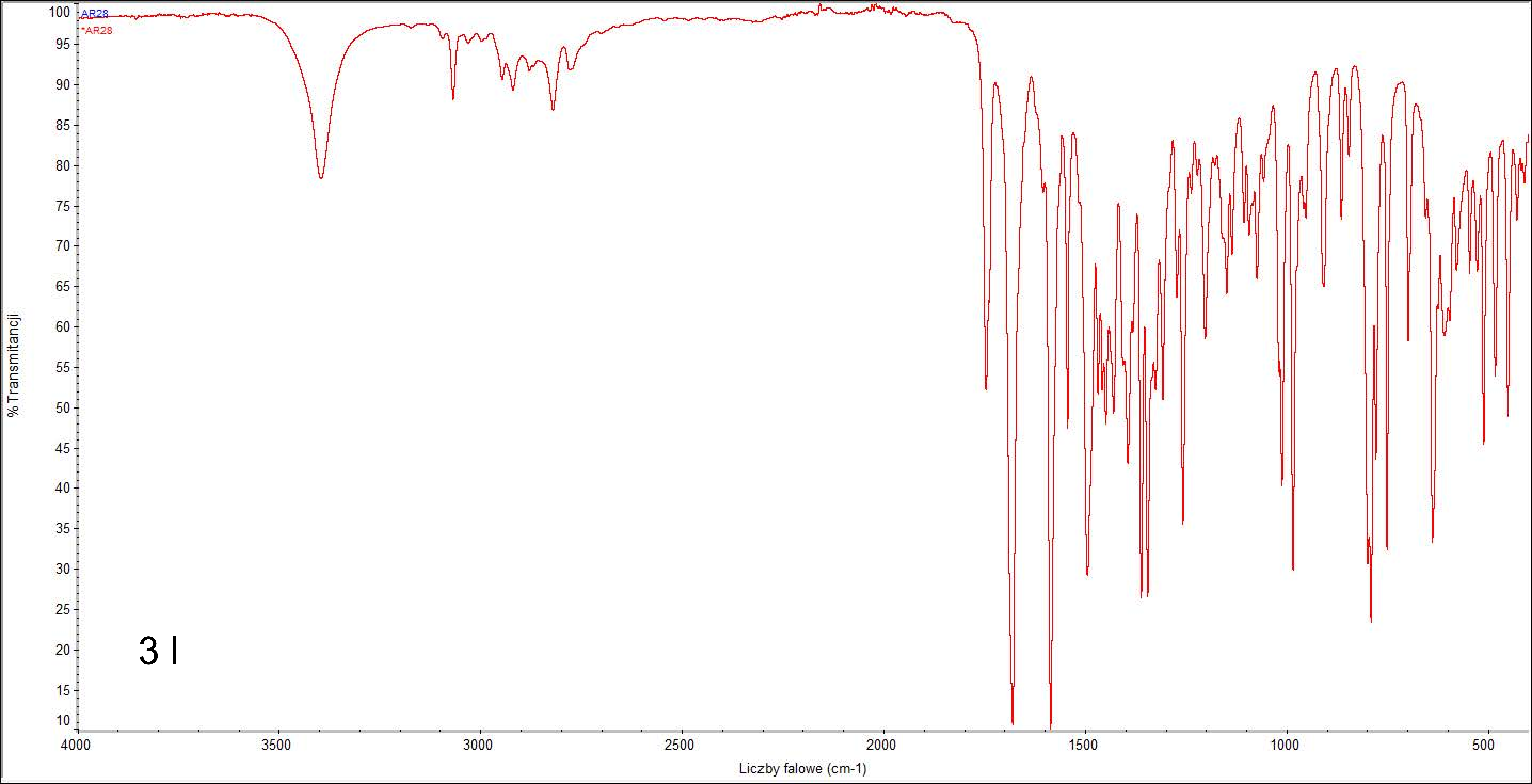

AR28
*AR28
% Transmitancji
Liczby falowe (cm-1)
3 l

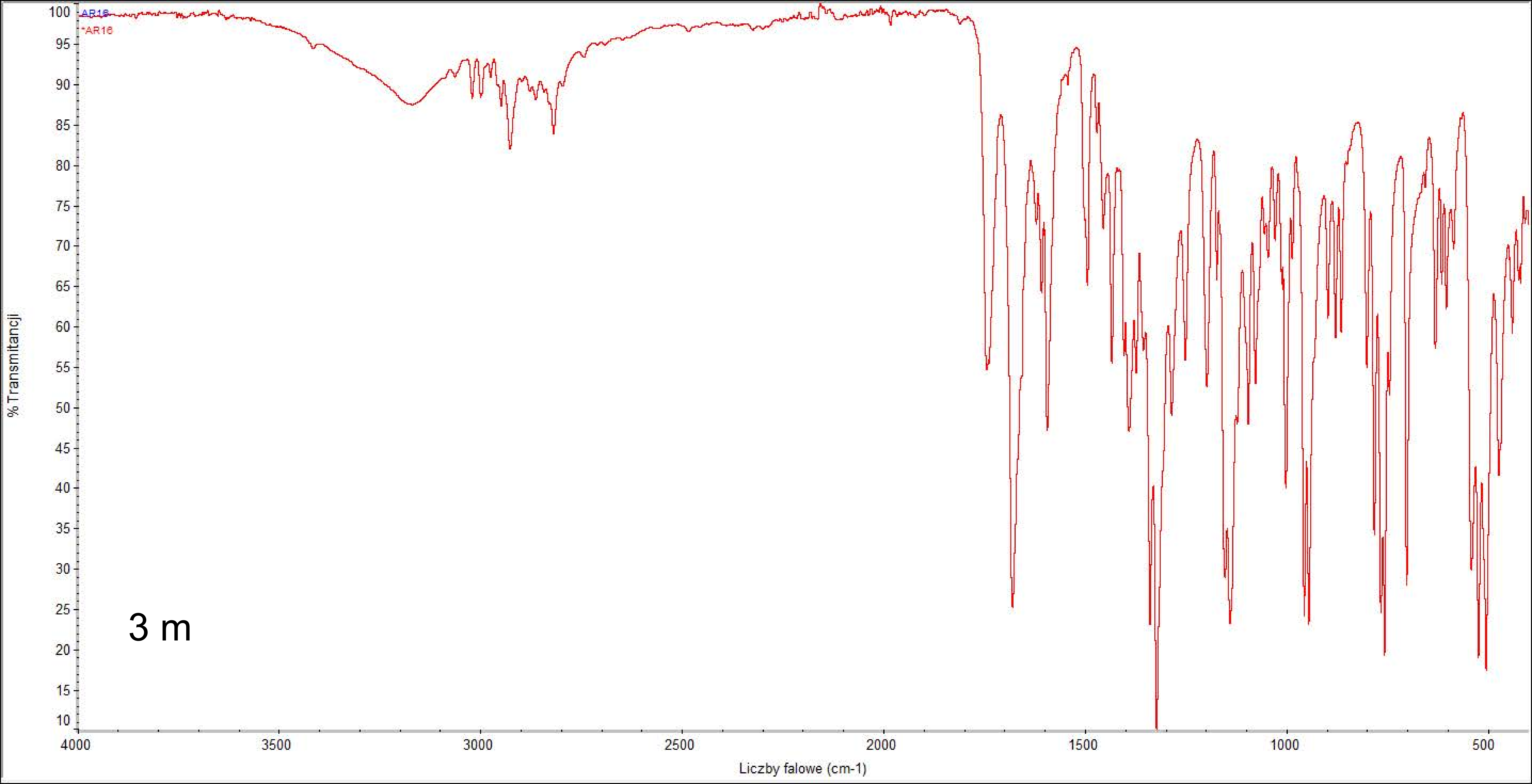

AR16
*AR16
% Transmitancji
Liczby falowe (cm-1)
4000
3500
3000
2500
2000
1500
1000
500
100
95
90
85
80
75
70
65
60
55
50
45
40
35
30
25
20
15
10
3 m

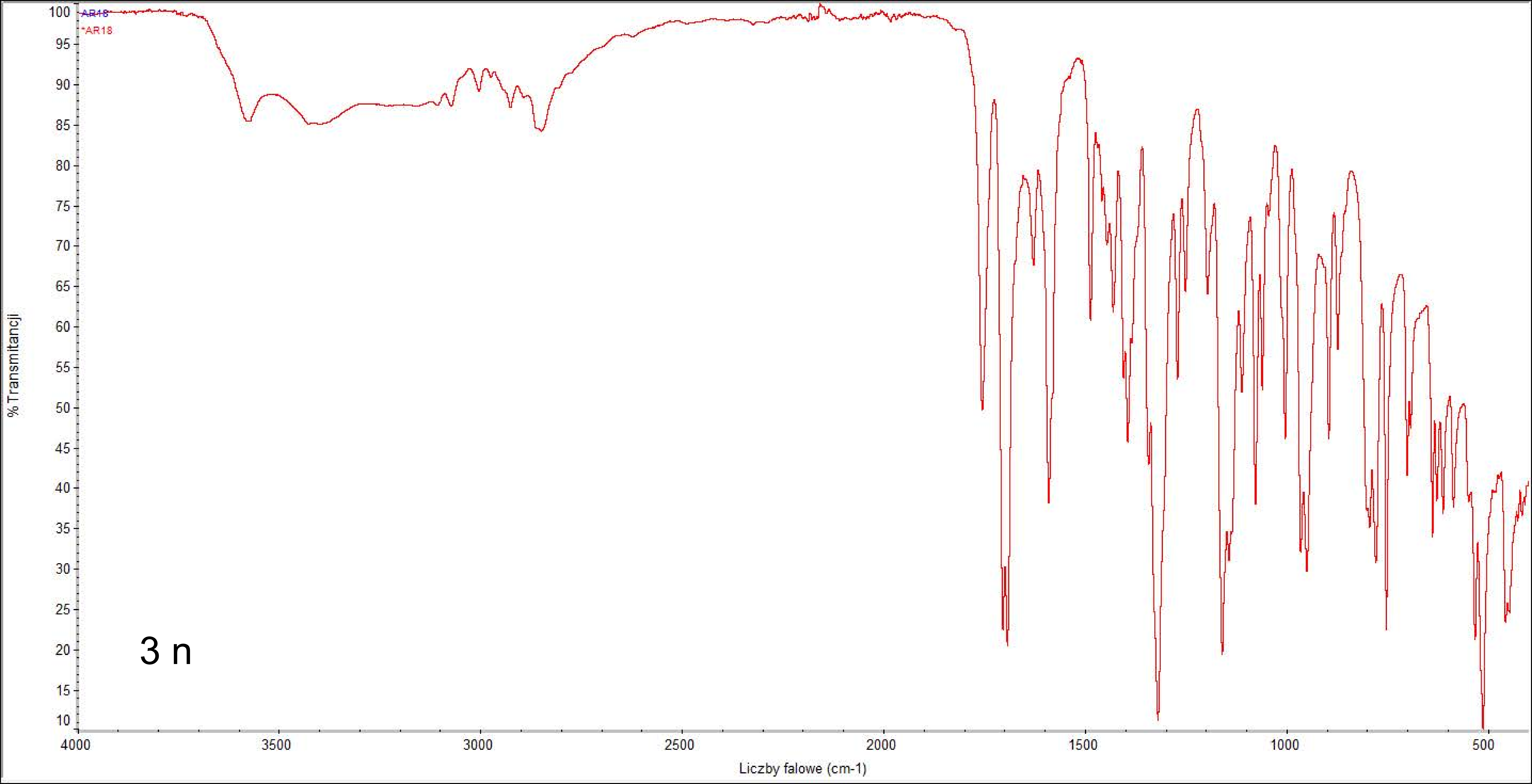
AR18
*AR18
%Transmitancji
Liczby falowe (cm-1)
3 n
100
95
90
85
80
75
70
65
60
55
50
45
40
35
30
25
20
15
10
4000
3500
3000
2500
2000
1500
1000
500

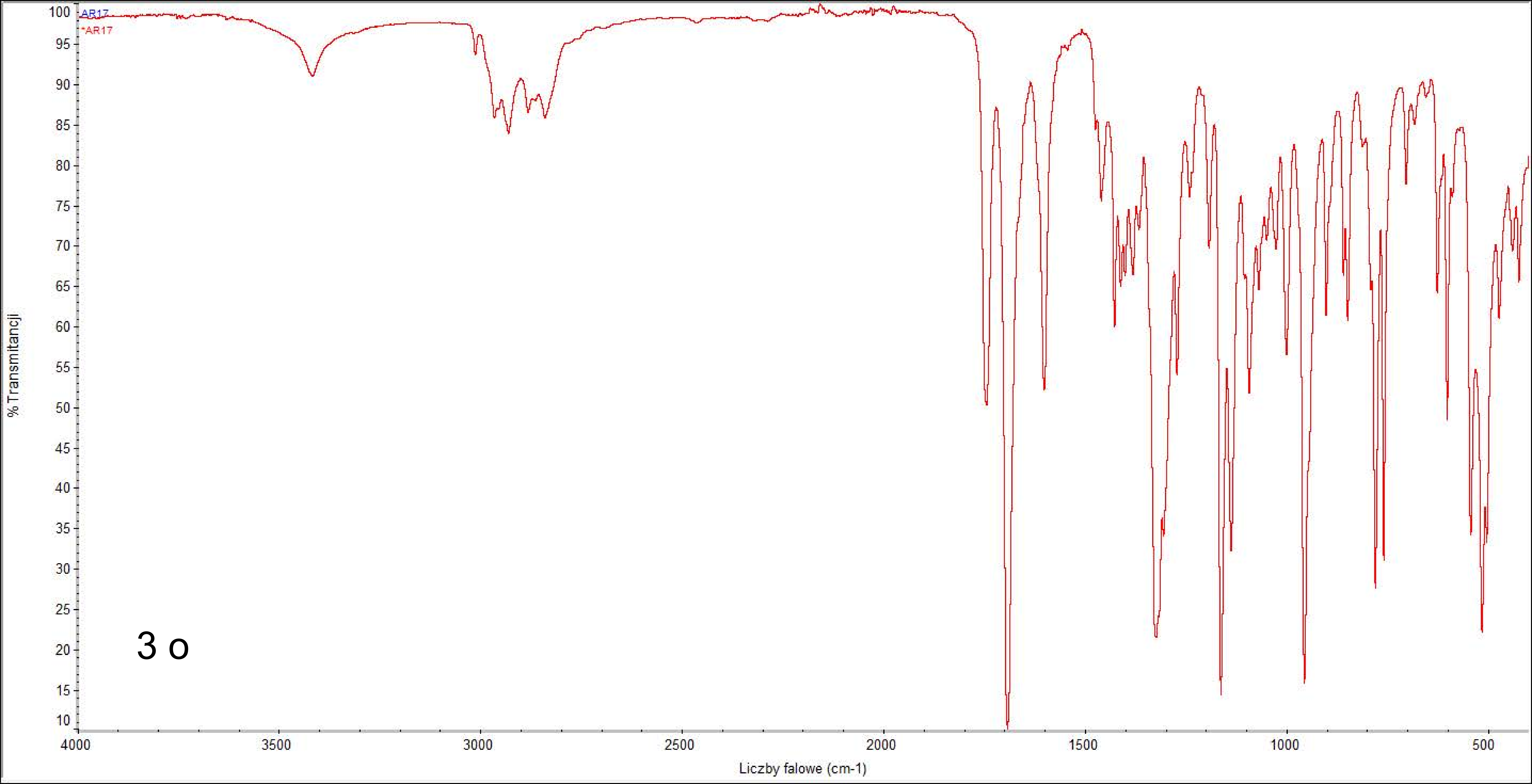
AR17
*AR17
% Transmitancji
Liczby falowe (cm-1)
100
95
90
85
80
75
70
65
60
55
50
45
40
35
30
25
20
15
10
4000
3500
3000
2500
2000
1500
1000
500
3 o
